# A green-footprint approach for parallel multiclass analysis of contaminants in roasted coffee via LC-HRMS

**DOI:** 10.1007/s00216-024-05157-4

**Published:** 2024-02-13

**Authors:** Julio César España Amórtegui, Susanne Ekroth, Heidi Pekar, Jairo Arturo Guerrero Dallos

**Affiliations:** 1 Chemistry Department, Science Faculty, Universidad Nacional de Colombia, Bogotá, Colombia; 2 Science Department, Swedish Food Agency, Uppsala, Sweden

**Keywords:** Ochratoxin A, Acrylamide, Pesticides, Stable isotope labeling, Validation, Eco-friendly

## Abstract

The development and validation of a simple, comprehensive, and environment-friendly procedure to determine pesticide residues, naturally occurring and processing contaminants in roasted coffee is presented. A solid-liquid extraction of pesticides and mycotoxins with ethyl acetate and the concurrent partition of acrylamide to an aqueous phase follows a parallel analytical strategy that requires a single analytical portion to determine contaminants that are typically analyzed by dedicated single residue methods. The partition rules the lipids out of the aqueous extract before an “in-tube” dispersive solid phase microextraction (dSPME) for acrylamide retention. This is followed by the elution with buffer prior to injection. This extract is independently introduced into the system front end followed by the injection of the compounds from the organic phase, yet all spotted in the same run. A novel liquid chromatography high-resolution mass spectrometry (LC-HRMS) method setup enables the quantification of 186 compounds at 10 µg/kg, 226 at 5 µg/kg, and the acrylamide at 200 µg/kg for a total of 414 molecules, with acceptable recoveries (70–120%) and precision (RSD < 20%) making this strategy significantly faster and cost-effective than the dedicated single residue methods. Even though the presence of chlorpyrifos, acrylamide, and ochratoxin A was confirmed on samples of different origins, the findings were below the limit of quantification. During the storage of raw coffee, no proof of masking of OTA was found; however, condensation with glucose was evidenced during thermal processing experiments with sucrose by using stable isotope labeling (SIL). No detected conjugates were found in roasted nor in commercial sugar-added torrefacto samples, an industrial processing usually carried out above the decomposition temperature of the disaccharide.

**Graphical abstract:**

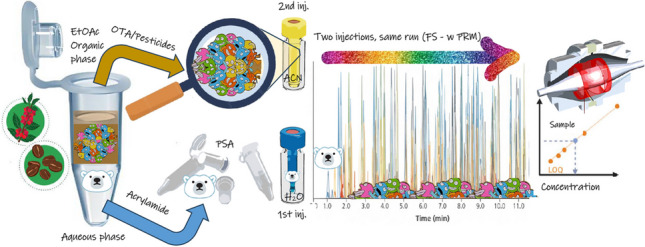

**Supplementary Information:**

The online version contains supplementary material available at 10.1007/s00216-024-05157-4.

## Introduction

*Coffea arabica* (arabica coffee), *C. canephora var. robusta* (robusta coffee), *C. liberica* (liberica coffee), and *C. liberica var. dewevrei* (excelsa coffee) are examples of species of significant importance in the coffee market. Besides, since a universally appreciated taste has built confidence in this everyday beverage key, controls must deal with potential contaminants coming from post-harvest and processing. Mycotoxins are a diverse group of highly toxic secondary metabolic products from various fungal species that could be considered ubiquitous in the environment. Once mycotoxins infect the field, it may end up in ripened beans [1], then develop during processing stages and resist even harsh conditions such as the coffee roasting [2]. In this regard, small-scale operators must pay attention to programs intended to ensure adherence to good practices to avoid mold formation in the first place [3]. Nevertheless, infestation during storage must be addressed too, since may lead to potential risks out of reach of coffee growers, harnessing an industry built on supreme quality. In fact, recommendations addressed to mitigate these risks may face challenges during the extension of storage conditions in situations such as the pandemic, the global trading crisis, or the rush dynamics of the market, severely hampering exports of coffee [4, 5]. Ochratoxin A (OTA) is the most toxic variant of ochratoxins and represents a serious risk to animal and human health [6]. Even though the most effective way to prevent OTA contamination is to ensure a moisture content of < 12.5% [7, 8], in some regions, coffee is sold wet (> 50%) mainly due to the lack of infrastructure (electrical dryers vs. sun drying). An example of this situation was a study dealing with strict humidity controls that showed no significant differences in the mold load for wet coffee (35–45%) after 96 h until drying [9]. Nonetheless, keeping moisture in coffee bags during transport may end up in a drop in quality, not only due to delays through rudimentary roads across the high mountains, but also in the long run, during storage and transport overseas. Since food containing a contaminant in unacceptable amounts shall not be placed on the market [10], the Commission Regulation 1881/2006 set maximum levels of contaminants, including mycotoxins, and amendments enforced some critical pollutants [11–13]. Likewise, EC 105/2010 [14] and EC 594/2012 [15] included a maximum level of OTA in products not considered in former regulations. This was recently updated to lower down the level to 3 µg/kg in roasted coffee [16]. The methods based on extraction and cleanup by immunoaffinity columns to analyze OTA in coffee are mainstream; for more than 20 years, there has been only one update on the European standard under the same work scheme [17, 18]. The European Food Safety Authority (EFSA) policies cover all matters on Food and Feed safety in the EU and have indicated that modified forms of mycotoxins can add substantial amounts to the levels detected in the product, since may contribute to overall toxicity. The occurrence of a handful of modified forms of toxins is being studied since it became critical to understand exposure [19]. Nonetheless, the potential OTA-modified forms have not been considered in the legislation so far [20]; therefore, it is imperative to dig deeper to understand the potential for any transformed forms arising from pre-existing contamination during long-term storage. This is why looking for the modifications that OTA might undergo is of critical importance since, if found, it has the potential to release the toxin from the matrix or end up hydrolyzed into the active moiety. This situation would lead to values above limits, even if the product is labeled as compliant with well-established routine methods. In addition, the market trends supporting the differentiation of organoleptic characteristics in the serving portion are promoting changes in the standardized protocols as consumers call for novelty. This made popular the trade of the dried cherry including the outer skin, that is, without any husking (natural) and also without removing the layers of husk and endocarp “pulp” from the beans or hulling (honey). Such crafted varieties are usually found in specialty coffee markets where the dealers might have extra profit from exotic nuances derived from the partially untouched coffee. However, deviations from the coffee standard for exportations (washed) might keep moisture beans for longer. Therefore, potential hazards derived from OTA modifications under long-term storage conditions must also be addressed in these alternative types of raw “green” coffee, as well as during the thermal treatments taking place at well-known industrial coffee factories. Upgrading facilitates producers’ access to the high-value coffee market and is often associated with higher farm income, since consumers pay a price premium for the higher quality [4]. An example of that is that now farmers also own small-scale roasting houses, allowing them to get the most from their production. However, while the thermal process occurs, green beans start turning brown and a number of complex reactions in the bean take place. Along with several Maillard reaction byproducts, acrylamide (AA) is also created in the process. Evidence of the presence of this carcinogenic compound was published for the first time in starchy products [21]; then, it was detected in ground and instant coffee appearing after the thermal treatment which is mandatory to release its particular taste and flavor [22]. Benchmark levels (BML) for the reduction of the presence of acrylamide in food were then established as the average content in coffee at 400 µg/kg according to the report of Commission Regulation (EU) 2017/2158 [23].

Generally speaking, pesticides are used in coffee crops in order to avoid diseases affecting the quality and quantity of production. In the final product, the residues might find their way to the consumer, and even though only some crop protection products are registered for coffee, any eventual misuse or simply the runoff from the steep hills adjacent crops make the screening for contaminants essential in order to comply with regulations. The analysis of hundreds of pesticides can be challenging because the physicochemical properties of these compounds vary widely. From research to routine, recognizing the actual analytes among endogenous compounds in this complex matrix may become a challenging task. Laboratories usually invest resources in different single residue methods to address OTA and AA separately; however, a multiclass residue method could be a great asset for fast decision-making. Though triple quadrupole (QqQ) brings unmatched sensitivity [24], identification and confirmation using a combination of acquisition modes based on high-resolution mass spectrometry (HRMS) may bring further confidence, the key to a wider scope along with the potential of retrospective analysis [25, 26]. In addition, control bodies continuously seek new methods to accurately identify hazards and verify compliance, with a focus on simplifying sample preparation where possible. Particularly, green chemistry metrics [27] are considered crucial in method development. The analytical Eco-Scale is a semiquantitative approach; however, it only has to do with the impact of the solvents used [28]. On the flipside, GAPI is a comprehensive method assessing the entire workflow, yet most of the methods do not cover the synthesis before the sample preparation. In the pursue of greener approaches as an alternative to current methods that use large amounts of toxic solvents and waste per sample, it is key to foresee an ecofriendly approach and evaluate as many aspects as possible during the development to select the greenest available solution in different regards to mitigate the impact the analytical method inevitably poses on the environment. This study aims to explore threats across the different coffee stages and hence develop a relevant, comprehensive, and fit-for-purpose approach to key contaminants in roasted coffee that from an environmentally friendly perspective could encompass a multiclass scope.

## Materials and methods

### Materials and samples

#### Chemicals, standards, and consumables

High-purity ochratoxin A was purchased from Sigma-Aldrich (Saint Louis, USA) and prepared at 500 µg/mL in LC-MS grade acetonitrile (ACN). Aflatoxin mix solution (2 µg/mL B1 and G1, 0.5 µg/mL B2 and G2, in ACN) procured from Merck KGaA (Darmstadt, Germany). Isotope-labeled internal standards (ILISs) of acrylamide-d_3_ (98%) from Polymer Source Inc. (Dorval, Canada) and acrylamide-^13^C (99%) from CDN Isotopes Inc. (Pointe-Claire, Canada) were prepared at 10 mg/mL in HPLC grade water and stored at − 20 °C. Ochratoxin A—^13^C_20_ (10 µg/mL in ACN) was purchased from Sigma-Aldrich (Saint Louis, USA). Atrazine-d_5_ (99.0%) was purchased from Merck KGaA (Darmstadt, Germany) and prepared at 10 µg/mL in LC-MS grade ACN and stored at − 20 °C. A working solution with approximately 700 compounds was prepared at 1 µg/mL out of five commercially available stock standard mixtures from the Smart Solutions™ v700 PestiMix standard kit from Dr. Ehrenstorfer-LGC (North Charleston, USA), originally at 5 µg/mL and stored at − 20 °C. The final multi-analyte standard was freshly prepared prior to spiking experiments by mixing the intermediate working solutions to dilute them down to 50 ng/mL to spike with reasonable volumes (i.e., at 10 µg/kg with 10 µL). All spiking solutions were stored at − 20 °C. LC-MS grade methanol (MeOH) was purchased from Scharlau (Barcelona, Spain); residue grade ethyl acetate (EtOAc) and LC-MS grade ACN were acquired from J.T Baker (Radnor, USA). Type-I grade water (18.2 MΩ) was produced from a water purification system Direct Q from Merck (Darmstadt, Germany). Gallic acid was purchased from Merck Schuchardt OHG (Hohenbrunn, Germany), D (+)-glucono-1,5-lactone from Alfa Aesar (Ward Hill, MA, USA), D (-)-sorbitol from Merck KGaA (Darmstadt, Germany), and sucrose from Supelco (Bellefonte, PA, USA). The modified primary secondary amine silica sorbent, Bondesil® PSA (ethylenediamine-N-propyl), was purchased from Agilent technologies (Santa Clara, USA) and the Isolute® ENV+ sorbent from Biotage (Uppsala, Sweden). LC-MS grade formic acid and ammonium formate were acquired from Scharlau (Barcelona, Spain), ammonium carbonate from Merck KGaA (Darmstadt, Germany), Spin-X® centrifuge tube filters were purchased from Corning (Glendale, USA), and Sorton poliamide filters (0.2 µm pore diameter, 13 mm) were purchased from Sartorius Stedim Biotech GmbH (Goettingen, Germany).

#### Samples

The department of Tolima in Colombia has the advantage of having a main harvest and a second harvest called “mitaca,” less abundant, yet interspersed throughout the territory. The raw samples were collected in January 2022 in rural farms near the municipality and labeled accordingly. The three regions are located in separate areas at different altitudes. The following coordinates and elevation show the origin of the samples collected: San Juan de la China at 4° 32′ 27.6″ N 75° 04′ 30.0″ W (SJ), 2000 m.a.s.l.; San Antonio at 3° 55′ 12.0″ N 75° 29′ 24.0″ W and 1500 m.a.s.l. (SA); and Libano at 4° 54′ 43.6″ N 75° 00′ 12.2″ W, 1400 m.a.s.l. (LB). From each location, three different types of coffee beans representing differential processing or *beneficio* were sampled: natural (N), honey (H), and washed (W). The proficiency test (PT) items from rounds of the FAPAS® Food Chemistry proficiency testing scheme samples of ground and instant coffee were acquired from Fera Science Ltd (York, UK), stored at − 20 °C until use, and not subjected to any particular pre-treatment and were used “as is.” Torrefacto is a roasting process in which sugar is added to influence bitterness and mask negative flavors [29–32]. Commercial roasted and torrefacto coffee samples were purchased in whole beans at local markets from several locations, transported, and homogenized by a conventional milling procedure at ambient temperature shortly after package opening. These samples were stored at − 20 °C until use in the extraction and cleanup experiments and for validation purposes.

### Sample preparation and extraction

In the first part of the study, the evaluation included the different types of coffee (H, N, V) in order to identify any possible OTA conjugation during storage. The potential to release the mycotoxin may increase toxicity, so the target compounds are any transformation products of the OTA that retain the labeled atoms from a known source. To achieve this, triplicate portions of 1 g (among 10 beans on average) of each coffee were spiked at 5 µg/kg with both the non-labeled OTA and labeled OTA-^13^C_20_; in a similar fashion, other studies carried out experiments using isotopic labeling techniques [33–36]. The spiked concentration matched the level under regulation by the time the experiment started. A solution containing the corresponding solvent fraction was added and used as a control and to screen for any pre-existing contamination (mock-treated sample). In addition, two quality controls (QCs) spiked at 250 µg/kg and a diluted version (dQC) at 25 µg/kg were included in order to account for the low concentration used during the experiment that may hamper detection. The samples were placed in 500 mL conical flasks and left in the dark in a forced air oven under monitored conditions of moisture exposure (40–60) % and 40 °C for 6 months. After this period, the whole content was ground to a homogeneous powder left in glass vials in the freezer at − 20 °C until analyzed. The sample extraction was done the same day for the sample batch after the ambient temperature was reached. The ability of three solvents (ACN, MeOH, and water) and combinations thereof in extracting the three types of coffee was also investigated. A design of experiments (DOE) was used to obtain different combinations to perform extraction and compare in order to achieve a maximal extraction capacity. D-optimal designs are a sort of DOE method constructed to minimize the generalized variance of the estimated regression coefficients. This kind of design is convenient when limitations in the number of feasible experiments exist, or the experimental space might not be fully explored. In this case, restrictions on extraction solvent composition consist of a fixed 50% water to avoid ending up with a fully neat organic extraction and resemble the conditions in the beverage extraction at least to some extent. In this experimental plan, runs to be performed were selected from a multi-level full factorial design (FFD) [37] creating a matrix of candidate points shown in Table S1. In order to carry out a generic extraction and get different extraction profiles as comprehensive as possible, 200 mg of powder was taken into 50 mL polypropylene centrifuge tubes and then 10 mL of each of the labeled solvent mixtures (S1 to S10) was added per equal weighted samples. The ultrasound-assisted solid-liquid extraction took 10 min and then the tubes were vortexed for 1 min and centrifuged at 16,000 g at 4 °C. The raw extract was filtered through 0.2 µm pore 13 mm filters into an amber vial to add up to 90 spiked samples (3 coffee types, 3 locations, 10 extraction compositions), 90 corresponding blanks, and both QCs ready for LC-HRMS acquired with Method 1, explained later in the “Instrument conditions” section. Blanks and randomized samples were injected along to neat solvent at regular intervals within the sequence (every 10 injections). Injection of the dilution of QCs helped to verify the system response remained stable during the sequence.

With the aim of choosing a given composition for extraction, the effect of the different solvent mixtures on the OTA extraction was assessed. Besides, taking advantage of the ideally unbiased untargeted capabilities in LC-HRMS, the matrix profiles of green and roasted coffee commercially obtained were also evaluated in another comparative design but only using the chosen composition for extraction. However, in many untargeted plant metabolomics studies, solvent mixtures with or without acidification are used for sample extraction showing differential results [38], so that the effect of acidification of the extraction solvent with 0.1% formic acid was assessed too. In addition to this, the use of sorbents for cleanup was also part of this small experimental design. For instance, highly effective solid phase extraction (SPE) sorbents such as Isolute® ENV+ and Bondesil® PSA were evaluated concomitantly with the acidification and over both matrices. Therefore, by using the same extraction quantities to obtain raw extracts with the chosen solvent mixture in the D-optimal design, 500 µL of both the non-acidified and acidified extracts were put into different Eppendorf tubes containing 50 mg of each sorbent, respectively. The dispersive SPE cleanup was conducted by vortex for 1 min and centrifuged at 16,000 g at 4 °C. The raw extract was filtered through 0.2 µm pore 13 mm filters into an amber vial for injection in LC-HRMS using the acquisition Method 2. Finally, the last part of the experiments that relied on an untargeted approach dealt with potential OTA-modified forms during intense heating and included two types of roasted coffee, that is, regular roasted and torrefacto coffee. The formation of OTA conjugates was first assessed by heating each of the model molecules (gallic acid, D (+)-glucono-1,5-lactone, D (-)-sorbitol, and sucrose) in a mixture with OTA at 175 °C as described elsewhere [39] followed by LC-HRMS via acquisition Method 1. Afterwards, a heating experiment only for sucrose was performed using the artificially contaminated coffee matrix; then, it was extracted using MeOH/H_2_O (1:1) and analyzed with the acquisition Method 3 to identify products from preselected precursors. Finally, a variation of Method 1 was used to monitor the selected signals in a stepped heating setup and to analyze roasted and commercial torrefacto samples.

**Fig. 1:**
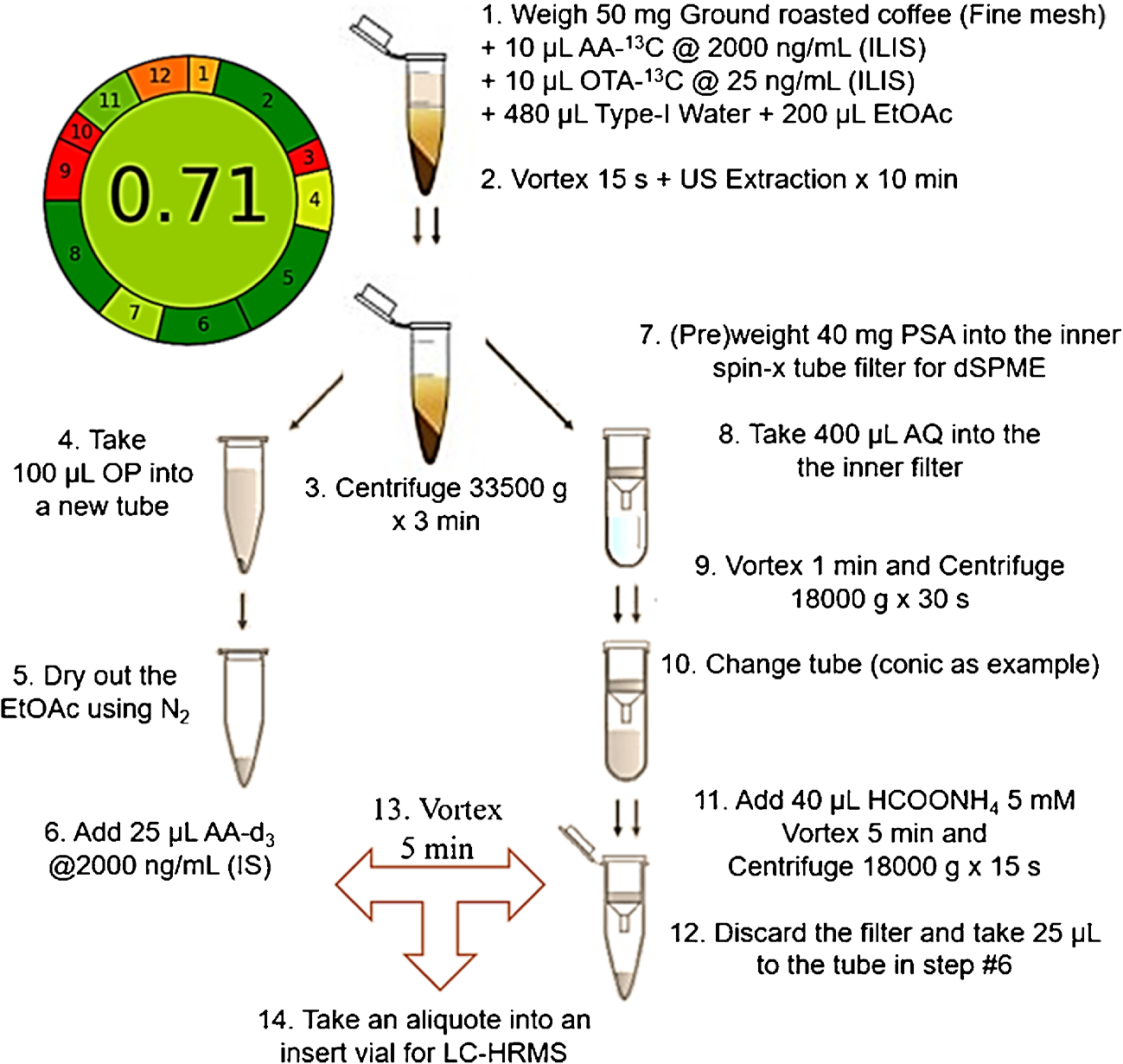
Workflow scheme for the determination of acrylamide and ochratoxin via “in-tube” dispersive solid microextraction (dSPME). AA acrylamide, OTA ochratoxin A, EtOAc ethyl acetate, US ultrasound, PSA primary secondary amine modified sorbent, OP organic phase, AQ aqueous phase. Analytical Greenness metric (AGREE): 0.71, under the 12 principles of green chemistry [40]

In the interest of including the method scope of relevant contaminants in coffee, the determination of acrylamide in roasted coffee was considered in a separate procedure depicted in Fig. 1. The steps in the “Sample preparation and extraction” section method are explained as follows. Weigh 50 mg of roasted ground coffee into an Eppendorf tube, then add 10 µL of AA-^13^C at 2000 ng/mL and 10 µL of OTA-^13^C at 25 ng/mL as I.L.I.S. for quantification of the non-labeled counterparts. Add 480 µL of Type-I grade water and 200 µL of EtOAc, close the lid firmly, and manually shake it to distribute evenly before vortex for 15 s, then place the tube into an ultrasonic bath (US) for 10 min. Centrifuge the blend per 33,500 g for 3 min at 4 °C. A phase separation is visible when centrifugation is over, then place 100 µL of the upper organic phase (OP) into a new Eppendorf tube and let it dry using a gentle stream of nitrogen under the hood; once the residue is completely dry, add 10 µL of AA-d_3_ at 2000 ng/mL (I.S.) and reserve the tube for later use. Regarding the aqueous phase left in the bottom, take 400 µL avoiding any residual solvent to enter upwards the pipette tip by blowing some air while crossing the liquid surface. Place the aliquot on top of 40 mg of pre-weighted Bondesil® PSA placed into the Spin-X® filter for the “in-tube” dispersive solid phase microextraction (dSPME). Close the lid and vortex for 1 min, then centrifuge per 18,000 g for 30 s at 4 °C. Open the Spin-X® tube lid and take out the filter only to place it into a new Eppendorf tube (a conic tube illustrates that it is a new tube). Add 40 µL of ammonium formate at 5 mM on top of the sorbent and vortex for 5 min, then centrifuge per 18,000 g for 15 s at 4 °C. Discard the filter and take 25 µL to the tube previously reserved. Close the lid and vortex for 5 min making sure the contents are moving all over the walls of the tube. Finally, take an aliquot larger than 40 µL into an insert vial for LC-HRMS using the acquisition Method 3.

Finally, in order to expand the scope of the method, it could be insightful to include pesticides and other mycotoxins under surveillance. For this reason, the above-mentioned method for roasted coffee was modified in three aspects: spiking, vial preparation, and the addition of a “User Defined Program” (UDP) for the automatic injection. Firstly, the spiking scheme included a mix of aflatoxins B1/2 and G1/2, and a working solution with approximately 700 compounds at 5 µg/kg, 10 µg/kg, and 20 µg/kg for validation purposes. In addition, atrazine-d_5_ was used as a surrogate compound at a fixed concentration of 10 µg/kg. Secondly, step no. 13 of the sample preparation in Fig. 1 is not performed and both the aqueous-based and the organic-based phases are injected in separate vials as explained hereby. In one hand, add 50 µL of ACN after step no. 5 to the dried Eppendorf and vortex for 1 min, then place an aliquot larger than 40 µL into an insert vial labeled with the identifier “OP” for the organic phase and place it into the sample tray of the LC-HRMS. On the other hand, take 25 µL from the bottom of the Eppendorf in step no. 12 and place it into another separate insert vial with 25 µL with ammonium formate at 5 mM, preferably labeled with the identifier “AQ.” The two vials are then placed in adjacent positions into the autosampler to perform the UDP injection routine that includes the sandwich injection of the TPP along the AQ-based sample to start the acquisition followed by the injection of the OP-based sample. This procedure is more comprehensive and was the method subjected to the in-house validation trial.

### Instrument conditions

Chromatographic separation followed by mass spectrometry analysis was performed in a UHPLC Dionex™ Ultimate 3000 (Thermo Scientific™, San Jose, CA, USA) coupled to a Q Exactive™ Focus mass spectrometer (Thermo Scientific™, Bremen, Germany). Mobile phase A and mobile phase B consisted of a mixture of 98% water + 2% MeOH and 98% MeOH + 2% water, respectively. Additives for both mobile phases consisted of 5 mM ammonium formate and 0.1% formic acid was added to enhance ionization. Separation was performed in a C18 stationary phase UHPLC column Accucore aQ (Thermo Scientific™, San Jose, CA, USA) of length, diameter, and particle size of 100 mm, 2.1 mm, and 2.6 µm, respectively. A precolumn with the same stationary phase and corresponding dimensions was attached to the column and a 100 µL loop connecting the precolumn to the autosampler valve used to improve the peak shape of early eluting compounds. The column was set at 30 °C during the analysis. The flow used was 300 µL/min, the injection volume was 10 µL, and the autosampler was cooled at 10 °C. The gradient started with 100% A. After 1 min, the percentage of B was increased to 40%, followed by another increment to 70% at 3 min, and finally reached 100% at 7 min. The latter composition was additionally kept for 5 min and then steeply dropped to 0% in 0.1 min; finally, this was kept for 2 min to allow column equilibration for the next run; the total runtime was 15 min. The mass spectrometer was equipped with a heated electrospray ionization source (HESI II). The HESI II source parameters were optimized elsewhere [41] and set as follows: spray voltage of 3.5 kV in positive mode, sheath gas flow rate 35 arbitrary units (au), auxiliary gas flow rate 12 au, sweep gas flow rate 0 au, capillary temperature of 210 °C, auxiliary gas temperature of 350 °C, and S-lens radiofrequency of 50. An external mass calibration was performed daily with a mix of n-butylamine, caffeine, Ultramark 1621, and MRFA. The data analysis was performed using the software TraceFinder v4.0 (Thermo Scientific™, San Jose, CA, USA), if not stated otherwise. The data acquisition settings in the Orbitrap analyzer varied with the type of experiment and can be found described in Table S2 as Polarity switching—Full Scan (FS) acquisition coded as “Method 1,” Full Scan – Data dependent MS2 (FS-ddMS2) as “Method 2,” Parallel Reaction Monitoring (PRM) “Method 3,” and Full Scan—wide Parallel Reaction Monitoring (FS-wPRM) or “Method 4.”

### Method validation

In order to perform the method validation of the “all-in-one” or comprehensive method, the guidance document on the identification of mycotoxins in food and feed SANTE/12089/2016 [42] was taken into account, and the whole quantitative validation was performed considering SANTE/11312/2021 [43]. The parameters tested were selectivity, linearity, trueness, and precision. To evaluate the trueness, precision, and limit of quantitation (LOQ), samples were fortified at three different concentrations, corresponding to 0.005, 0.010, and 0.020 mg/kg. In particular, OTA was also evaluated at 0.0025 mg/kg to assess method performance below the regulation levels in force. Samples of different origins and characteristics (robusta and arabica, roasted, and torrefacto) were used to assess the applicability of the method and its capability to deliver trusted results in some varieties of the specialty and international commercial coffee market.

## Results and discussion

### Investigations on potential ochratoxin A modifications during storage

Variations to the current standard process for green coffee have been recently applied in farms over the territory, leading to tailored methods such as the selected N, H, and V types referred to as “semidry” processing [44]. The mycotoxin transformation products are as important as the primary toxin and being part of the pool of analytes in the strategy might contribute towards a comprehensive food safety assessment. The term “modified mycotoxin” denotes different forms regardless of origin [20, 45], and considering that the levels of moisture of natural, honey, and washed types might be above the recommended levels during storage, a long-term study was planned to look for changes that may hide OTA from artificially contaminated samples. Once the 6-month period ended, the samples were subjected to analysis based on 10 different solvent extraction compositions in Table S1. The LC-HRMS analysis of the batch made use of the parameters described in the section “Instrument conditions” and Method 1 for acquisition. Raw files were analyzed by the MetExtract II software [46], a powerful tool that allows extracting features via the wavelet signal analysis [47] and with the added benefit of addressing “credentialed” features [48] using the TracExtract module through isotopic pattern recognition [49] by comparing the pattern with the one expected for the marked compound. Other than the AllExtract module, the suite is not designed to detect all metabolites of the system but will only report products of the studied tracer, thereby, endogen features not directly related with the treatment are ruled out. Figure S1 (a) shows locations (LB, SA, SJ), types (H, N, V), and overlayed peaks for all extraction mixtures with different compositions (S1–S10). In general terms, the matching signals in Figure S1 (b) reflect the 1:1 mixture of ^12^C/^13^C OTA. From all the results showing feature pairs reported by the software, neither most findings comply with the 20-carbon distance nor actual labeled peaks correspond to the native trace. To be able to credential a feature group properly, a peak from the labeled version must match the retention time at the specified distance on the *m/z* axis from the spectrum as shown in Figure S1 (c and d) for the QC, the corresponding OTA in both positive and negative polarities. Despite the use of the mixtures exhibiting different polarities, no OTA-modified forms from the semidry coffee types were found, even at the QC concentrations there were no complying feature groups in none of them, aside from the single OTA that was correctly identified by the TracExtract module. The metrics for the findings can be found in Figure S2. This may indicate the storage of artificially contaminated coffee beans under the experimental conditions did not lead to modified forms that supposedly remain undetected eventually releasing the OTA moiety.

It is worth mentioning that the D-Optimal design is used in cases in which classic designs do not apply [37] due to the natural interaction that is expected between the factors, just in the case of the components in the extraction solvent mixture [50]. Selecting an appropriate model is subjective by nature since there are plenty of parameters one might rely on to take the final decision. Often, the true functional form of the relationship between the response and the factor variables is unknown and depends on the experimental limitations of the planned design. Nevertheless, the corrected multiple *R*^2^ could be a good estimate to understand how the fourth order is the model that fits the best to the extracted OTA areas for each combination. This can be seen in the R output using *MixModel* function [51] in Figure S3 (a) where the studentized residuals show no visible trends (Figure S3 (b)). The red dots in Figure S3 (c) show the compositions from the matrix of candidate points and the contour lines and colored surface with the highest response. In a similar fashion, the results were analyzed using the statistical package DesignExpert™ v13 to build the 3D surface response in Figure S4 (a). The relative positions for each mixture are scaled to 1, and the ideal composition of the mixture is calculated by the “Desirability” function, shown with a white flag near 0.5 MeOH in Figure S4 (b) for simplicity. This result suggests that a mixture of MeOH and H_2_O (1:1) is the best of the options tested for the OTA extraction. Often, comparing total ion chromatograms (TIC), looking for weight-based differences in the residue left after evaporation or the color of the extract are warning signs repeatedly associated with “dirtiness” [52–55], these approaches have been of help in deciding among extraction methods and cleaning agents for years. However, the TICs from the different mixtures looked so similar that it was difficult to tell which looked “cleaner.” So that, with the aim of better evaluating the extracts in an unbiased way, the raw files were analyzed with the Compound Discoverer™ 3.3 software considering the factors studied. Figure S5 shows a scatter plot comparing the compounds found in the SJ samples extracted by the ACN and H_2_O (1:1, Mix 3) solvent composition against the mixture MeOH and H_2_O (1:1, Mix 4) as reference. This plot shows a “volcano” shape comparison in which a huge amount of the compounds on the left side of the *x* axis (3086) at the shaded green zone are offering both significant differences (*p*-value < 0.05) and a relative quantity of at least twofold below the chosen mixture from the D-optimal design. On the contrary, relatively few compounds found in the red zone (243) were presumably extracted to a higher extent by the presence of ACN in the example mixture.

Note that the latter evaluation was made including the different coffee types all at once, so that a new ANOVA was run on the mixture MeOH and H_2_O (1:1) comparing natural and honey vs. washed coffee, shown in Figure S6 (a and b, respectively). Although their TICs looked virtually identical, the differential analysis against the washed coffee beans shows a clear difference in the components extracted from natural, but only a few compounds different from the honey type. This is not a surprise since the pericarp must add a significantly different composition than the mere presence of the mesocarp in honey. Likewise, this also indicated that the matrix profile of the honey resembles the compounds extracted from the washed coffee type. Table 1 summarizes the number of compounds in all different extraction mixtures tested, individually and among the coffee types.

**Table 1:** Number of compounds in different extraction mixtures showing both statistical significance (*p*-value < 0.05) and at least a twofold change (|Log_2_ X_i_|> 1) in both either a higher or lower abundance of each compound (*X*_i_) versus the reference (Mix 4, MeOH/H_2_O 1:1) compared among different types of coffee from the San Juan Region (SJ)

Mix	Natural (N)		Honey (H)		Washed (V)		Among N/H/V	
	Lower	Higher	Lower	Higher	Lower	Higher	Lower	Higher
1	888	5	780	66	827	50	1605	164
2	1638	12	1416	197	1505	112	2607	262
3	1910	15	1671	164	1789	101	3086	243
5	3260	93	1684	662	1825	64	3521	195
6	2510	3	1639	106	1806	96	3429	258
7	2331	13	1605	182	1078	6	1897	38
8	1081	0	1016	10	2184	125	4490	241
9	3102	22	1871	280	2796	61	4835	163
10	3351	12	2014	289	827	50	1605	164

Briefly, it is easy to tell that the pattern is repeated in all coffee types and for all mixtures showing more compounds and only a few extracted at a higher extent compared with the reference. This result correlates well with the findings from the desirability function and the selection of “mixture 4” as the best and most effective option reflecting differences in the co-extractives as well.

### A dedicated method for ochratoxin A in raw and roasted coffee

The selected mixture 4 was evaluated for green and roasted coffee. Additionally, since in the literature there was no clear trend regarding the use of acidified solvents [38] nor this sort of cleanup method in coffee, not only the effect of the addition of formic acid, but also the use of two adsorbents for dSPE cleanup was assessed. In this case, the use of Isolute® ENV+ was selected due to the high surface area and effective retention along with the modified silicate Bondesil® PSA for cleanup in triplicate per combination adding up to 24 spiked samples compared with a corresponding level of OTA at 5 µg/kg to assess recovery. The labeling of the samples matches the class of coffee beans (G, green; T, roasted), the extraction in the first digit (0, without acid; 1, with acid), and the cleanup sorbent for dSPE (1, PSA; 2, ENV+). The batch was injected using two different acquisition methods. On one hand, the resolution of the full scan method was lowered down to 17,500 FWHM to get more scans per peak, but also to couple it with a subsequent fragmentation mediated by parameters detailed in the “Instrument conditions” section. The latter parameters were set with the aim of characterizing the extracts by triggering MS2 events to identify the compounds with differential retention. The results of a study based on the ‘Food Research’ workflow from Compound Discoverer™ are summarized in Table 2. The workflow included statistical analysis and online [56] and local database searches (Arita Lab and EFS HRAM ThermoFisher™ Scientific™) that allowed the identification of co-extracted endogenous compounds [57].

**Table 2:** Examples of endogenous compounds annotated via MS2 fragmentation at 25 NCE (FS-DDA/dd-MS2) and results of selected contrasts from the differential analysis

Compound	Rt (min)	MS1			MS2			Differential analysis		
		Predicted formula	SFit (%)	Error (ppm)	Precursor (*m/z*)	Match (%)	ID	Contrast	FC	PV
Caffeine	3.595	C_8_H_10_N_4_O_2_	12	0.39	195.1624	71.7	2	NS	< 0.5	> 0.05
Chlorogenic acid	3.048	C_16_H_18_O_9_	88	− 0.74	356.1716	99.8	2	G02/G01	2.24	0.0072
4,5-dicaffeoylquinic acid	3.860	C_25_H_24_ O_12_	85	1.28	518.1646	97.9	2	NS	< 0.5	> 0.05
1-caffeoyl-5-feruloylquinic acid	4.173	C_26_H_26_O_12_	50	1.32	531.1504	-	3	NS	< 0.5	> 0.05
3-O-feruloylquinic acid*	3.545	C_17_H_20_O_9_	89	− 0.14	370.1703	97.2	2	T02/T01	− 2.11	0.0019
Cyclo(leucylprolyl)**	3.651	C_11_H_18_N_2_O_2_	83	0.47	212.1414	98.2	2	NS	< 0.5	> 0.05
Trans-5-O-(4-coumaroyl) quinic acid	3.114	C_16_H_18_O_8_	90	0.1	339.1077	98.9	2	G02/G01	− 3.39	0.0053
								T02/T01	− 1.96	0.026
5-hydroxymethylfurfural	2.815	C_6_H_6_O_3_	45	3.16	-	-	3	None, only in T (roasted)		

*Rt* retention time, *MS1* Full Scan event at 70.000 FWHM in positive polarity, *MS2* event triggered by MS1 to select the precursor with an isolation with 3 m*/z* to undergo a higher energy collision-induced dissociation (HCD) at 25 eV and the acquisition of the fragmentation spectrum at 35.000 FWHM, *Predicted formula* result of the prediction node based on mass tolerance and elemental composition as well as the isotopic pattern matching, *SFit* score based on the spectral similarity score between the theoretical and the measured isotope pattern as a percentage, *Error* mass difference in ppm between the experimental mass and the annotated mass, *Precursor* selected precursor in (*m/z*) that triggers the MS2 event**,** *Match* best match score (0–100) from the mzCloud identity search for the compound, *ID* annotation confidence levels based on the Chemical Analysis Working Group (CAWG) of the Metabolomics Standards Initiative (MSI) [58], *NS* no evidence found above 5% significance level, *FC* log_2_ fold-change, *PV p*-value of the contrast by a multivariate *t*-test (assuming equal variance).*MS1 and MS2 matching information may correspond to the chlorogenic methylate, isobaric compound.**MS1 and MS2 matching information may correspond to the Cyclo(leucylprolyl), an isomeric compound

**Fig. 2:**
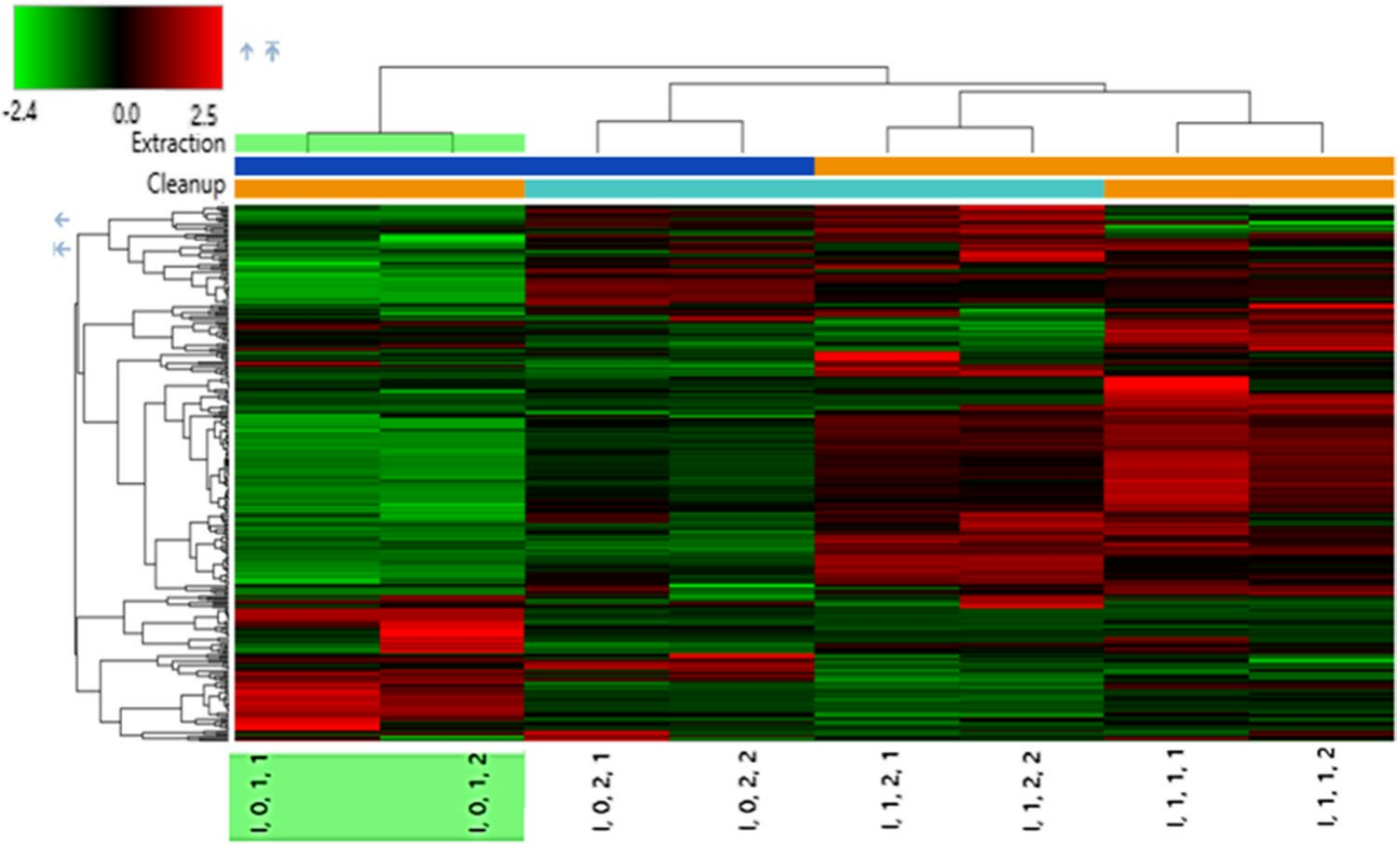
Heat map showing the correlation of the detected compounds and a roasted coffee sample (l) in the experimental design testing for extraction without acid (0 or the dark blue top bar) and with acid (1 or the yellow top bar). Likewise, the cleanup treatment factor with the adsorbent PSA (1 or the yellow top bar) and ENV+  (1 or the light blue top bar). Replicate samples are represented in the sample coding, third and last digit. The color of each rectangle represents the relative amount scaled before clustering (*z* score) according to the red/green scale located in the left-up corner. The vertical dendrogram shows dissimilar groups of compounds in relation to zones of distinct color. The horizontal dendrogram shows grouping against the single node of the first sample, difference highlighted by the green square for a better understanding

Table S3 shows examples of the experimental spectra for tentative annotation (level 3) and putative identification (level 2) [59] of polyphenols, including the feruloylquinic acid and the dicaffeoylquinic acid that have been reported in significant amounts in coffee brew [60], yet a great chemical diversity has been reported on the composition of coffee beans [44]. The occurrence of those bioactive substances and polyphenols in coffee beans that were extracted by non-acidic extraction was significantly higher for the use of ENV+ over PSA for the cleanup in the case of chlorogenic acid (green coffee) and significantly lower for feruloylquinic acid (roasted coffee) and the *trans*-5-O-(4-coumaroyl) quinic acid (in both roasted and green coffee). Regarding the 5-hydroxymethyl furfural, the relative areas did not differ significantly, neither by extraction nor the cleanup method. It is worth noticing that this toxic compound was only present in roasted coffee, as expected [61]. Similarly, the raw files were also analyzed by the differential analysis node in order to perform a principal component analysis (PCA). Figure S7 shows the cumulative variance for explaining more than 60% of variability by the principal components (PC) 1 and 2. However, the evaluation was then repeated including a larger number of roasted coffee samples including the comparison with raw extracts. As shown in Figure S8 only PC4 was able to separate groups of samples by extraction (c) and cleanup (b). At this point, a PRM acquisition was used to quantify the OTA for higher selectivity to calculate the recovery in each condition. The acidified extraction yields lower recoveries (ENV+ , 15%; PSA, 41%) than non-acidified extraction (ENV+ , 37%; PSA, 95%). According to this, the best results were obtained on the side of PSA. We also considered hierarchical cluster analysis (HCA), grouping methods by Euclidean distance, among other possibilities (Manhattan, Pearson) that gave the same result in Fig. 2. The heatmap shows duplicate samples of non-acidified and PSA cleanup in the highlighted rectangle, which corresponds to a sole condition where relatively lower intensities of most compounds could be associated with better efficiency in the cleanup stage. According to this, compounds of different natures coming from the matrix tend to stick to the PSA to a higher extent; hence, a cleaner extract is obtained. As this was also reported for OTA, yet by different conditions [62], the use of adsorbents was not taken as an option for the next step. Considering that PSA is a weak anion exchange sorbent that might also retain polar compounds via hydrogen bonding, the use of the raw extract appeared to be a reasonable way to avoid any bias when looking for more polar moieties that may form during thermal treatments such as roasting and the torrefacto process.

### Heating experiments and OTA masking during processing

**Fig. 3:**
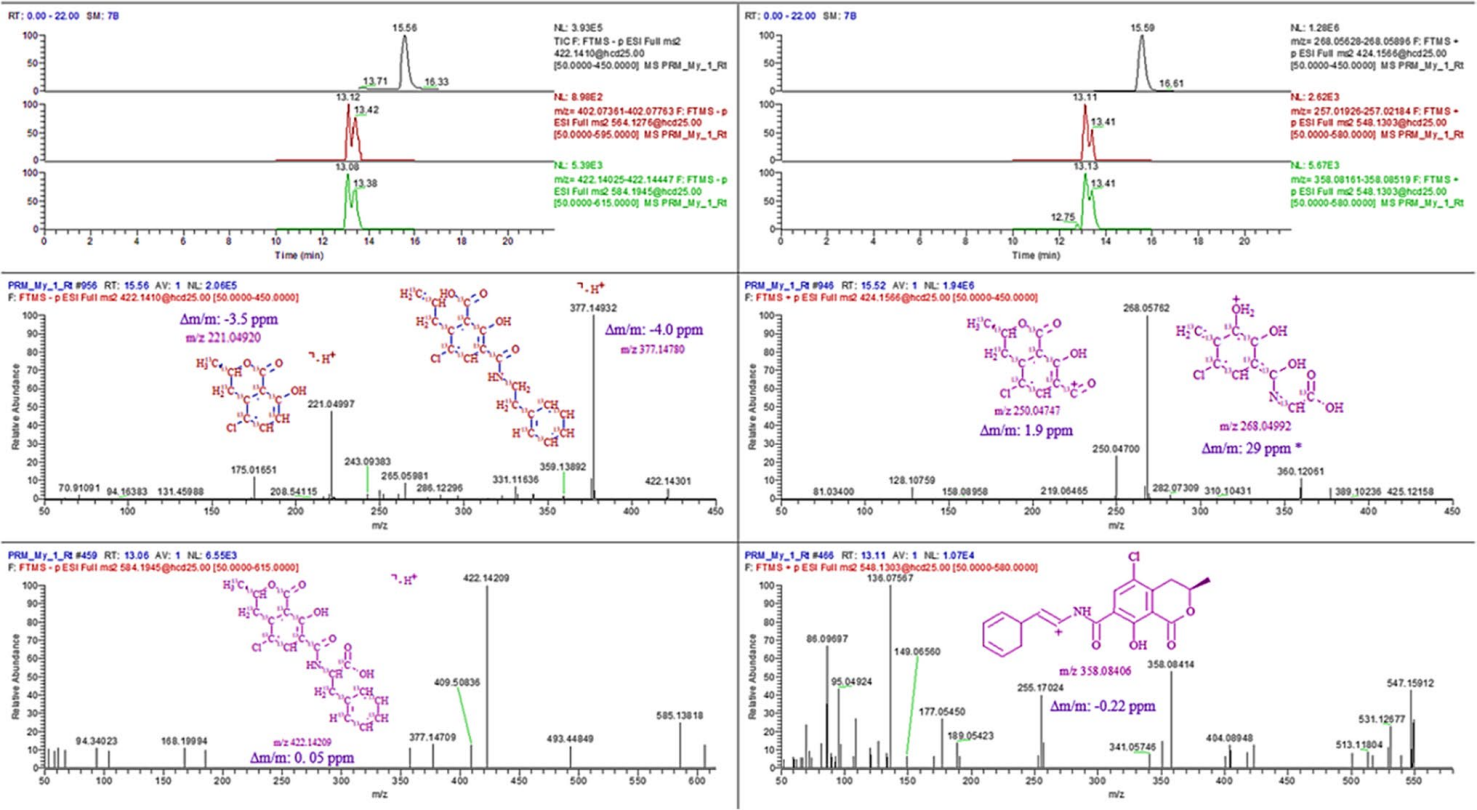
Artificially contaminated coffee with native, uniformly labeled ^13^C-OTA and added sucrose then subjected to thermal processing at 175 °C per 5 min. The material was extracted using Mix 4 MeOH/H_2_O (1:1) and directly analyzed via HRMS in PRM acquisition mode (Method 2). **a** First cell, top to bottom: TIC in black trace showing the OTA precursor in the middle cell at the retention time. Red and green negative XICs for structurally related ion products from selected precursors related with the acyl glucoside (OTA-glu) for native (*m/z* 564.1276) and labeled (*m/z* 584.1945) moieties. **b** Positive polarity, black XIC of base peak *m/z* 268.05759. Unacceptable mass error for a potential structure (*), yet the same shape and retention time is a good indicator for a true fragment related to a ^13^C-OTA moiety. Red and green XIC traces of typical fragments in OTA spectra originated from the OTA-glu precursor (*m/z* 548.1303)

The formation of saccharide esters of OTA bound to cellulose during roasting in artificially contaminated coffee beans was first studied by Bittner using methyl-α-glucopyranoside [39]. In the present study, an aliphatic compound (D (-)-sorbitol), an aromatic polyphenol (gallic acid), and a cognate compound resembling the mentioned work (D (+)-glucono-1,5-lactone) were used as model molecules in order to test the capacity of different polyhydroxylated compounds to conjugate OTA under a heat treatment process. The TracExtract analysis via isotopic pattern recognition disclosed the native and labeled OTA precursors around 14.39–14.41 min for all these trials (not shown). Nonetheless, Figure S9 shows the OTA condensation with methanol (part of the solvent mix) and its labeled feature pair separated by 20 carbons (*X*_n_ 20) found within the mass error tolerance (Δ*m*/*m* < 5 ppm) at *m/z* 438.17174. Interesting insights on the balanced conjugation with isomers showed up as part of an unbiased reaction with primary hydroxyl groups at both far ends of D (-)-sorbitol that brought in two peaks with the same spectra at 12.13 and 13.35 min. The peaks indicated native and labeled feature pairs for both positive and negative ions as shown in Figure S10 (a). The feature group of the native ^12^C-OTA and the labeled ^13^C-OTA esters correlate also with sodium adducts at *m/z* 590.13995 and 610.20654, respectively. Similarly, Figure S11 indicates that both hydroxyl groups at m- and p- positions may react with the acyl group giving the possibility to form the two gallic acid different isomers found at 16.12 and 17.01 min, both characterized by the isotopologues *m/z* 556.09869 and 576.16748. The formation of the acyl glucoside with the D (+)-glucono-1,5-lactone may take place in different positions of the cyclic compound. Arguably a preference to react with the hydroxyl position at C6 (C22 → C6’) may turn up, it notably has less steric hindrance and then might correspond to the tallest peak at 13.23 min in Figure S12. Finally, Figure S13 shows no evidence of native and labeled pairs at the expected values of the condensation product between sucrose and the OTA, yet a feature group was detected at 12.04 min in negative mode. This last pair matches the conjugation with a glucose unit (OTA-glu), a product already described [63] and presumably derived from the disaccharide decomposition during thermal exposure. Prominent ion clusters at *m/z* 564.12665 and 584.19464 are in close agreement with previous findings reflecting the formation of this acyl glucoside from cellulose, whereas no evidence of fructose binding was found here. Considering that the potential formation of OTA conjugates during the torrefacto process certainly differs from the matrix binding experiment that was performed with cellulose in the previous studies, the OTA-glu is a common factor in both cases. The sugar addition at a temperature between 150 and 200 °C at the last phase of the roasting process has been described in the literature [64]. Therefore, the next step consisted of the addition of the mix of native and labeled OTA and a sucrose solution on the bean, and the surface was left air-dried and then heated at 150 °C for 5 min. In this case, the coffee matrix was used to resemble the sugar addition taking place in the industry since the first patented industrial processes ES28829A1 and ES39494A1 [65, 66], but also allowed by the normative in force that include a sugar content up to 15% (w/w) in torrefacto products, sold only in few countries such as Spain and Argentina [67]. In light of this, the use of the acquisition Method 2 was required to handle the complexity of the coffee matrix by selecting the precursor ions already identified at the model experiment. Figure 3 shows the TIC in both polarities of the product ions of ^12^C/^13^C-OTA. The retention times slightly shifted due to the matrix composition since were correctly identified at 15.56 min and 15.59 min by their spectra in both polarities in the middle cell. Likewise, the previously identified glucoside precursors shifted, and the double peaks for native and labeled precursors laid now around 13.11 min following coaxial elution and the same peak shape. The left cell at the bottom shows the spectrum of selected labeled acyl glucoside fragments (*m/z* 564.1276) in negative mode, whereas the right-bottom spectrum shows typical fragments of the extracted traces from the native OTA (*m/z* 358.08340 and 257.02078) being part of the acyl glucoside moiety (*m/z* 548.1303) detected in positive mode. This moiety is related to the dehydration of the original OTA-glu (*m/z* 566.14236), only detected in negative mode. All the mass errors of the fragments were below 5 ppm in both polarities, except the structure proposed for the labeled ^13^C-OTA referred to the mass at *m/z* 268.05762 (28.7 ppm) which the corresponding peak fully overlaps at 15.03 min.

To study the effect of the temperature on a hypothetical OTA masking during the torrefacto process, the heating experiment was carried out at different 5 temperatures ranging from 150 to 250 °C. In short, the disaccharide solution was applied to the artificially contaminated coffee matrix, which was subjected to heating at 150, 175, 200, 225, and 250 °C for the same period in separate experiments. The heat was rapidly turned off, and the cooled samples were extracted and analyzed via LC-HRMS. Arguably the most sensitive acquisition mode in Orbitrap technology is the so-called single ion monitoring (SIM), which works in the same fashion as a Full Scan event from Method 1, but the lens and elements are set in transmission-only operating mode to avoid the loss of ions that may drop signal intensities. So, the range was narrowed down to 1 Da around selected masses to keep the C-trap filled with the least possible number of interfering ions maximizing the population of ions of interest over the AGC setting of million charges being injected into the Orbitrap. Figure S14 shows XICs from each native and labeled OTA and ion traces from native and labeled acyl glucosides in both polarities. The top fourth XICs at 15.5–15.7 min showed the dependence of the OTA signal in relation to each increase of 25 °C that led to the following behavior: a drop of 50% at 175 °C, another 60% drop at 200 °C followed by a new fall of 90% at 225 °C of OTA, that is, cumulative of only 2% of the initial amount that is completely gone at 250 °C, resembling studies described elsewhere [68]. No significant variation from 150 to 175 °C was found regarding the OTA-modified forms. However, no visible peaks of any of the glucoside traces remained beyond 200 °C. In fact, this could be explained by the sucrose decomposition at 185 °C, impairing any possibility of conjugation at higher temperatures.

### Merging single residue methods for roasted coffee

In April 2002, findings of high amounts of acrylamide in various common cooked foods were announced jointly by the University of Stockholm and the National Food Administration at a press conference in Stockholm. The determination of acrylamide remains challenging for some products ever since, especially in coffee [69]. Despite the tailing in peak shape, Figure S15 (a) shows an improvement from a split AA peak (purple dashed square), by including a large loop between the autosampler and the head of the column, an alternative to the “feed injection” mode recently included in some new models of autosamplers to correct the shape of polar pesticides. Even though the resolution was set at 70,000 FWHM, the actual resolution at the *m/z* values of the acrylamide and its isotopologues turned out to be well above 130,000 FWHM. However, the chance of missing the peak from the mass extraction window due to a slight bias calibration could lead to problems at such high resolution if the calibration frequency in a large injection sequence batch is compromised. So, the inclusion list of the acquisition method PRM (Method 3) was updated with specific transitions for AA, ^13^C-AA, and AA-d_3_ in Figure S15 (b) to have a safety net for a drop in accuracy at low *m/z* values. The use of a C18 stationary phase capable of handling 100% aqueous phase made it possible to elute the AA at 1 min without any solvent amount that ruins the peak shape. This retention time (Rt) may appear too short, but it is not that far from the Rt in official methods which is also loosely retained by a carbon-based stationary phase; it is regularly very short eluting around 4 min and shifting to 1.5 min after heavy duty. However, the Rt remains stable at 1.7 min when levels prepared in the matrix were injected (Figure S16) as a mandatory limit for Rt by ISO 16618:2015 [70]. Taking into account that most methods make use of a water-based extraction followed by SPE (e.g., Oasis MCX, Isolute® ENV+), tests using ENV+ but also PSA were performed. PSA is a weak anion exchanger, with pKa of approximately 10.1 and 10.9. The selection of the PSA sorbent has to do with the added benefit of using an aqueous-based elution that rules out the need for a MeOH evaporation step that most methods call for, usually due to a mandatory solvent exchange prior to injection. Along MeOH for ENV+, two elution buffers for PSA were tested, with the aim of assessing its effectiveness in retaining AA. Even though the ENV+ exhibits a polymeric non-polar surface hydroxylated surface that calls for MeOH for desorption, the selection of the buffers for PSA was based on the ionized amines in the surface (at pH < 8) and the induced hydrogen bonding over the very polar AA structure, which can be eventually displaced by an electrostatic competitive mechanism. Therefore, ammonium carbonate and ammonium formate at 5 mM were used to provide carboxylic moieties able to establish a charge compensation onto the PSA surface, releasing the AA into the solution. The experiments showed a differential retention among the tryouts. A simple ANOVA for a completely random design followed by Tukey’s Honestly Significant Difference (HSD) for multiple pairwise comparisons of means among the carbonate/PSA, formate/PSA, and MeOH/ENV+ systems was performed (not shown). Despite the fact that the system carbonate/PSA was able to elute a significantly lower amount of AA than the other systems tested (*p* < 0.05), no significant differences were found between the formate/PSA and MeOH/ENV+ systems (*p* = 0.5881). This led to selecting the ammonium formate as the chosen solution for elution since performs in a similar manner to the legacy system, with the added benefit of being a faster and a greener alternative to elute AA by an aqueous-based elution. In the method scheme in Fig. 1, an “in-tube” dispersive solid phase microextraction (dSPME) showed evidence of simple and non-exhaustive but effective retention of AA. This step was vortexed to assist the microextraction through the PSA-suspended particles that adsorbed AA. The study of the time required to reach the equilibrium is shown in absolute area in Figure S16 (a and b). The use of post-extraction spiked AA-d_3_ as I.S. to correct both AA (higher background) and AA-^13^C allowed the area correction vs. time (up to 20 min of vortex for adsorption) in Figure S16 (c). The value remained relatively constant through time which is easier to visualize as the same as the shortest time tested using the log_10_ of time Figure S16 (d). The results allowed the selection of 1 min as a suitable contact time during the vortex-assisted adsorption. In addition, as the amount of sorbent is limited by the size capacity of the Spin-X® filter, the amount of PSA used was also tested on (50, 100, and 150) mg, but the lowest amount was selected since no change in recovery at any of the sorbent quantities was found as shown in Figure S17. Along with the OTA, the organic phase also retains fats and oils. In fact, the EtOAc has been readily used for mycotoxins extraction [71] with the advantage of allowing the at-once physical separation of the systems as being a quite immiscible solvent, alike the others used before. In short, the method consists of a solid-liquid mini-extraction using non-miscible solvents that are then withdrawn from the remaining solids towards separate cleanup procedures, which involve an “in-tube” dSPME retention and aqueous-based elution followed by the combination with an organic dried extract (from ethyl acetate, leaving behind lipids into the inner walls). Now, instead of directly comparing our workflow against each of the official methods integrated into the scope, a general metric was calculated to give an idea of the greenness of the method by following the Analytical Greenness metric (AGREE) that includes the 12 principles of green chemistry [72] and assigning relevant weight were applicable. For instance, higher weight was used for the following principles: minimal sample size and number of samples in batch (no. 2), the selection of automated and miniaturized methods (no. 5), derivatization should be avoided (no. 6), and also that multi-analyte methods are preferred versus methods using one analyte at a time (no. 8). Lower importance was assigned for aspects that are not comparable within the kind of methods of this application such as direct techniques to avoid sample treatment (no. 1), measurements should be in situ (no. 3) which are common in environmental analytical methods, and also that renewable reagents should be preferred (no. 9) since unfortunately none of the reagents are from bio-based sources. The default weight was used for the rest of the principles and the final score added up to 0.71 (Fig. 1), which is far from the ideal maximum, but matches the efforts that we deployed to get closer to an eco-friendly initiative. In addition, no need for time-consuming methanol drying as the official method does is a plus. A single shot using a PRM acquisition Method 3 gives information only previously available by using different single residue methods, and hence, paves the way to the quantification of other contaminants as well. Thanks to the miniaturization scale, and the use of spikes with low mass amounts ILIS for AA and OTA, a suitable correction for recovery is achieved and the strong matrix effects, expected in roasted coffee samples, are corrected. Once the single residue methods were consolidated into the new method, an endurance test was performed. Figure S18 shows areas and retention time of 100 injections, and only around 50 consecutive injections the performance started to drop quickly for AA.

The representativity of the sample size was tested by the execution of multiple analyses of PT items from rounds of the FAPAS® Food Chemistry proficiency testing scheme samples of ground and instant coffee; the distribution of the calculated *z* scores showed a slight bias on the OTA results which lied mostly in the satisfactory range. Taking into account that sampling has a high impact on the variability of the analysis [73], the homogeneity assessment of the material is key although quantities < 1 g already delivered good results (e.g., 100–500 mg [74–76]). A two-stage nested design included batches (S) for between-unit homogeneity and subsamples (A-L) for within-unit homogeneity. Nonetheless, the evaluation of each batch was compared since the study for OTA apparently showed a bimodal distribution. Figure S19 (a) shows a histogram of artificially generated bootstrapped values (1000) of each batch where the confidence limits of the S1 and S3 distributions do not overlap with S2, indicating the batches may differ beyond the experimental dispersion. Likewise, the Tukey test indicated significant differences in OTA groups a and b in Figure S19 (b); however, in the individual boxplots (c), samples belong to indistinct groups regardless of the batch, except for the E and G from the same batch S2, both in the far end at the first quartile of the batch. The variation between bottles (subsamples here related with the homogeneity) is usually calculated using the expression for ${S}_{bu}^{2}$ in Table S4. For this analysis, it is assumed that data are homoscedastic, normal, random, and independent into subsamples [77]. As *S*_bu_ < 0.3 * *σ*_P_, for both AA and OTA, the material was considered adequately homogeneous. Between-unit homogeneity indicates that each 50 mg of the sample carries the same value for each property. Some materials are inherently more prone to inhomogeneity than others; however, in the case of roasted and instant coffee, a very small particle size is easy to achieve which is perhaps connected to the characteristic fragility of such material.

### Expanding the scope

**Fig. 4:**
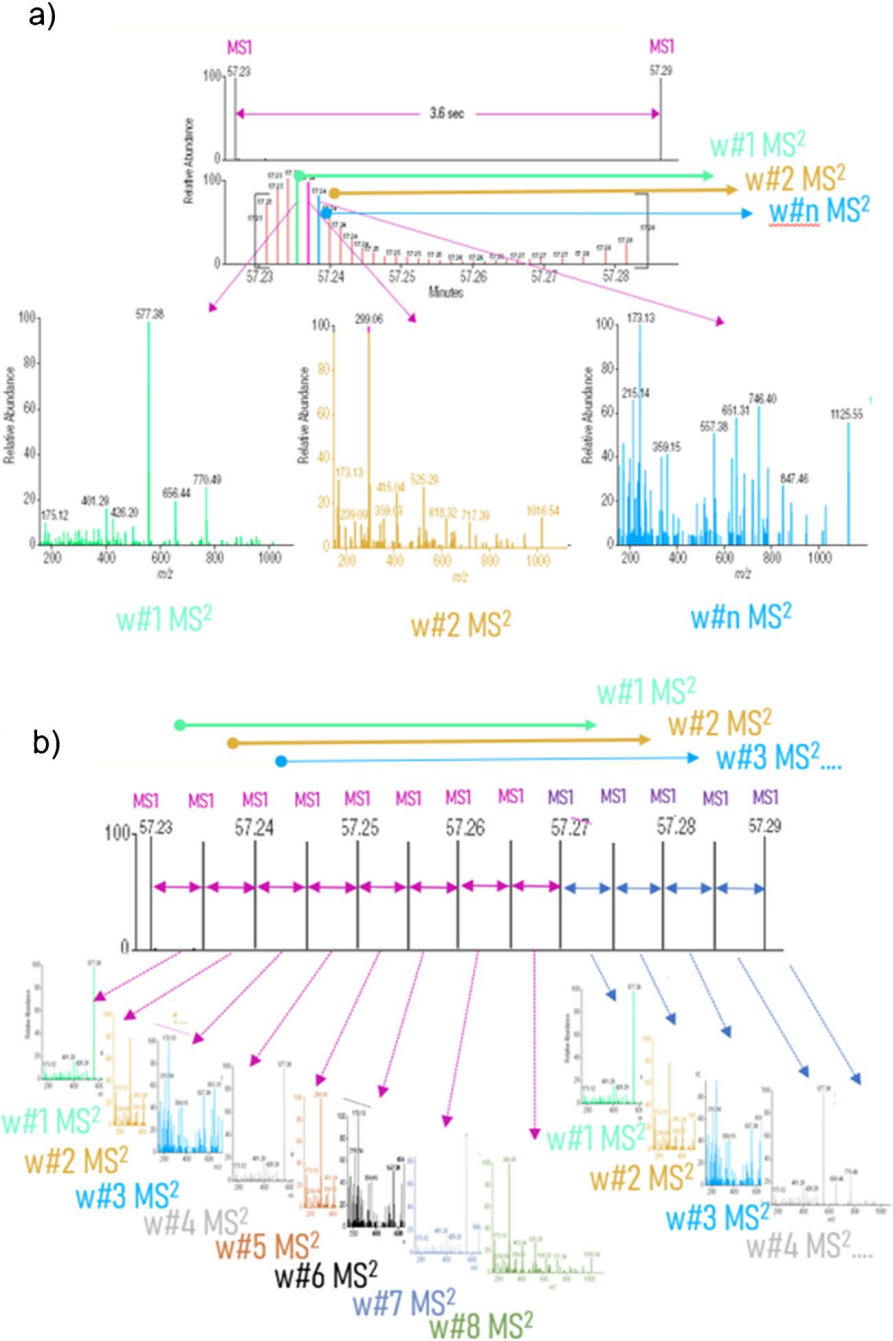
Schematic representation of the acquisition modes in LC-HRMS in Full scan (MS1) and post-fragmentation scan (MS2) of preselected segments. **a** The well-known Data Independent Acquisition (DIA) mode in combination with Full Scan creates a few segmented All-ion-fragmentation consecutive events of defined width to complete the duty cycle and only then a new MS1 acquisition is acquired again. **b** A new approach combining the Full Scan event with a counterintuitive “wide” Parallel Reaction Monitoring (FS-wPRM) that enables the acquisition of MS2 spectra with comparable selectivity (50 m*/z* width) but with the added benefit of acquiring MS1 spectra in-between. The FS-wPRM mode keeps an adequate MS2 acquisition rate for qualitative confirmation (colored top arrows) speeding up the MS1 acquisition rate improving sensitivity

Based on what is being discussed, the procedure described is a multi-purpose method for coffee that intends to get an idea of contamination in roasted coffee by just one method. On one hand, it takes advantage of the power of LC-HRMS that enables the concomitant detection of both OTA and AA with high selectivity at the required levels; however, with the aim to expand the scope further, the scheme in Fig. 1 was modified by using a slight modification as mentioned in the “Sample preparation and extraction” section, and, the acquisition method on the instrument was transformed to the FS-wPRM (Method 4) to be able to cope with a broader identification scope by the use of the full scan mode and the possibility of confirmation via MS2 events in a “friendly” way that dodge a heavy impact of the quantifier trace cycle time. Figure S20 (a) shows the Experiment Setup tab with all the important settings on the contextual right sidebar for both the Full MS and PRM boxes. Besides, a view of the inclusion list that follows the pattern of wide windows that can be shifted in time segments is shown in the floating window. Likewise, the Scatter plot in Figure S20 (b) shows the distribution of the molecular mass of the parent ion (*m/z*) and the retention time of each compound (dot and name). The red windows keep shifting through time towards higher *m/z* values in order to cover the compounds that will undergo fragmentation in a similar way that sliding windows are more selective in ion mobility [78]. However, the width of the windows was carefully selected to keep the lowest PRM events as possible in order to obtain enough data points to define the peaks for confirmation. Historically, the duty cycle in the DIA acquisition mode is severely impacted by the number of MS2 windows that delay the acquisition of a new MS1 event as shown schematically in Fig. 4a. That is not the case of the novel FS-wPRM mode, which allows acquiring the MS1 events in-between MS2 events leading to a higher acquisition rate as in Fig. 4b which therefore improves sensitivity on the quantification trace, leaving room for a maximum of 8 wide PRM windows (25 m*/z*, narrower and thereby, more selective than 50 m*/z*, the minimum width in the DIA mode).

**Fig. 5:**
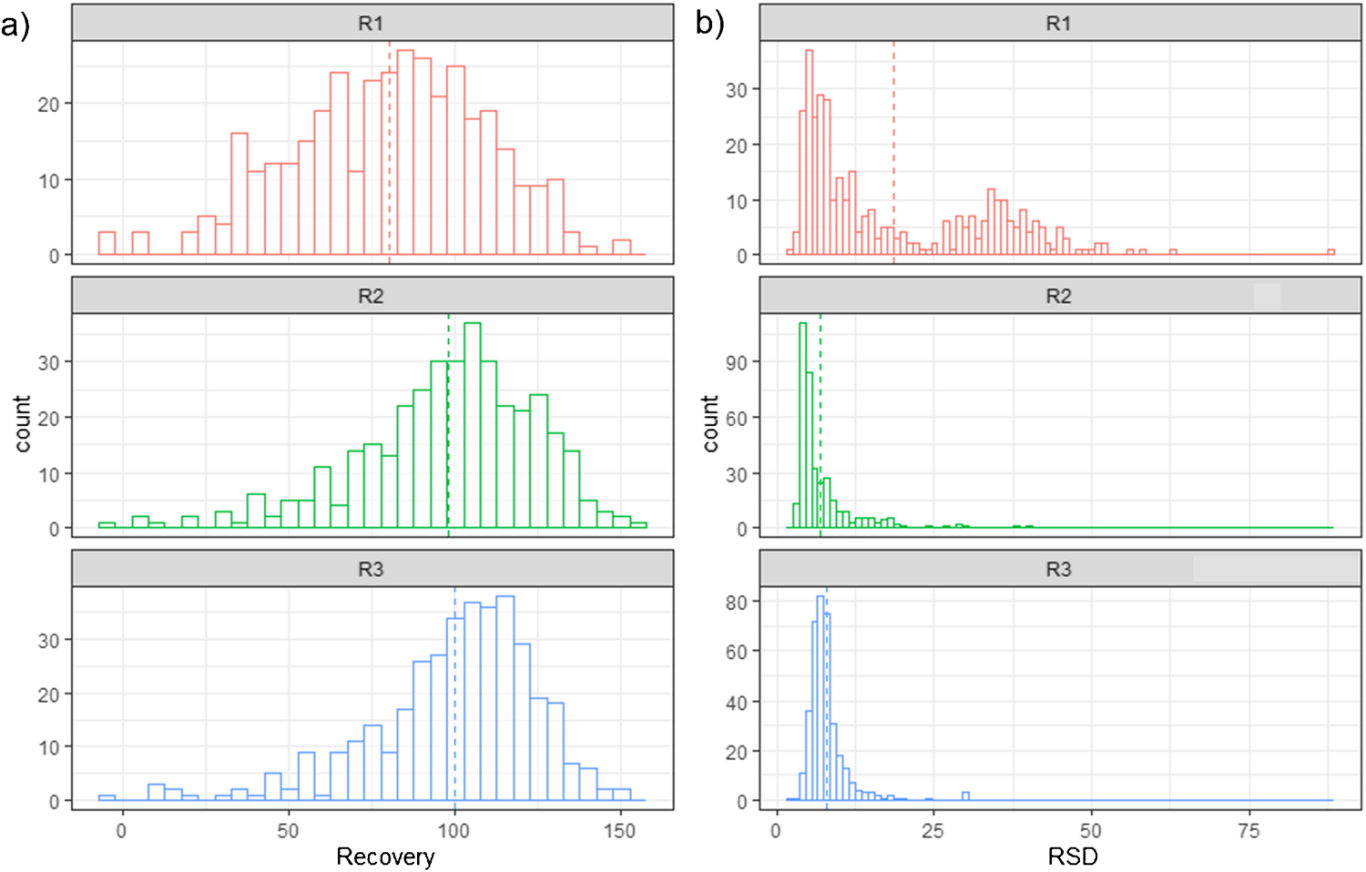
Summarized visualization of the validation results at different levels (R1, 5 µg/kg; R2, 10 µg/kg; and R3, 20 µg/kg) in roasted coffee. **a** Histogram of the recovery (*R*, %) at different levels presented individually; the dashed vertical line indicates the “global” average of the whole set at each level. **b** Histogram of the relative standard deviation (RSD, %) at different levels presented individually; the dashed vertical line indicates the “global” average of the whole set at each level. Notice that the second hump of the RSD distribution at level R1 (red) is above 25%, which was found exceeding the SANTE/11312/2021 recommendations. This in close relation with the compounds that only reached 10 µg/kg as LOQ

In addition to the perks of the FS-wPRM acquisition mode, the method has the capability to perform automatic injections of the separate AQ and OP vials. The successive in-needle combined injection proved to be unsuitable for the system since did not work well as shown in Figure S21 (a and b) as a sandwich injection. The partial combination of the phase (despite an air gap being left) led to the distortion of the AA peak shape due to the organic composition of the aliquot. Nonetheless, a split injection did make it possible to acquire the chromatogram without such distortions as shown in Figure S21 (c), allowing to get the most of the operating instrument time. Validation was carried out with the latest version of the sample preparation and acquisition method 4 by enclosing the evaluation of AA, OTA, and 512 out of c.a. 700 additional compounds used in the recovery trials, which were readily ionized by HESI-II probe operating conditions. Only 414 out of 512 assessed compounds were actually included in the validation since the rest of the compounds did not present good performance at the levels required. Identification criteria for specificity and selectivity were set for all the analyzed contaminants as confirmed (by MS2 information), but some may require verification, and collision optimization, namely, “in need to check identity.” Some were labeled as “quantified only” when MS1 information made possible the integration with a MEW < 5 ppm, but no fragmentation is available. The isotopic pattern score would also be useful for identification and gave the following values on average: 67.9% at 20 ug/kg, 60.2% at 10 ug/kg, and 47.8% at 5 ug/kg. Table S5 shows a summary of various parameters and details included in the validation plan. Calibration curves were based on a weighted regression (1/x) model based on six-point calibration curves per triplicate a day using the least squares method. The linearity was assessed by visual checking of the residual plot of response ratios (against the corresponding I.S. or I.L.I.S) versus the respective concentration levels and back-calculation differences kept acceptable ≤  ± 20%. During routine analytical sessions, an *R*^2^ > 0.990 was set as a criterion for calibration curve acceptability as in other studies that made use of the same technology in related applications [79]. Precision and trueness were assessed from repeated analyses on spiked blank coffee samples. Precision was evaluated by calculating the intermediate relative standard deviation (repeated analyses on three different days), while trueness was estimated in terms of recovery. The limit of quantification (LOQ) of the analytical method was assessed at low levels according to guidance for situations where no measurable noise is available [80]. The matrix effect was not evaluated since there was evidence of significant differences, not only in signal but in peak shapes. Matrix-matched calibration was always used for quantification and validation purposes. Atrazine-d_5_ was used as a surrogate compound at the same concentration over all the different recovery experiments which averaged 101.7% with an RSD of 17.5% (*n* = 72, 3 outliers). Since the use of the so-called typical recovery is only feasible for cognate compounds but not suitable for multiclass analytes showing different behavior, the notably dissimilar groups of compounds in the scope of the study called for a different approach. Three levels were included in the validation of the compounds, except for OTA that was also assessed below the updated regulation, despite the recovery values being low and notably differing just like the other compounds. Visualizing the results in Figure S22 (a) may give an idea of the distribution of the recoveries across the analytes at each level. In order to contrast the recoveries by level, a global comparison via an analysis of variance for a repeated measures design (RM-ANOVA) was performed in order to assess the recoveries across all the analytes, and in an independent manner as detailed in the caption. Figure S22 (b) shows all the recoveries by level and the standard error bars between and within that are not comparable with the variation across all the compounds, however, bars per compound in Figure S22 (c) let us see the variance was decomposed and the between-levels error (red bars) are higher and different from the within-level (black bars). The independent analysis fulfills the assumptions in Figure S22 (d, e) and makes it possible to compare among the groups (f) noting great significance, not only with the lowest level, but also among all the tested concentrations assessed by compound. This confirms the fact that recoveries must be reported and interpreted individually and per compound as shown in Table S5. Finally, the distribution of the recovery and the associated precision in Fig. 5 (a and b, respectively) is shown in a histogram plot per level that shows a dashed line to indicate the average value to visually note the central trend.

Even though LC–MS/MS remains the gold standard for accurate quantification of acrylamide in food [81], care must be taken regarding potential interferents in coffee leading to an overestimation of acrylamide [82]. There have been efforts to address this issue, as a bromine derivatization that has been reported [76]; however, a simpler strategy to get rid of the interferents bringing in the same ion products suggests the use of an aqueous phase without any acid [83]. Therefore, a 100% water mobile phase was set as the initial composition for the applicability exercise to analyze a set of roasted coffee samples of different origins. Despite the LC pump allowing only binary gradients, the selection valve located in the left block let us switch to the water reservoir, located in a third line (mobile phase D) that was set up in Method 4 only for the first 2 min, and then again, at the last 2 min to provide enough time to wash out the acid from the column before the next injection. Though the acrylamide quantification trace corresponds to the ion product *m/z* 55.01784, the benefits of the FS-wPRM acquisition mode allowed to also monitor the signals of certain precursors that, if present, might bring in the same product ion [84]. Figure S23 shows the method using mobile phase A and the traces of N-acetyl-β-alanine, lactamide, 3-aminopropionamide, and valine ions at *m/z* 132.06552, 90.05495, 89.07094, and 118.0563, respectively. This revealed the presence of peaks around the retention time of acrylamide and ^13^C-acrylamide for the monitored compounds, but the 3-aminopropionamide. In contrast, in the method using just water, the retention time slightly changed, shifting interferences to the left and enhancing the retention of the acrylamide towards 2.13 min. Afterwards, the method was checked by spiked blanks (*n* = 4) that clearly show the separation of a peak present in the blank before 2 min (Figure S24 a). Although acceptable recoveries of the labeled compound could help to bring to light a good method performance for this polar compound, in the case of the native analyte, the presence of interfering compounds is very telling. In fact, this presumably explains the observed overall high recoveries found in Table S5 for acrylamide, especially at the lowest concentration, in contrast to the labeled internal standard used to concurrently trace recovery that remained in close proximity to 100%. As shown in Table 3, a set of 10 samples purchased in several locations, with different processing (roasted and torrefacto) as well as variety (*robusta* and *arabica*), were analyzed to address the coelution risk up front in several scenarios. Figure S24 b displays efficacy in the separation of the left peak from the acrylamide since actually relies only on chromatography to avoid a potential misidentifying. The stacked XICs show the interferents have shifted enough (~ 1.79 min) in order to tell apart this intrusive peak that otherwise might hedge the analyte. The red trace with a notable peak apex at 2.15 min points out the native acrylamide in the recovery. The absence of signals on the flatted black trace belonging to a solvent injection indicates that the background in all the other XICs belongs to co-extractives. The second black trace of the sample M07 (Licata, Italy) was the only one that scribbled a little hump in the acrylamide retention time, way lower compared to the peak of the recovery at 400 mg/kg. Typically, high concentrations had been consistently reported on coffee samples, and those historical values were used as input to set such BML. Even though the low values in these samples may seem peculiar, highly selective methods started to report now results remarkably lower than the BML and even below 200 mg/kg [76], which is the LOQ of the current method.

**Table 3:** Commercial coffee samples (brands not disclosed) of different origins and characteristics that were used in the applicability study. Confirmed findings below the LOQ are indicated. *N.D.* not detected. The XIC trace for AA showed a S/N < 3 in all samples except for the M07 (S/N = 5.1). Certified single origin is unknown for most brands except the indicated as * Brazil and **Colombia

Sample code	Category description			Findings
	Market	Type	Variety	Mass fraction (µg/kg)
M01	Buenos Aires, Argentina	Torrefacto 100%	Blend	Chlorpyrifos (< 5)
M02	Buenos Aires, Argentina	Torrefacto 100%	Blend	N.D
M03	Madrid, Spain	Torrefacto 50%	Arabica	OTA (< 2.5)
M04	Uppsala, Sweden*	Roasted	Robusta	N.D
M05	Madrid, Spain**	Roasted	Arabica	N.D
M06	Madrid, Spain**	Torrefacto 100%	Arabica	N.D
M07	Licata, Italy	Roasted	Blend	AA (< 200)
M08	Madrid, Spain	Torrefacto 100%	Blend	OTA (< 2.5)
M09	San Juan, Tolima**	Roasted	Arabica	N.D
M10	San Antonio, Tolima**	Roasted	Arabica	N.D

Likewise, Table 3 shows that none of the samples had findings above the reporting levels for the pesticides in the scope of the method, although chlorpyrifos was found and confirmed (Fig. S25) in a sample form Argentina (M01: torrefacto, robusta/arabica blend) at a concentration in mass fraction < 5 µg/kg. Regarding OTA, Fig. S26 shows peaks confirmed by the presence of the product ion *m/z* 239.01030 in a pair of samples from Spain (M03 and M08). Nonetheless, the findings were below the lowest calibrated level and the global pooled mean concentration (3.2 µg/kg) in coffee and coffee-based products [85]. In addition, the samples were extracted with the mixture of MeOH and H_2_O (1:1, Mix 4) in a separate procedure and analyzed for the OTA-modified forms discussed before; however, no evidence of residues of the OTA-glucoside was found, neither by PRM nor by the SIM acquisition mode. No peaks around 13.12 min indicated the modified forms were not present, the absence of OTA correlates as well. However, the absence of findings in real torrefacto samples might be explained by the harsh conditions of the industrial torrefacto process in the case of any pre-existing amount of OTA that might bring up a potential conjugation. A rotating cylinder heats up the load from 200 to 240 °C [86] in industrialized roasting houses or big coffee factories, leaving little chance to detect any masked OTA left, as portrayed in the heating experiments that reach temperatures beyond the sucrose decomposition. Nonetheless, not all coffees are roasted to the same extent, the market nowadays offers a plethora of roasting flavors spread out from the Italian dark roast to the New England’s style, a light roast degree that avoids harsh conditions to keep some key nuances below the standard temperature treatment that would be a moderate scenario for the potential modifications to persist.

## Conclusions

National and global food safety surveillance systems must deal with emerging and unsuspected hazards typically by monitoring, investing, and supporting research for challenges to come. The potential presence of masked mycotoxins during the storage of semidry types of selected Colombian coffee samples was investigated, yet no evidence of OTA-modified forms was found. On the other hand, artificially contaminated coffee under heat processing revealed the formation of glucoside conjugates by using SIL techniques; however, no detected amounts were found in real samples of commercial coffee from countries where sucrose is usually added during roasting (torrefacto*)*. Worthy to note that coffee is an exceptionally complex matrix. This calls for techniques to check data, analyze, and withdraw insights during method development that help by exploring the composition of the extracts. The extraction of OTA from green coffee can be achieved with high efficiency by using a mixture of MeOH/H_2_O (1:1) and a cleanup step using PSA for dSPE. Among the advantages the miniaturization of methods may offer, the use of less amounts of solvents and waste of sorbents is environmentally friendly and convenient. In addition, this small format also enables the use of significantly reduced amounts of the very expensive isotope labeled standards used for quantification in this sort of application that currently require them for correction in routine. Through a parallel analytical strategy, the aqueous extraction of AA and the organic-based extraction of OTA (ethyl acetate) is performed in a simultaneous extraction step. This partition is also used as a key cleanup step that avoids the lipids from the aqueous phase that otherwise may hinder the “in-tube” dSPME adsorption of the acrylamide onto the PSA sorbent. The method design allowed us to expand the scope to other contaminants such as pesticides and mycotoxins that may be expected, by using a single analytical portion and the same run instead of separate procedures. In summary, the method enables the quantification of 186 compounds at 10 ug/kg, 226 at 5 ug/kg, and the acrylamide at 200 ug/kg for a total of 414 molecules, with acceptable recoveries (70–120%) and precision (RSD < 20%) making this strategy significantly faster and cost-effective. Since some compounds may undergo degradation (e.g., hydrolysis) during the organic-aqueous concurrent extraction, buffering systems that avoid interference with the dSPME adsorption may be investigated to become a way to improve recoveries of polar compounds that may exhibit instability or low sensitivity.

### Supplementary Information

Below is the link to the electronic supplementary material.

Supplementary file1 (DOCX 6358 KB)
