# Supplementary material for: A green-footprint approach for parallel multiclass analysis of contaminants in roasted coffee via LC-HRMS
Source: Anal Bioanal Chem. 2024 Feb 13;416(7):1541–60. doi: 10.1007/s00216-024-05157-4 (PMC10899293; doi:10.1007/s00216-024-05157-4)
Supplement: Supplementary file 1 — Supplementary file1 (DOCX 6358 KB) [file 216_2024_5157_MOESM1_ESM.docx]

**A green-footprint approach for parallel multiclass analysis of contaminants in roasted coffee via LC-HRMS**

Julio César España Amórtegui ^a*^, Susanne Ekroth ^b^, Heidi Pekar ^b^, and Jairo Arturo Guerrero Dallos ^a^

^a^ Chemistry Department, Science Faculty, Universidad Nacional de Colombia. Bogotá, Colombia.

^b^ Science Department, Swedish Food Agency, Uppsala, Sweden

^*^ Correspondence: jcespanaa@unal.edu.co

------------------------------------------------------

**Supplementary Information**

**Index of Tables**

Table S1. Experimental conditions D-Optimal randomized design.

Table S2. Details of the data acquisition settings in the Orbitrap analyzer depending on the type of experiment.

Table S3. Examples of endogenous compounds confirmed in coffee extracts.

Table S4. Results of the homogeneity assessment of the material using a 50 mg sample size as part of the method development

Table S5. Results on the interlaboratory validation of selected analytes in roasted coffee by using the ‘in-tube’ dSPME sample preparation method and LC-HRMS in FS-wPRM acquisition mode.

**Index of Figures**

Fig. S1 Feature pair of non-labelled/labelled 13C-U-OTA in treated coffee beans from different regions (LB, SA, SJ), types (H, N, V) and a QC of OTA at 25 ug/kg.

Fig. S2 Example of the MetExtract II metrics for the extracted ion chromatograms (EICs) of the pair non-labelled/labelled ^13^C-U-OTA

Fig. S3 Ternary model used to explore the effect between mixtures of extraction solvents ACN, MeOH, H_2_O and the OTA area from extracted ion chromatograms (EICs)

Fig. S4 Representations of the D-optimal mixtures of extraction solvents ACN, MeOH, H_2_O and the OTA area from extracted ion chromatograms (EICs)

Fig. S5 Scatter plot of the differential analysis between the samples from the San Juan (SJ) region extracted by using the mixture MeOH/H_2_O 1:1 and an alternative mixture ACN/H_2_O 1:1 as example.

Fig. S6 Scatter plot as example of the differential analysis between the samples from the San Juan (SJ) region extracted by using the mixture MeOH/H_2_O 1:1 comparing the coffee type versus washed coffee: (a) Natural coffee bean and (b) Honey coffee bean.

Fig. S7 Principal Component Analysis (PCA) results for the experimental design involving the acidification of the extraction solvent with formic acid (0: without acid, 1: with acid) and the type of sorbent (1: PSA, 2: ENV+).

Fig. S8 Scores plots of an extended experimental design applied to roasted coffee samples of several brands involving the acidification of the extraction solvent with formic acid (0: without acid, 1: with acid) and the cleanup step (0: no cleanup, 1: PSA, 2: ENV+)

Fig. S9 Feature pair of non-labelled/labelled 13C-U-OTA condensation with methanol that is part of the solvent mix 4 in the preparation for the injection vial.

Fig. S10 Evidence of the reaction of the OTA with D (-)-sorbitol as a model aliphatic compound.

Fig. S11 Evidence of the reaction of the OTA with gallic acid as a model aromatic polyphenol indicating the formation of two positional isomers.

Fig. S12 Evidence of the reaction of the OTA with D (+)-glucono-1,5-lactone as a model of a cyclic compound resembling a hexose monosaccharide.

Fig. S13 Heating experiment of the OTA with sucrose as a model disaccharide resembling the torrefacto processing.

Fig. S14 Effect of the temperature on the hypothetical OTA masking during the torrefacto process.

Fig. S15 LC-HRMS acquisition showing distinctive acrylamide peaks at 10 ng/mL in solvent.

Fig. S16 Adsorption time study of non-labelled acrylamide, acrylamide-^13^C spiked at 200 µg/kg.

Fig. S17 Assessment of the ‘in-tube’ dSPME procedure in samples spiked at 200 µg/kg.

Fig. S18 Endurance test for the method ‘in-tube’ dSPME procedure in samples over a sequence of 100 runs over the weekend, followed by a new sequence (40 runs).

Fig. S19 Homogeneity study based on a two-stage nested design with batches (S) for between-units homogeneity and subsamples (A-L) for within-units homogeneity.

Fig. S20 Schematic representation of the setup of the Full Scan - “wide” Parallel Reaction Monitoring (FS-wPRM) acquisition mode.

Fig. S21 Parallel Reaction Monitoring (PRM) in an aqueous-based sample (aQ) spiked at 400 µg/kg for acrylamide, acrylamide-d_3_ and ^13^C-acrylamide and an organic-based sample (OP) at 5 µg/kg for Ochratoxin-A using different automated injection routines.

Fig. S22. Repeated measures RM-ANOVA to test for method recovery across level concentrations for the analytes in the scope.

Fig. S23 Monitoring of endogenous compounds in coffee showing the separation from acrylamide.

Fig. S24 Separation of potential interferents from acrylamide in different samples of roasted coffee.

Fig. S25 Summary of results for a coffee sample form Argentina (M01) torrefacto, robusta/arabica blend.

Fig. S26 Summary of results for Ochratoxin A findings in coffee samples from Spain.

**Table S1.** Experimental conditions D-Optimal randomized design. Restrictions:

a) 0 ≤ ACN ≤ 0.5, b) 0 ≤ MeOH ≤ 0.5, c) 0.5 ≤ H_2_O ≤ 1, d) 0 ≤ ACN + MeOH ≤ 0.5

| Mix | Mixture component ratio | | |
| --- | --- | --- | --- |
|  | **ACN** | **MeOH** | **H_2_O** |
| 1 | 0 | 0 | 1 |
| 2 | 0 | 0.25 | 0.75 |
| 3 | 0.5 | 0 | 0.5 |
| 4 | 0 | 0.5 | 0.5 |
| 5 | 0.25 | 0.25 | 0.5 |
| 6 | 0.25 | 0 | 0.75 |
| 7 | 0.333 | 0.083 | 0.583 |
| 8 | 0.083 | 0.083 | 0.833 |
| 9 | 0.083 | 0.333 | 0.583 |
| 10 | 0.167 | 0.167 | 0.667 |

**Table S2** Details of the data acquisition settings in the Orbitrap analyzer depending on the type of experiment.

| **Method** | **Description** | **Details** |
| --- | --- | --- |
| 1 | Polarity switching - Full Scan (FS) | Resolving power (RP) of 70000 FWHM at 200 m/z, mass range from 120 to 1000 Da, Automatic Gain Control (AGC) of 3x10^6^ charges, Maximum Injection Time (maxIT) automatic, microscans: 1 and centroid spectrum data type selected. The spray voltage was 3.5 kV in positive mode, and 2.5 kV in negative mode. A single variation was implemented in the case of the *torrefacto* roasting experiment. The Single Ion Monitoring (SIM) acquisition mode was built by narrowing the mass range down to 1 Da around selected masses of interest at the retention time of OTA and the acyl glucoside product. |
| 2 | Full Scan – Data dependent MS2  (FS-ddMS2) | The conditions for the FS were the same as Method 1 experiment but only ran in positive mode and the profile spectrum data type was selected. In Data Dependent Analysis (DDA) the master scan (FS o MS1) is followed by data dependent MS2 scans that are triggered on the master scan (Discovery mode in Tune^TM^ Software). The conditions were a RP setting was 17500 FWHM, isolation window: 3.0 m/z, collision energy (CE) of 30 eV, default charge state: 1, AGC: 1x10^5^ charges, maxIT automatic, loop count: 1, minimum AGC: 1x10^3^ charges, intensity threshold set automatic, apex trigger: 3 to 9 s, dynamic exclusion in automatic, charge exclusion ≥ 2, exclusion of isotopes activated and centroid spectrum data type selected. |
| 3 | Parallel Reaction Monitoring (PRM) | RP setting was 17500 FWHM, MS2 isolation width: 3.0 m/z, CE: 25 eV, default charge state: 1, AGC: 1x10^6^ charges, maxIT automatic, and centroid spectrum data type selected. The spray voltage was 3.5 kV in positive mode. |
| 4 | Full Scan – wide Parallel Reaction Monitoring (FS-wPRM) | In the FS-wPRM acquisition mode, the previous gradient was modified to allocate more room for the separation of the whole suite of analytes. It started with 100% A. After 2 min the percentage of B was increased to 40%, followed by another increment to 70% at 16 min, and finally reached 100% at 23 min. The latter composition was additionally kept for 2 min and then steeply dropped to 0% in 0.1 min, finally this was kept for 2 min to allow column equilibration for the next run, the total runtime increased to 27 min in this extended version. The WPS-3000RS autosampler was programed with a script with instructions or UDP to take 2 µL from a fixed position containing a solution of TPP and 0.1 % formic acid as internal standard (I.S.) for the compounds without labelled counterpart available, followed by 5 µL of an air gap, and then 10 µL of aqueous-based sample to start acquisition. Afterwards, the valve switches to the inject position, sends the syringe valve to home and the needle to the parking position to wait for 30 s. Then it takes 10 µL of the organic-based sample and inject while the instrument is running. Finally, the autosampler let the valve switch to waste to wash and drain 25 µL of syringe wash solution (MeOH 50%) to get ready for the next sample. In summary, this method allows the automatic serial injection of I.S., AQ-based sample and OP-based sample for their acquisition under the same run.  The conditions for the FS were: RP 35000 FWHM, mass range from 70 to 450 Da, AGC: 3x10^6^ charges, maxIT automatic, microscans: 1 and centroid spectrum data type selected. The spray voltage was 3.5 kV in positive mode. The conditions for the PRM were the following: RP setting 35000 FWHM, isolation window: 25.0 m/z, CE: 25 eV, default charge state: 1, AGC: 1x10^6^ charges, maxIT automatic, and centroid spectrum data type selected. Wide windows were selected in the inclusion list on this novel mode (wPRM) in order to mimic a DIA acquisition mode with fragmentation at the Higher-energy Collision Dissociation (HCD) cell on selected m/z segments shifting during the run (program not shown). |

**Table S3.** Examples of endogenous compounds. MS1 spectra showing color-coded mass peaks within the tolerance in a green box and in red boxes when the isotope score is not met as criteria for a tentative structure annotation (Level 3) of the monoisotopic ion in the gray box. MS2 fragmentation spectra showing product ions fails in red and direct match on a fragment ion in green. Dots are mirrored with the library reference HCD fragmentation for a putative identification (Level 2) using the acquisition mode FS-DDA (dd-MS2).

| **Name/Structure/ID** | **MS1 (M+H + adducts)** | **MS2 (HCD, DDA vs. mzCloud library, if available)** |
| --- | --- | --- |
| Caffeine  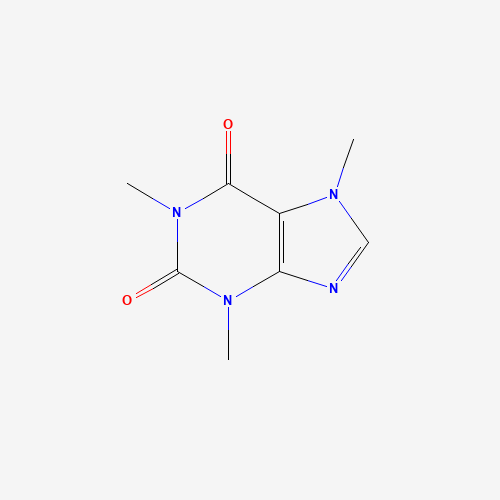  CAS 58-08-2 |  |  |
| Chlorogenic acid 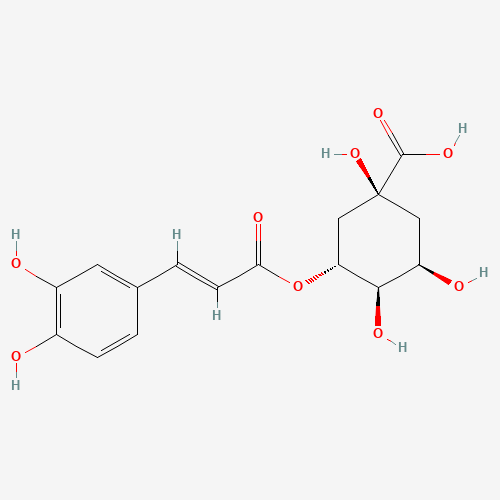  CAS 327-97-9 |  |  |
| 4,5-dicaffeoylquinic acid  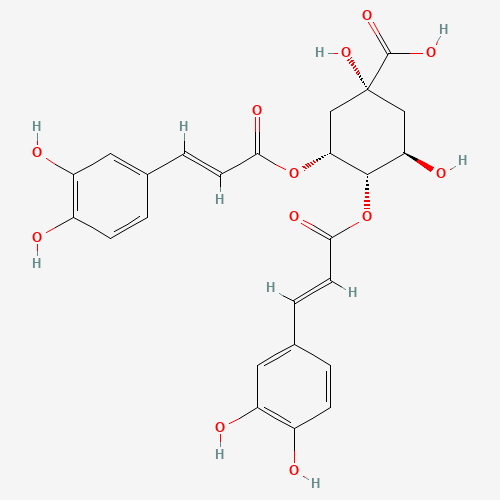  CAS 57378-72-0 |  |  |
| 1-caffeoyl-5-feruloylquinic acid  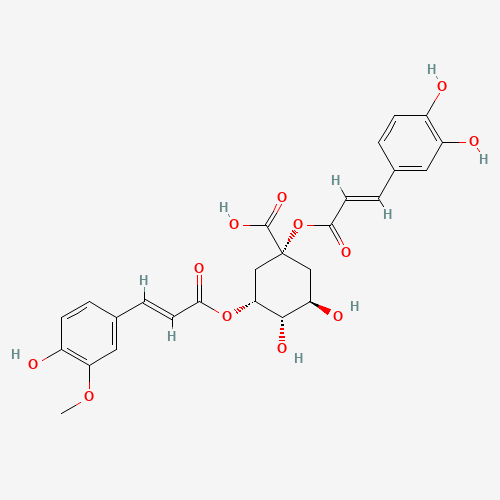  CHEBI:88343 |  | **MS2 acquired but not found at mzCloud (link to query)**    [https://www.mzcloud.org/DataViewer.aspx#/spectrumsearch#d1be8b4a-75d2-46ce-82ca-5155939405fa](https://www.mzcloud.org/DataViewer.aspx#/spectrumsearch) |
| 3-O-Feruloylquinic acid  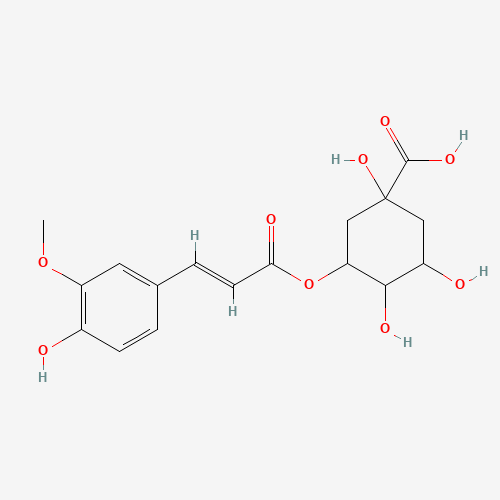  CAS 1899-29-2 |  |  |
| Cyclo(leucylprolyl)  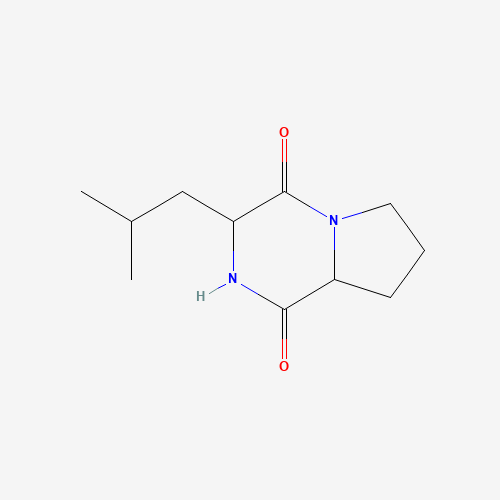  CAS 5654-86-4 |  |  |
| Trans-5-O-(4-coumaroyl) quinic acid  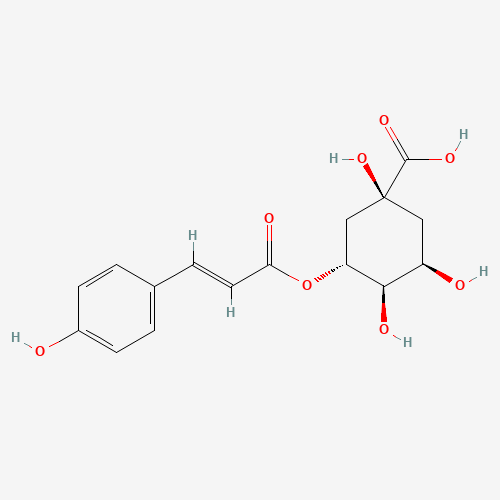  CAS 1899-30-5 |  |  |
| 5-hydroxymethylfurfural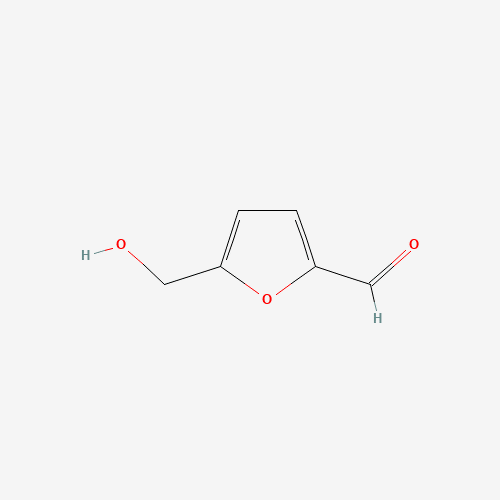  CAS 67-47-0 |  | **-MS2 not acquired – (link to library spectrum)**  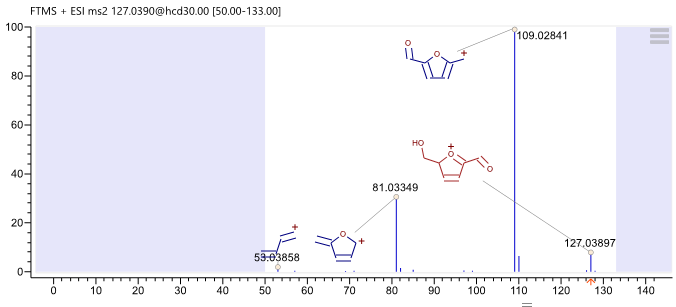  [www.mzcloud.org/dataviewer.aspx#Creference630#T1129#c#146445](http://www.mzcloud.org/dataviewer.aspx#Creference630) |

**Table S4.** Results of the homogeneity assessment of the material using a 50 mg sample size as part of the method development. The one-way ANOVA table including the between-unit (bu, batch) and within-unit (wu, subsamples) sum of squares (MS) relate to the two-stage nested design used to estimate the variance contributions.

| **Source** | **Expression** | **Component** | **AA** | **OTA** |
| --- | --- | --- | --- | --- |
| Calculations with terms from the ANOVA  Table | $S_{bu}^{2}= \frac{{MS}_{bu}-{MS}_{wu}}{n_{0}}$ | $S_{bu}$ | 16.14 | 0.0457 |
|  | $u_{bu}^{*}=\sqrt{\frac{{MS}_{wu}}{n}} x \sqrt[4]{\frac{2}{v {MS}_{wu}}}$ | $u_{bu}^{*}$ | 7.62 | 0.0235 |
|  | $S_{Meas}^{2}= \frac{{MS}_{wu}}{n}$ | S _Meas_ | 4.72 | 0.0187 |
| Calculated  uncertainty using either method | $u_{bu 1}(\%)= \frac{S_{bu}}{{Mean}_{total}}x100$ | u _bu 1_ (%) | 5.21 | 1.17 |
|  | $u_{bu 2}(\%)= \frac{u_{bu}^{*}}{{Mean}_{total}}x100$ | u _bu 2_ (%) | 2.46 | 0.60 |
| Measurement  variance | $S_{Meas}(\%)= \frac{S_{Meas}^{2}}{{Mean}_{total}}x100$ | u _Meas_ (%) | 1.52 | 0.48 |
| Standard Deviation for Proficiency | 0.3 * σ_P_ | σ_P_ (µg/kg) | 17.64† | 0.184† |

The influence of the homogeneity to the overall measurement uncertainty was calculated for either estimate (u_bu1_, u_bu2_) and then compared with the actual Standard Deviation for Proficiency (σP) to be considered adequately homogeneous for the properties ochratoxin A (OTA) and acrylamide (AA). If there are no missing data in the study planned to contain 3 observations per subsample, n_0_ (12-3 = 9) and n (35-2-9=24). Degrees of freedom (v MS_wu_). † S _bu_ ≤ 0.3 * σ_P_, the material was considered adequately homogeneous. Between-unit homogeneity indicates that each 50 mg of sample carries the same value for each property (AA and OTA). The MSbu is the variation between units and MSwu within units and the number of replicate measurements performed per unit. The calculated one-way ANOVA values showed MSbu > MSwu, this ruled out repeatability problems that would require to take actions on the method. As shown in the table, the calculations with terms from the ANOVA let to estimate the ubu, the higher value of (Sbu or u*bu) was taken as an uncertainty estimate for the homogeneity [67]. The calculation of the contribution of the homogeneity to the overall measurement uncertainty in percent was carried out and compared to the standard deviation for PT (σP) reported by the round organizer.

**Table S5.** Results on the interlaboratory validation of selected analytes in roasted coffee by using the ‘in-tube’ dSPME sample preparation method and LC-HRMS in FS-wPRM acquisition mode.

**⸹** Retention (Ret.) time with elution program using an Accucore aQ C18 length, diameter and particle size of 100 mm, 2.1 mm and 2.6 µm, respectively. Quantification (Quant.) trace matched the best found adduct or MS2 transition product. Extracted ion chromatograms are built with a mass extraction window of 5 ppm wide. Fragments by MS2 used for confirmation, however, some may require verification or collision optimization (Check ID). Few compounds without MS2, were only identified by exact mass and isotopic pattern, further orthogonal information may be necessary (Quan. only).

**⸙** Internal standard calibration using triphenylphosphate, except for acrylamide (^13^C-acrylamide) and ochratoxin A (^13^C-U-ochratoxin A). Calibration range: (2,5 – 80) ng/mL, except for acrylamide (200 – 6400) ng/mL.

† Individual recovery per assessed level. Recovery at Level N1 is marked with one star (*) for compounds exceeding RSD _Ri_ > 20 %. Compounds with unacceptable biased recovery (30 % >R>>150 %) are marked with two stars (**).

⸸ Limit of Quantification (LOQ). Relative Standard Deviation (RSD) under conditions of repeatability (RSD_r_). Between-day precision and accuracy results got from in-house validation trial, though precision is only shown at the corresponding LOQ per compound. Pooled calculation over three days under conditions of intermediate precision (RSD Ri).

‡ Due to a recent update on the regulation, the ochratoxin A was the only compound assessed at 2.5 µg/kg showing a recovery of 56.7 % and RSD _Ri_ 20.3 %. As the values are in the limit of acceptance criteria, the values at LOQ = 5 µg/kg remained in the table. Commission Regulation (EU) 2023/915 of 25 April 2023 on maximum levels for certain contaminants in food and repealing Regulation (EC) No 1881/2006 (Text with EEA relevance)

| **Compound** | **Chromatography and mass spectrometry identifiers ⸹** | | | | **Weighted Linear Regression ⸙**  **(WLS, 1/x, Levels=6, n=3)** | | | **Recovery at each level ⸶**  **(n=4, days=3)** | | | | **Precision shown**  **only at the LOQ** ⸸ | | |
| --- | --- | --- | --- | --- | --- | --- | --- | --- | --- | --- | --- | --- | --- | --- |
|  | **Formula** | **Ret. time (min)** | **Quant.**  **Trace**  **(m/z)** | **Fragments by MS2** | **Intercept** | **Slope** | **R^2^** | **N1:**  **5 µg/kg**  **(%)** | | **N2:**  **10 µg/kg (%)** | **N3:**  **20 µg/kg (%)** | **LOQ (µg/kg)** | **RSD_r_ (%)** | **RSD _Ri_ (%)** |
| 1,3-diphenylurea | C_13_H_12_N_2_O | 9.46 | 213.10224 | Confirmed | 4.82_X_10^-2^ | 2.89_X_10^-2^ | 0.9883 | 137.0 |  | 121.8 | 112.0 | 5 | 0.5 | 7.7 |
| 2-n-octyl-4-isothiazolin-3-one | C_11_H_19_NOS | 15.97 | 214.12601 | Quan only | 7.11_X_10^-2^ | 8.29_X_10^-2^ | 0.9893 | 86.1 | * | 104.6 | 106.2 | 10 | 1.9 | 3.9 |
| Acrylamide | C_3_H_5_NO | 1.90 | 55.01864 | Confirmed | 3.43_X_10^-2^ | 4.74_X_10^-4^ | 0.9972 | ** |  | 152.5 | 123.2 | 200 | 2.5 | 5.9 |
| Acrylamide-d_3_ | C_3_H_5_D_3_NO | 1.90 | 58.03744 | Confirmed | 2.56_X_10^-2^ | 6.43_X_10^-4^ | 0.9971 | 95.4 |  | 96.6 | 95.3 | 200 | 3.4 | 5.1 |
| Aflatoxin-B1 | C_17_H_12_O_6_ | 8.10 | 313.07066 | Confirmed | 3.10_X_10^-2^ | 1.63_X_10^-2^ | 0.9834 | 128.3 |  | 104.7 | 96.3 | 5 | 2.8 | 4.8 |
| Aflatoxin-G1 | C_17_H_12_O_7_ | 6.82 | 329.06558 | Confirmed | 9.12_X_10^-3^ | 1.05_X_10^-2^ | 0.9857 | 31.4 |  | 59.2 | 65.6 | 5 | 12.1 | 18.6 |
| Akton | C_12_H_14_Cl_3_O_3_PS | 20.95 | 114.96151 | Confirmed | 4.90_X_10^-3^ | 5.60_X_10^-3^ | 0.9894 | 98.8 | * | 105.5 | 99.7 | 10 | 2.0 | 6.9 |
| Aldicarb | C_7_H_14_N_2_O_2_S | 6.24 | 116.05285 | Quan only | -4.72_X_10^-3^ | 2.63_X_10^-2^ | 0.9894 | 66.6 | * | 84.6 | 89.8 | 10 | 3.3 | 5.2 |
| Allethrin | C_19_H_26_O_3_ | 20.47 | 303.19547 | Check ID | 4.08_X_10^-2^ | 3.04_X_10^-2^ | 0.9931 | 52.8 | * | 73.8 | 74.4 | 10 | 6.0 | 6.3 |
| Allidochlor | C_8_H_12_ClNO | 6.47 | 174.06802 | Quan only | -1.76_X_10^-2^ | 2.68_X_10^-2^ | 0.9929 | 61.2 |  | 55.7 | 71.1 | 5 | 10.7 | 10.2 |
| Alloxydim | C_17_H_25_NO_5_ | 15.74 | 324.18055 | Confirmed | 1.40_X_10^-2^ | 3.15_X_10^-2^ | 0.9922 | 122.2 | * | 139.6 | 139.5 | 10 | 1.5 | 4.6 |
| Ametoctradin | C_15_H_25_N_5_ | 19.05 | 276.21827 | Confirmed | 7.97_X_10^-2^ | 6.19_X_10^-2^ | 0.9927 | 114.5 | * | 125.7 | 118.1 | 10 | 2.4 | 3.9 |
| Ametryne | C_9_H_17_N_5_S | 9.95 | 228.12774 | Confirmed | 3.85_X_10^-3^ | 6.41_X_10^-2^ | 0.9875 | 82.2 |  | 100.2 | 106.3 | 5 | 4.6 | 7.1 |
| Amicarbazone | C_1_0H_19_N_5_O_2_ | 7.00 | 242.16115 | Confirmed | 6.78_X_10^-3^ | 1.05_X_10^-2^ | 0.9871 | 55.1 |  | 91.7 | 102.9 | 5 | 9.2 | 18.0 |
| Amidosulfuron | C_9_H_15_N_5_O_7_S_2_ | 8.85 | 370.04857 | Quan only | 1.70_X_10^-3^ | 7.04_X_10^-3^ | 0.9882 | 65.5 |  | 101.1 | 110.2 | 5 | 31.2 | 14.6 |
| Anilofos | C_13_H_19_ClNO_3_PS_2_ | 17.28 | 368.03053 | Confirmed | 2.59_X_10^-2^ | 2.76_X_10^-2^ | 0.9924 | 87.6 | * | 112.3 | 111.1 | 10 | 3.1 | 5.8 |
| Aspon | C_12_H_28_O_5_P_2_S_2_ | 21.58 | 379.09261 | Confirmed | 3.81_X_10^-2^ | 4.60_X_10^-2^ | 0.9921 | 98.6 | * | 112.8 | 111.7 | 10 | 5.2 | 7.1 |
| Azaconazole | C_12_H_11_Cl_2_N_3_O_2_ | 11.42 | 300.03011 | Confirmed | -2.75_X_10^-3^ | 1.20_X_10^-2^ | 0.9862 | 77.4 |  | 93.4 | 105.4 | 5 | 7.7 | 18.5 |
| Azamethiphos | C_9_H_1_0ClN_2_O_5_PS | 7.24 | 324.98093 | Confirmed | 3.92_X_10^-3^ | 1.58_X_10^-2^ | 0.9879 | 55.9 | * | 79.4 | 83.0 | 10 | 5.8 | 6.9 |
| Azimsulfuron | C_13_H_16_N_1_0O_5_S | 10.73 | 425.10986 | Confirmed | -1.55_X_10^-2^ | 6.33_X_10^-3^ | 0.9753 | 78.3 |  | 70.7 | 36.4 | 5 | 7.5 | 5.3 |
| Azinphos-methyl | C_1_0H_12_N_3_O_3_PS_2_ | 12.00 | 318.01305 | Confirmed | 4.90_X_10^-3^ | 7.34_X_10^-3^ | 0.9931 | 96.6 |  | 141.9 | 147.0 | 5 | 4.4 | 7.3 |
| Aziprotryne | C_7_H_11_N_7_S | 13.00 | 89.01711 | Confirmed | 5.01_X_10^-4^ | 2.36_X_10^-3^ | 0.9869 | 91.6 | * | 108.0 | 112.0 | 10 | 6.1 | 5.8 |
| Beflubutamid | C_18_H_17_F_4_NO_2_ | 17.01 | 356.12682 | Confirmed | 1.66_X_10^-2^ | 2.02_X_10^-2^ | 0.9913 | 93.7 |  | 113.1 | 112.9 | 5 | 3.4 | 6.4 |
| Benalaxyl | C_2_0H_23_NO_3_ | 17.24 | 326.17507 | Confirmed | 4.81_X_10^-2^ | 5.42_X_10^-2^ | 0.9930 | 115.5 | * | 130.6 | 128.4 | 10 | 2.1 | 4.8 |
| Benodanil | C_13_H_1_0INO | 9.64 | 323.98798 | Confirmed | 6.81_X_10^-3^ | 1.83_X_10^-2^ | 0.9881 | 85.1 |  | 96.3 | 98.8 | 5 | 4.8 | 10.6 |
| Benoxacor | C_11_H_11_Cl_2_NO_2_ | 11.20 | 260.02396 | Confirmed | -9.72_X_10^-3^ | 7.20_X_10^-3^ | 0.9884 | 58.2 |  | 71.9 | 82.9 | 5 | 7.3 | 4.9 |
| Bensulfuron-methyl | C_16_H_18_N_4_O_7_S | 12.03 | 411.09690 | Confirmed | 9.62_X_10^-3^ | 1.69_X_10^-2^ | 0.9866 | 118.4 |  | 133.0 | 131.4 | 5 | 12.0 | 9.1 |
| Bensulide | C_14_H_24_NO_4_PS_3_ | 16.51 | 398.06778 | Confirmed | 7.83_X_10^-3^ | 9.83_X_10^-3^ | 0.9898 | 103.5 |  | 122.5 | 120.2 | 5 | 5.6 | 4.2 |
| Benthiavalicarb-isopropyl | C_18_H_24_FN_3_O_3_S | 13.58 | 382.15952 | Quan only | 2.98_X_10^-2^ | 2.43_X_10^-2^ | 0.9849 | ** |  | 143.2 | 133.9 | 10 | 2.1 | 4.1 |
| Benzoximate | C_18_H_18_ClNO_5_ | 18.35 | 364.09463 | Confirmed | 4.59_X_10^-3^ | 8.05_X_10^-3^ | 0.9918 | 67.9 |  | 99.0 | 97.3 | 5 | 4.5 | 16.6 |
| Benzoylprop Ethyl | C_18_H_17_Cl_2_NO_3_ | 17.62 | 366.06583 | Check ID | 1.02_X_10^-2^ | 2.02_X_10^-2^ | 0.9915 | 71.6 |  | 92.7 | 91.9 | 5 | 9.0 | 15.2 |
| Benzthiazuron | C_9_H_9_N_3_OS | 8.38 | 208.05391 | Confirmed | -2.37_X_10^-3^ | 1.41_X_10^-2^ | 0.9860 | 64.6 |  | 88.4 | 90.1 | 5 | 5.2 | 12.7 |
| Bioresmethrin | C_22_H_26_O_3_ | 22.57 | 339.19547 | Confirmed | 2.76_X_10^-2^ | 2.93_X_10^-2^ | 0.9830 | 63.2 | * | 80.2 | 82.5 | 10 | 11.2 | 8.4 |
| Boscalid | C_18_H_12_Cl_2_N_2_O | 13.40 | 343.03994 | Confirmed | 4.31_X_10^-4^ | 5.45_X_10^-3^ | 0.9906 | 55.7 |  | 97.9 | 104.6 | 5 | 5.7 | 15.2 |
| Bromacil | C_9_H_13_BrN_2_O_2_ | 7.38 | 261.02332 | Confirmed | -4.45_X_10^-3^ | 2.90_X_10^-3^ | 0.9887 | 43.6 |  | 44.5 | 74.0 | 5 | 3.0 | 8.4 |
| Bromofenvinphos-ethyl | C_12_H_14_BrCl_2_O_4_P | 17.84 | 98.98434 | Confirmed | 9.78_X_10^-4^ | 1.11_X_10^-2^ | 0.9873 | 91.0 | * | 99.5 | 96.6 | 10 | 0.9 | 4.0 |
| Bromuconazole (mixture of Isomers) | C_13_H_12_BrCl_2_N_3_O | 14.50 | 375.96136 | Quan only | -3.04_X_10^-3^ | 1.63_X_10^-3^ | 0.9887 | ** |  | 40.3 | 73.3 | 10 | 13.7 | 9.9 |
| Bupirimate | C_13_H_24_N_4_O_3_S | 14.30 | 166.09714 | Confirmed | 7.53_X_10^-3^ | 7.45_X_10^-3^ | 0.9849 | 121.2 | * | 137.8 | 134.8 | 10 | 5.4 | 4.8 |
| Buprofezin | C_16_H_23_N_3_OS | 19.70 | 306.16346 | Confirmed | 2.55_X_10^-2^ | 5.27_X_10^-2^ | 0.9899 | 75.9 | * | 88.8 | 84.9 | 10 | 5.4 | 4.5 |
| Butachlor | C_17_H_26_ClNO_2_ | 19.16 | 312.17248 | Quan only | -3.82_X_10^-2^ | 4.51_X_10^-2^ | 0.9905 | 87.8 |  | 102.5 | 106.3 | 5 | 3.0 | 4.7 |
| Butylate | C_11_H_23_NOS | 19.39 | 218.15731 | Quan only | 4.67_X_10^-3^ | 2.89_X_10^-2^ | 0.9916 | 89.8 |  | 97.3 | 95.0 | 5 | 6.5 | 5.4 |
| Cadusafos | C_1_0H_23_O_2_PS_2_ | 18.63 | 271.09498 | Confirmed | 5.71_X_10^-2^ | 7.11_X_10^-2^ | 0.9922 | 99.5 |  | 108.3 | 106.4 | 5 | 6.4 | 4.8 |
| Carbaryl | C_12_H_11_NO_2_ | 8.68 | 145.06479 | Confirmed | 2.25_X_10^-2^ | 1.50_X_10^-2^ | 0.9895 | 130.0 |  | 113.3 | 104.6 | 5 | 6.8 | 6.5 |
| Carbendazim | C_9_H_9_N_3_O_2_ | 4.64 | 192.07675 | Quan only | -2.01_X_10^-2^ | 4.32_X_10^-2^ | 0.9857 | ** |  | 46.9 | 74.6 | 10 | 22.8 | 16.7 |
| Carboxine | C_12_H_13_NO_2_S | 8.44 | 236.07398 | Confirmed | 2.80_X_10^-2^ | 2.76_X_10^-2^ | 0.9876 | 95.4 | * | 100.5 | 93.0 | 10 | 6.3 | 4.5 |
| Carfentrazone-ethyl | C_15_H_14_Cl_2_F_3_N_3_O_3_ | 16.37 | 412.04371 | Confirmed | -1.96_X_10^-3^ | 8.43_X_10^-4^ | 0.9898 | 85.1 |  | 95.7 | 97.4 | 5 | 6.3 | 7.9 |
| Carpropamid | C_15_H_18_Cl_3_NO | 17.16 | 139.03059 | Confirmed | 1.76_X_10^-2^ | 1.72_X_10^-2^ | 0.9927 | 98.6 | * | 115.6 | 114.3 | 10 | 4.2 | 4.6 |
| Chlorbromuron | C_9_H_1_0BrClN_2_O_2_ | 13.41 | 292.96869 | Confirmed | -1.32_X_10^-3^ | 7.45_X_10^-3^ | 0.9917 | 57.4 |  | 91.3 | 94.8 | 5 | 15.5 | 12.8 |
| Chlorfenvinphos (mixture Isomers) | C_12_H_14_Cl_3_O_4_P | 17.43 | 358.97681 | Confirmed | 1.30_X_10^-2^ | 1.51_X_10^-2^ | 0.9925 | 84.2 |  | 101.8 | 102.0 | 5 | 5.6 | 5.2 |
| Chloridazon | C_1_0H_8_ClN_3_O | 5.47 | 222.04287 | Confirmed | -6.57_X_10^-3^ | 8.11_X_10^-3^ | 0.9892 | ** |  | 21.4 | 59.2 | 10 | 10.8 | 7.1 |
| Chlorimuron Ethyl | C_15_H_15_ClN_4_O_6_S | 13.83 | 415.04736 | Confirmed | 1.74_X_10^-3^ | 6.28_X_10^-3^ | 0.9903 | 108.1 |  | 132.6 | 133.5 | 5 | 4.9 | 8.9 |
| Chloroxuron | C_15_H_15_ClN_2_O_2_ | 15.05 | 291.08948 | Confirmed | 3.25_X_10^-2^ | 2.78_X_10^-2^ | 0.9907 | 96.3 |  | 108.4 | 110.5 | 5 | 5.9 | 6.4 |
| Chlorpyrifos | C_9_H_11_Cl_3_NO_3_PS | 20.96 | 349.93356 | Confirmed | 3.43_X_10^-3^ | 8.05_X_10^-3^ | 0.9893 | 70.5 | * | 97.2 | 94.6 | 10 | 4.4 | 7.6 |
| Chlorpyrifos-methyl | C_7_H_7_Cl_3_NO_3_PS | 18.87 | 321.90226 | Confirmed | -3.50_X_10^-3^ | 9.48_X_10^-4^ | 0.9788 | 103.6 |  | 116.3 | 108.4 | 5 | 5.7 | 9.6 |
| Chlorpyrifos-oxon | C_9_H_11_Cl_3_NO_4_P | 15.77 | 333.95640 | Confirmed | 5.99_X_10^-3^ | 1.32_X_10^-2^ | 0.9909 | 93.8 |  | 110.0 | 111.8 | 5 | 7.8 | 5.2 |
| Chromafenozide | C_24_H_3_0N_2_O_3_ | 15.02 | 395.23292 | Quan only | 1.28_X_10^-2^ | 1.55_X_10^-2^ | 0.9907 | 91.8 |  | 110.9 | 111.7 | 5 | 6.4 | 4.8 |
| Cinosulfuron | C_15_H_19_N_5_O_7_S | 7.25 | 414.10780 | Quan only | -7.87_X_10^-3^ | 9.52_X_10^-3^ | 0.9856 | 62.8 |  | 75.5 | 81.9 | 5 | 9.3 | 13.7 |
| Clethodim | C_17_H_26_ClNO_3_S | 19.42 | 360.13947 | Check ID | 1.01_X_10^-3^ | 9.75_X_10^-3^ | 0.9925 | 92.2 |  | 108.6 | 110.3 | 5 | 7.5 | 5.3 |
| Climbazol | C_15_H_17_ClN_2_O_2_ | 10.50 | 293.10513 | Confirmed | 1.32_X_10^-2^ | 3.23_X_10^-2^ | 0.9893 | 112.5 |  | 135.8 | 127.0 | 5 | 13.7 | 9.5 |
| Clodinafop-propargyl | C_17_H_13_ClFNO_4_ | 16.55 | 350.05899 | Confirmed | 1.62_X_10^-2^ | 1.92_X_10^-2^ | 0.9916 | 74.7 |  | 92.8 | 95.7 | 5 | 4.6 | 4.3 |
| Clofentezine | C_14_H_8_Cl_2_N_4_ | 19.06 | 138.01036 | Confirmed | 3.33_X_10^-3^ | 2.70_X_10^-3^ | 0.9935 | 59.3 | * | 81.6 | 88.7 | 10 | 3.9 | 3.6 |
| Clomazone | C_12_H_14_ClNO_2_ | 11.63 | 240.07858 | Confirmed | -1.99_X_10^-2^ | 3.20_X_10^-2^ | 0.9862 | 35.4 |  | 77.5 | 92.9 | 5 | 11.6 | 6.8 |
| Clomeprop | C_16_H_15_Cl_2_NO_2_ | 20.38 | 324.05526 | Quan only | 4.04_X_10^-3^ | 1.12_X_10^-2^ | 0.9902 | ** |  | 38.3 | 45.4 | 10 | 9.3 | 8.5 |
| Cloquintocet-mexyl | C_18_H_22_ClNO_3_ | 20.93 | 336.13610 | Confirmed | 5.44_X_10^-2^ | 5.66_X_10^-2^ | 0.9902 | 114.5 | * | 126.5 | 122.2 | 10 | 15.8 | 10.4 |
| Cloransulam-methyl | C_15_H_13_ClFN_5_O_5_S | 8.47 | 430.03827 | Quan only | -2.28_X_10^-3^ | 4.43_X_10^-3^ | 0.9871 | 36.9 |  | 86.1 | 97.5 | 5 | 19.0 | 10.0 |
| Coumaphos | C_14_H_16_ClO_5_PS | 17.87 | 363.02174 | Confirmed | 1.06_X_10^-2^ | 1.63_X_10^-2^ | 0.9912 | 66.5 |  | 86.8 | 87.7 | 5 | 10.5 | 11.8 |
| Coumaphos Oxon | C_14_H_16_ClO_6_P | 12.28 | 347.04458 | Confirmed | 5.12_X_10^-3^ | 1.64_X_10^-2^ | 0.9876 | 68.2 | * | 93.9 | 105.2 | 10 | 4.6 | 18.2 |
| Crimidine | C_7_H_1_0ClN_3_ | 6.38 | 172.06360 | Quan only | -6.02_X_10^-2^ | 3.78_X_10^-2^ | 0.9832 | 67.1 |  | 49.3 | 66.2 | 5 | 6.7 | 8.2 |
| Crotoxyphos | C_14_H_19_O_6_P | 13.26 | 332.12575 | Confirmed | 1.84_X_10^-2^ | 2.40_X_10^-2^ | 0.9915 | 125.0 |  | 134.3 | 128.6 | 5 | 5.7 | 4.4 |
| Cumyluron | C_17_H_19_ClN_2_O | 14.37 | 303.12587 | Confirmed | 4.28_X_10^-2^ | 2.74_X_10^-2^ | 0.9878 | 67.2 | * | 118.4 | 123.7 | 10 | 8.0 | 9.8 |
| Cyazofamid | C_13_H_13_ClN_4_O_2_S | 15.58 | 325.05205 | Confirmed | 1.12_X_10^-2^ | 1.60_X_10^-2^ | 0.9927 | 87.1 |  | 104.0 | 104.3 | 5 | 6.2 | 8.1 |
| Cybutryne | C_11_H_19_N_5_S | 13.62 | 254.14339 | Quan only | 6.14_X_10^-2^ | 7.21_X_10^-2^ | 0.9864 | 100.5 |  | 115.4 | 115.5 | 5 | 6.5 | 5.1 |
| Cyclosulfamuron | C_17_H_19_N_5_O_6_S | 15.28 | 422.11288 | Confirmed | 8.34_X_10^-3^ | 1.46_X_10^-2^ | 0.9911 | 116.8 |  | 131.8 | 135.3 | 5 | 9.1 | 11.2 |
| Cycloxidim | C_17_H_27_NO_3_S | 19.24 | 326.17844 | Check ID | 4.66_X_10^-3^ | 1.48_X_10^-2^ | 0.9881 | 93.4 |  | 112.0 | 114.2 | 5 | 9.9 | 8.5 |
| Cycluron | C_11_H_22_N_2_O | 10.86 | 199.18049 | Quan only | 6.68_X_10^-2^ | 6.24_X_10^-2^ | 0.9887 | 97.3 |  | 106.1 | 104.8 | 5 | 5.5 | 6.4 |
| Cyflufenamid | C_2_0H_17_F_5_N_2_O_2_ | 18.28 | 413.12830 | Confirmed | 1.21_X_10^-2^ | 2.07_X_10^-2^ | 0.9930 | 81.9 |  | 99.4 | 97.3 | 5 | 3.2 | 9.8 |
| Cyhalofop-butyl | C_2_0H_2_0FNO_4_ | 23.02 | 380.12686 | Quan only | -1.62_X_10^-3^ | 3.57_X_10^-3^ | 0.9912 | ** |  | 35.9 | 44.1 | 10 | 21.3 | 9.4 |
| Cymiazole | C_12_H_14_N_2_S | 5.43 | 219.09505 | Check ID | -9.78_X_10^-2^ | 3.63_X_10^-2^ | 0.9314 | ** |  | 90.0 | 80.4 | 10 | 10.1 | 10.5 |
| Cypermethrin (mix Of Isomers) | C_22_H_19_Cl_2_NO_3_ | 22.09 | 433.10802 | Check ID | -3.46_X_10^-3^ | 4.43_X_10^-3^ | 0.9899 | ** |  | 42.2 | 46.0 | 10 | 7.4 | 13.0 |
| Cyphenothrin | C_24_H_25_NO_3_ | 22.45 | 376.19072 | Check ID | -9.74_X_10^-4^ | 2.00_X_10^-3^ | 0.9810 | ** |  | 26.0 | 44.9 | 10 | 15.5 | 11.9 |
| Cyproconazole | C_15_H_18_ClN_3_O | 15.82 | 292.12112 | Confirmed | 4.42_X_10^-3^ | 1.27_X_10^-2^ | 0.9917 | 85.3 |  | 107.5 | 118.3 | 5 | 3.9 | 12.1 |
| Cyprodinil | C_14_H_15_N_3_ | 15.90 | 226.13387 | Quan only | 2.99_X_10^-2^ | 7.10_X_10^-2^ | 0.9916 | 82.4 |  | 91.1 | 92.5 | 5 | 2.2 | 6.2 |
| Cyprofuram | C_14_H_14_ClNO_3_ | 8.71 | 280.07350 | Confirmed | 1.01_X_10^-2^ | 2.28_X_10^-2^ | 0.9891 | 78.3 | * | 94.7 | 97.9 | 10 | 4.6 | 4.0 |
| Cyprosulfamide | C_18_H_18_N_2_O_5_S | 7.63 | 375.10092 | Quan only | -4.26_X_10^-3^ | 4.01_X_10^-3^ | 0.9850 | ** |  | 38.2 | 80.2 | 10 | 27.2 | 13.1 |
| Daimuron | C_17_H_2_0N_2_O | 13.68 | 269.16484 | Confirmed | 6.69_X_10^-2^ | 4.54_X_10^-2^ | 0.9834 | 121.7 |  | 137.1 | 132.2 | 5 | 7.2 | 6.0 |
| Demeton-O | C_8_H_19_O_3_PS_2_ | 11.51 | 259.05860 | Check ID | 2.24_X_10^-2^ | 1.20_X_10^-2^ | 0.9898 | ** |  | 135.8 | 115.6 | 10 | 2.4 | 4.7 |
| Demeton-S-methyl | C_6_H_15_O_3_PS_2_ | 7.87 | 231.02730 | Quan only | -8.35_X_10^-3^ | 4.26_X_10^-3^ | 0.9846 | ** |  | 36.3 | 60.7 | 10 | 8.9 | 6.5 |
| Demeton-S-Methyl-Sulfone | C_6_H_15_O_5_PS_2_ | 4.45 | 263.01713 | Quan only | 7.37_X_10^-3^ | 2.14_X_10^-2^ | 0.9894 | ** |  | 21.1 | 31.0 | 10 | 19.2 | 10.8 |
| Desmedipham | C_16_H_16_N_2_O_4_ | 11.26 | 136.03909 | Check ID | 2.70_X_10^-2^ | 2.98_X_10^-2^ | 0.9831 | ** |  | 37.3 | 37.2 | 10 | 6.7 | 6.3 |
| Desmethyl-formamido-pirimicarb | C_11_H_16_N_4_O_3_ | 7.62 | 253.12952 | Confirmed | 3.63_X_10^-2^ | 3.61_X_10^-2^ | 0.9897 | 104.2 | * | 115.3 | 110.9 | 10 | 1.9 | 3.7 |
| Desmetryn | C_8_H_15_N_5_S | 8.07 | 214.11209 | Confirmed | 3.65_X_10^-2^ | 1.13_X_10^-1^ | 0.9873 | 85.7 |  | 100.9 | 104.7 | 5 | 6.1 | 6.0 |
| Dialifos | C_14_H_17_ClNO_4_PS_2_ | 18.86 | 394.00979 | Confirmed | 1.10_X_10^-2^ | 1.19_X_10^-2^ | 0.9927 | 44.7 |  | 67.0 | 67.9 | 5 | 8.0 | 6.8 |
| Diazinon | C_12_H_21_N_2_O_3_PS | 17.22 | 305.10833 | Confirmed | 3.62_X_10^-2^ | 3.88_X_10^-2^ | 0.9902 | 105.4 |  | 125.7 | 131.5 | 5 | 5.9 | 4.4 |
| Dichlormid | C_8_H_11_Cl_2_NO | 7.57 | 208.02905 | Confirmed | -6.74_X_10^-3^ | 1.60_X_10^-2^ | 0.9924 | 62.5 |  | 85.7 | 91.6 | 5 | 7.7 | 5.4 |
| Dichlorvos | C_4_H_7_Cl_2_O_4_P | 7.51 | 220.95318 | Confirmed | -3.74_X_10^-3^ | 1.23_X_10^-2^ | 0.9925 | 73.9 |  | 105.8 | 108.9 | 5 | 11.0 | 6.3 |
| Diclobutrazol | C_15_H_19_Cl_2_N_3_O | 16.45 | 328.09779 | Confirmed | 3.02_X_10^-3^ | 1.10_X_10^-2^ | 0.9916 | 114.0 |  | 130.0 | 126.9 | 5 | 3.7 | 4.1 |
| Diclocymet | C_15_H_18_Cl_2_N_2_O | 15.79 | 172.99158 | Confirmed | -1.01_X_10^-3^ | 3.61_X_10^-3^ | 0.8908 | 98.0 | * | 111.6 | 110.9 | 10 | 7.3 | 5.4 |
| Diclosulam | C_13_H_1_0Cl_2_FN_5_O_3_S | 9.03 | 405.99382 | Quan only | -4.55_X_10^-3^ | 2.22_X_10^-3^ | 0.9911 | ** |  | 53.0 | 79.9 | 10 | 15.3 | 13.4 |
| Diethatyl-ethyl | C_16_H_22_ClNO_3_ | 16.01 | 312.13610 | Confirmed | 8.32_X_10^-3^ | 3.95_X_10^-2^ | 0.9915 | 89.2 |  | 103.5 | 109.3 | 5 | 7.3 | 6.6 |
| Diethofencarb | C_14_H_21_NO_4_ | 11.84 | 268.15433 | Confirmed | 2.87_X_10^-2^ | 2.73_X_10^-2^ | 0.9885 | 126.8 | * | 135.7 | 124.1 | 10 | 4.9 | 5.1 |
| Difenconazole (mixture of isomers) | C_19_H_17_Cl_2_N_3_O_3_ | 19.25 | 406.07197 | Confirmed | -1.47_X_10^-2^ | 9.03_X_10^-3^ | 0.9845 | 63.4 |  | 61.9 | 55.5 | 5 | 3.9 | 4.6 |
| Diflubenzuron | C_14_H_9_ClF_2_N_2_O_2_ | 16.36 | 311.03934 | Confirmed | 2.32_X_10^-3^ | 5.72_X_10^-3^ | 0.9893 | 64.9 |  | 108.3 | 108.5 | 5 | 26.8 | 12.3 |
| Diflufenican | C_19_H_11_F_5_N_2_O_2_ | 19.69 | 266.04169 | Confirmed | 6.29_X_10^-4^ | 1.10_X_10^-3^ | 0.9916 | 80.8 | * | 98.7 | 90.2 | 10 | 7.6 | 5.5 |
| Dimepiperate | C_15_H_21_NOS | 18.94 | 264.14166 | Confirmed | 1.65_X_10^-2^ | 1.69_X_10^-2^ | 0.9896 | 75.5 | * | 96.8 | 97.7 | 10 | 3.4 | 4.3 |
| Dimethachlor | C_13_H_18_ClNO_2_ | 10.76 | 256.10988 | Confirmed | -4.91_X_10^-2^ | 3.71_X_10^-2^ | 0.9816 | 77.3 |  | 85.5 | 88.3 | 5 | 4.6 | 4.3 |
| Dimethametryn | C_11_H_21_N_5_S | 14.50 | 256.15904 | Quan only | 1.51_X_10^-1^ | 1.51_X_10^-1^ | 0.9898 | 100.9 | * | 117.5 | 117.3 | 10 | 2.6 | 4.1 |
| Dimethenamid | C_12_H_18_ClNO_2_S | 12.34 | 276.08195 | Confirmed | 7.55_X_10^-3^ | 3.56_X_10^-2^ | 0.9899 | 45.5 | * | 98.2 | 108.0 | 10 | 6.9 | 17.6 |
| Dimethoate | C_5_H_12_NO_3_PS_2_ | 5.29 | 230.00690 | Confirmed | -7.09_X_10^-3^ | 1.90_X_10^-2^ | 0.9901 | ** |  | 74.0 | 81.3 | 10 | 9.0 | 11.2 |
| Dimethomorph | C_21_H_22_ClNO_4_ | 14.07 | 388.13101 | Quan only | -1.86_X_10^-3^ | 6.36_X_10^-3^ | 0.9880 | 61.5 |  | 102.7 | 116.2 | 5 | 18.3 | 14.2 |
| Dimethylvinphos | C_1_0H_1_0Cl_3_O_4_P | 14.32 | 127.01542 | Confirmed | 2.24_X_10^-2^ | 1.88_X_10^-2^ | 0.9887 | 111.5 | * | 129.2 | 128.3 | 10 | 4.1 | 4.9 |
| Dimetilan | C_1_0H_16_N_4_O_3_ | 5.55 | 241.12952 | Quan only | 3.97_X_10^-2^ | 5.43_X_10^-2^ | 0.9900 | 49.5 | * | 75.1 | 76.9 | 10 | 6.1 | 6.0 |
| Dimoxystrobin | C_19_H_22_N_2_O_3_ | 16.28 | 327.17032 | Confirmed | 5.31_X_10^-2^ | 5.26_X_10^-2^ | 0.9933 | 131.4 | * | 149.4 | 145.6 | 10 | 2.7 | 4.1 |
| Diniconazole | C_15_H_17_Cl_2_N_3_O | 18.54 | 326.08214 | Confirmed | 4.69_X_10^-3^ | 1.88_X_10^-2^ | 0.9924 | 91.3 |  | 106.0 | 104.5 | 5 | 5.1 | 4.4 |
| Dioxathion | C_12_H_26_O_6_P_2_S_4_ | 20.19 | 271.02221 | Quan only | -3.04_X_10^-2^ | 2.71_X_10^-2^ | 0.9499 | 32.2 |  | 41.6 | 42.4 | 5 | 4.9 | 12.2 |
| Diphenamid | C_16_H_17_NO | 11.03 | 240.13829 | Confirmed | 3.48_X_10^-2^ | 6.87_X_10^-2^ | 0.9894 | 115.7 | * | 126.1 | 121.9 | 10 | 3.0 | 4.0 |
| Dipropetryn | C_11_H_21_N_5_S | 14.50 | 256.15904 | Check ID | 1.51_X_10^-1^ | 1.51_X_10^-1^ | 0.9898 | 100.9 | * | 117.5 | 117.3 | 10 | 2.6 | 4.1 |
| Disulfoton Sulfone | C_8_H_19_O_4_PS_3_ | 9.59 | 307.02558 | Confirmed | 7.64_X_10^-3^ | 2.17_X_10^-2^ | 0.9887 | 96.3 | * | 112.5 | 111.5 | 10 | 3.0 | 3.4 |
| Disulfoton Sulfoxide | C_8_H_19_O_3_PS_3_ | 9.54 | 291.03067 | Confirmed | 2.50_X_10^-2^ | 3.21_X_10^-2^ | 0.9891 | 107.7 | * | 125.8 | 122.5 | 10 | 2.8 | 3.6 |
| Dithiopyr | C_15_H_16_F_5_NO_2_S_2_ | 19.53 | 402.06154 | Confirmed | -2.35_X_10^-3^ | 2.97_X_10^-3^ | 0.9895 | 43.9 |  | 94.2 | 101.5 | 5 | 22.5 | 14.8 |
| Diuron | C_9_H_1_0Cl_2_N_2_O | 11.11 | 233.02429 | Confirmed | 5.75_X_10^-3^ | 2.14_X_10^-2^ | 0.9891 | 46.4 |  | 81.8 | 88.5 | 5 | 23.5 | 11.9 |
| DMST | C_9_H_14_N_2_O_2_S | 7.86 | 215.08487 | Check ID | -6.76_X_10^-2^ | 2.27_X_10^-2^ | 0.9860 | ** |  | 43.6 | 40.9 | 10 | 12.5 | 8.6 |
| Dyfonate | C_1_0H_15_OPS_2_ | 17.11 | 247.03747 | Confirmed | -6.35_X_10^-3^ | 8.99_X_10^-3^ | 0.9857 | 94.7 |  | 121.9 | 116.1 | 5 | 9.3 | 5.2 |
| Edifenphos | C_14_H_15_O_2_PS_2_ | 17.28 | 311.03238 | Confirmed | 5.38_X_10^-2^ | 4.93_X_10^-2^ | 0.9925 | 64.9 | * | 89.3 | 92.4 | 10 | 4.9 | 5.4 |
| Epoxiconazole (iso) | C_17_H_13_ClFN_3_O | 15.82 | 330.08039 | Confirmed | 1.06_X_10^-2^ | 1.58_X_10^-2^ | 0.9910 | 111.6 |  | 119.7 | 118.4 | 5 | 10.7 | 8.8 |
| Esprocarb | C_15_H_23_NOS | 20.12 | 266.15731 | Quan only | 6.20_X_10^-2^ | 5.34_X_10^-2^ | 0.9919 | 41.6 | * | 62.5 | 66.8 | 10 | 7.6 | 6.4 |
| Etaconazole | C_14_H_15_Cl_2_N_3_O_2_ | 15.64 | 328.06141 | Confirmed | 2.10_X_10^-3^ | 1.23_X_10^-2^ | 0.9875 | 88.2 |  | 104.6 | 108.4 | 5 | 6.6 | 6.5 |
| Ethaboxam | C_14_H_16_N_4_OS_2_ | 9.09 | 321.08383 | Confirmed | -1.14_X_10^-2^ | 6.23_X_10^-3^ | 0.9849 | 51.0 | * | 50.1 | 47.8 | 10 | 23.4 | 11.7 |
| Ethametsulfuron-methyl | C_15_H_18_N_6_O_6_S | 9.58 | 411.10813 | Quan only | 1.46_X_10^-2^ | 1.27_X_10^-2^ | 0.9861 | 147.6 |  | 145.9 | 138.8 | 5 | 1.9 | 5.2 |
| Ethion | C_9_H_22_O_4_P_2_S_4_ | 20.67 | 384.99489 | Confirmed | 1.69_X_10^-2^ | 2.83_X_10^-2^ | 0.9906 | 78.4 |  | 86.5 | 87.5 | 5 | 8.4 | 6.6 |
| Ethiozin | C_9_H_16_N_4_OS | 8.09 | 229.11176 | Confirmed | -5.04_X_10^-3^ | 3.15_X_10^-2^ | 0.9890 | 34.8 | * | 69.6 | 81.7 | 10 | 6.3 | 6.1 |
| Ethiprole | C_13_H_9_Cl_2_F_3_N_4_OS | 12.61 | 396.98990 | Quan only | -3.03_X_10^-3^ | 2.73_X_10^-3^ | 0.9864 | 35.0 |  | 83.3 | 97.2 | 5 | 24.0 | 11.6 |
| Ethoxysulfuron | C_15_H_18_N_4_O_7_S | 14.23 | 399.09690 | Quan only | 7.23_X_10^-3^ | 1.04_X_10^-2^ | 0.9891 | 130.5 |  |  | 150.7 | 5 | 3.9 | 5.3 |
| Ethychlozate | C_11_H_11_ClN_2_O_2_ | 11.75 | 239.05818 | Confirmed | -2.46_X_10^-3^ | 1.59_X_10^-2^ | 0.9887 | 61.7 |  | 97.4 | 102.1 | 5 | 15.8 | 18.4 |
| Etobenzanid | C_16_H_15_Cl_2_NO_3_ | 17.70 | 340.05018 | Confirmed | 2.44_X_10^-3^ | 1.27_X_10^-2^ | 0.9908 | 66.0 |  | 85.7 | 83.6 | 5 | 8.4 | 7.4 |
| Etofenprox | C_25_H_28_O_3_ | 23.20 | 394.23767 | Confirmed | 5.51_X_10^-3^ | 3.37_X_10^-2^ | 0.9922 | 75.6 |  | 80.8 | 76.7 | 5 | 5.3 | 7.6 |
| Etoxazole | C_21_H_23_F_2_NO_2_ | 21.56 | 360.17696 | Confirmed | 1.30_X_10^-1^ | 8.41_X_10^-2^ | 0.9920 | 133.3 |  | 133.8 | 129.0 | 5 | 5.6 | 5.1 |
| Etrimfos | C_1_0H_17_N_2_O_4_PS | 16.69 | 293.07194 | Confirmed | 1.55_X_10^-2^ | 6.26_X_10^-2^ | 0.9868 | 111.8 | * | 128.7 | 125.9 | 10 | 2.4 | 3.5 |
| Famoxadone | C_22_H_18_N_2_O_4_ | 18.09 | 392.16048 | Confirmed | -4.17_X_10^-3^ | 2.36_X_10^-3^ | 0.9919 | ** |  | 36.5 | 40.2 | 10 | 10.7 | 15.5 |
| Famphur | C_1_0H_16_NO_5_PS_2_ | 10.05 | 326.02803 | Quan only | 1.07_X_10^-2^ | 2.13_X_10^-2^ | 0.9873 | 96.4 | * | 112.3 | 113.7 | 10 | 3.6 | 3.7 |
| Fenamidone | C_17_H_17_N_3_OS | 12.65 | 312.11651 | Confirmed | -1.21_X_10^-2^ | 1.77_X_10^-2^ | 0.9901 | ** |  | 86.2 | 119.3 | 10 | 4.2 | 10.5 |
| Fenamiphos | C_13_H_22_NO_3_PS | 16.04 | 304.11308 | Confirmed | 6.04_X_10^-2^ | 4.79_X_10^-2^ | 0.9917 | 99.4 |  | 111.1 | 113.1 | 5 | 3.8 | 3.9 |
| Fenamiphos-sulfone | C_13_H_22_NO_5_PS | 8.37 | 336.10291 | Quan only | 5.62_X_10^-3^ | 1.37_X_10^-2^ | 0.9828 | 75.5 |  | 91.1 | 99.2 | 5 | 7.1 | 6.2 |
| Fenamiphos-sulfoxide | C_13_H_22_NO_4_PS | 8.15 | 320.10799 | Confirmed | 2.08_X_10^-2^ | 1.94_X_10^-2^ | 0.9846 | ** |  | 53.5 | 56.7 | 10 | 10.2 | 6.2 |
| Fenarimol | C_17_H_12_Cl_2_N_2_O | 14.99 | 331.03994 | Confirmed | -1.59_X_10^-4^ | 5.81_X_10^-3^ | 0.9912 | 58.4 |  | 99.7 | 106.5 | 5 | 9.2 | 11.1 |
| Fenazaquin | C_2_0H_22_N_2_O | 23.00 | 307.18049 | Confirmed | 3.73_X_10^-2^ | 9.65_X_10^-2^ | 0.9927 | 60.6 | * | 72.5 | 69.1 | 10 | 5.5 | 4.4 |
| Fenbuconazole | C_19_H_17_ClN_4_ | 16.16 | 337.12145 | Confirmed | 3.80_X_10^-3^ | 1.10_X_10^-2^ | 0.9897 | 88.3 |  | 105.4 | 105.1 | 5 | 6.5 | 7.2 |
| Fenchlorazol-ethyl | C_12_H_8_Cl_5_N_3_O_2_ | 17.97 | 401.91319 | Confirmed | 1.21_X_10^-3^ | 6.50_X_10^-3^ | 0.9912 | ** |  | 54.0 | 57.4 | 10 | 6.9 | 14.8 |
| Fenhexamid | C_14_H_17_Cl_2_NO_2_ | 14.37 | 302.07091 | Confirmed | -1.50_X_10^-4^ | 6.40_X_10^-3^ | 0.9894 | 61.0 |  | 89.0 | 97.8 | 5 | 8.1 | 7.5 |
| Fenobucarb | C_12_H_17_NO_2_ | 11.73 | 208.13321 | Check ID | 2.53_X_10^-2^ | 4.27_X_10^-2^ | 0.9874 | 132.3 |  | 123.6 | 115.8 | 5 | 5.8 | 5.4 |
| Fenoxanil | C_15_H_18_Cl_2_N_2_O_2_ | 15.97 | 329.08181 | Confirmed | -2.07_X_10^-2^ | 1.45_X_10^-2^ | 0.9696 | 71.2 |  | 68.9 | 70.4 | 5 | 4.7 | 7.4 |
| Fenoxaprop | C_16_H_12_ClNO_5_ | 15.76 | 277.89297 | Confirmed | 1.79_X_10^-4^ | 6.37_X_10^-3^ | 0.9901 | 117.1 | * | 119.3 | 114.3 | 10 | 2.2 | 3.5 |
| Fenoxaprop-ethyl | C_18_H_16_ClNO_5_ | 19.96 | 362.07898 | Confirmed | 2.10_X_10^-2^ | 2.37_X_10^-2^ | 0.9907 | ** |  | 54.2 | 57.3 | 10 | 11.3 | 8.1 |
| Fenoxycarb | C_17_H_19_NO_4_ | 12.35 | 302.13868 | Quan only | 3.40_X_10^-2^ | 4.61_X_10^-2^ | 0.9927 | 128.6 |  | 133.0 | 132.8 | 5 | 10.5 | 6.8 |
| Fenpiclonil | C_11_H_6_Cl_2_N_2_ | 12.12 | 236.99808 | Confirmed | -3.25_X_10^-3^ | 1.52_X_10^-3^ | 0.9851 | ** |  | 42.2 | 73.0 | 10 | 11.3 | 14.7 |
| Fenpropidin | C_19_H_31_N | 11.66 | 274.25293 | Confirmed | 1.96_X_10^-2^ | 6.62_X_10^-2^ | 0.9885 | 75.6 | * | 82.6 | 75.6 | 10 | 4.5 | 4.4 |
| Fenpropimorph | C_2_0H_33_NO | 12.03 | 304.26349 | Confirmed | 1.68_X_10^-2^ | 4.80_X_10^-2^ | 0.9871 | 85.2 | * | 97.0 | 91.1 | 10 | 4.3 | 4.9 |
| Fenpyroximate | C_24_H_27_N_3_O_4_ | 22.05 | 422.20743 | Check ID | 1.70_X_10^-2^ | 1.97_X_10^-2^ | 0.9857 | 89.1 |  | 95.1 | 96.6 | 5 | 6.3 | 4.5 |
| Fensulfothion | C_11_H_17_O_4_PS_2_ | 10.78 | 309.03786 | Confirmed | 3.01_X_10^-2^ | 2.92_X_10^-2^ | 0.9885 | 100.5 | * | 118.0 | 117.3 | 10 | 4.0 | 4.1 |
| Fensulfothion-oxon | C_11_H_17_O_5_PS | 6.38 | 293.06071 | Check ID | 6.09_X_10^-3^ | 1.98_X_10^-2^ | 0.9847 | ** |  | 28.9 | 37.0 | 10 | 4.7 | 4.7 |
| Fensulfothion-oxon-sulfone | C_11_H_17_O_6_PS | 6.47 | 309.05562 | Confirmed | 2.08_X_10^-3^ | 1.06_X_10^-2^ | 0.9892 | 56.9 | * | 81.5 | 89.2 | 10 | 6.5 | 5.5 |
| Fensulfothion-sulfone | C_11_H_17_O_5_PS_2_ | 11.00 | 325.03278 | Check ID | 3.62_X_10^-3^ | 1.24_X_10^-2^ | 0.9901 | 79.9 |  | 99.8 | 101.8 | 5 | 7.8 | 9.6 |
| Fenthion | C_1_0H_15_O_3_PS_2_ | 17.28 | 279.02730 | Confirmed | -3.03_X_10^-3^ | 3.84_X_10^-3^ | 0.9903 | ** |  | 79.6 | 101.9 | 10 | 6.7 | 8.6 |
| Fenthion Oxon | C_1_0H_15_O_4_PS | 11.95 | 263.05014 | Confirmed | 3.13_X_10^-2^ | 4.56_X_10^-2^ | 0.9894 | 94.5 | * | 111.2 | 111.4 | 10 | 2.3 | 3.4 |
| Fenthion Sulfoxide | C_1_0H_15_O_4_PS_2_ | 8.78 | 295.02221 | Confirmed | 1.45_X_10^-2^ | 2.31_X_10^-2^ | 0.9880 | 86.4 | * | 103.2 | 102.9 | 10 | 1.4 | 4.5 |
| Fenthion-ethyl | C_12_H_19_O_3_PS_2_ | 19.92 | 307.05860 | Quan only | 1.19_X_10^-2^ | 1.61_X_10^-2^ | 0.9931 | 34.5 |  | 67.5 | 71.0 | 5 | 14.6 | 16.8 |
| Fenthion-sulfone | C_1_0H_15_O_5_PS_2_ | 9.08 | 311.01713 | Confirmed | -5.80_X_10^-4^ | 6.79_X_10^-3^ | 0.9881 | 50.6 |  | 88.3 | 96.4 | 5 | 15.9 | 13.5 |
| Fenthion-sulfoxide | C_1_0H_15_O_4_PS_2_ | 8.78 | 295.02221 | Confirmed | 1.45_X_10^-2^ | 2.31_X_10^-2^ | 0.9880 | 86.4 | * | 103.2 | 102.9 | 10 | 1.4 | 4.5 |
| Ferimzone 1 | C_15_H_18_N_4_ | 9.50 | 255.16042 | Confirmed | 5.00_X_10^-2^ | 3.22_X_10^-2^ | 0.9901 | 122.1 |  | 148.5 | 139.7 | 5 | 9.5 | 15.6 |
| Ferimzone 2 | C_15_H_18_N_4_ | 10.85 | 255.16042 | Confirmed | 6.30_X_10^-2^ | 4.68_X_10^-2^ | 0.9897 | 123.1 |  | 130.1 | 114.9 | 5 | 5.5 | 7.8 |
| Flamprop Isopropyl | C_19_H_19_ClFNO_3_ | 17.35 | 105.03368 | Confirmed | 4.66_X_10^-2^ | 7.58_X_10^-2^ | 0.9918 | 86.5 | * | 99.8 | 98.4 | 10 | 3.6 | 4.4 |
| Flamprop-methyl (metavane) | C_17_H_15_ClFNO_3_ | 14.14 | 105.03367 | Confirmed | 2.70_X_10^-2^ | 2.85_X_10^-2^ | 0.9887 | 116.6 | * | 126.1 | 122.6 | 10 | 2.6 | 5.1 |
| Flamprop-M-isopropyl | C_19_H_19_ClFNO_3_ | 17.35 | 105.03368 | Confirmed | 4.66_X_10^-2^ | 7.58_X_10^-2^ | 0.9918 | 86.5 | * | 99.8 | 98.4 | 10 | 3.6 | 4.4 |
| Flazasulfuron | C_13_H_12_F_3_N_5_O_5_S | 11.73 | 182.05583 | Confirmed | 5.53_X_10^-3^ | 1.05_X_10^-2^ | 0.9856 | 126.4 | * | 134.4 | 129.1 | 10 | 3.2 | 3.6 |
| Florasulam | C_12_H_8_F_3_N_5_O_3_S | 6.07 | 360.03727 | Quan only | -5.25_X_10^-3^ | 3.31_X_10^-3^ | 0.9922 | 47.7 | * | 62.2 | 89.1 | 10 | 13.9 | 13.2 |
| Fluacrypyrim | C_2_0H_21_F_3_N_2_O_5_ | 18.92 | 427.14753 | Quan only | 1.57_X_10^-2^ | 2.56_X_10^-2^ | 0.9898 | 112.9 | * | 131.9 | 124.4 | 10 | 3.5 | 4.5 |
| Fluazifop | C_15_H_12_F_3_NO_4_ | 18.54 | 328.07912 | Confirmed | 3.00_X_10^-3^ | 1.18_X_10^-2^ | 0.9924 | 74.7 | * | 95.5 | 101.4 | 10 | 7.0 | 8.8 |
| Fluazifop-Butyl | C_19_H_2_0F_3_NO_4_ | 20.10 | 384.14172 | Confirmed | 7.03_X_10^-2^ | 6.53_X_10^-2^ | 0.9937 | 55.8 | * | 72.8 | 74.5 | 10 | 5.9 | 5.6 |
| Flufenacet | C_14_H_13_F_4_N_3_O_2_S | 14.84 | 364.07374 | Confirmed | 2.59_X_10^-2^ | 2.84_X_10^-2^ | 0.9900 | 101.9 |  | 113.3 | 116.5 | 5 | 5.4 | 7.7 |
| Flumetsulam | C_12_H_9_F_2_N_5_O_2_S | 5.07 | 326.05178 | Quan only | -9.36_X_10^-3^ | 5.46_X_10^-3^ | 0.9868 | ** |  | 42.2 | 61.3 | 10 | 13.1 | 14.8 |
| Flumorph | C_21_H_22_FNO_4_ | 11.49 | 285.09125 | Confirmed | 7.49_X_10^-3^ | 1.20_X_10^-2^ | 0.9859 | 101.7 | * | 120.0 | 115.7 | 10 | 0.9 | 4.2 |
| Fluometuron | C_1_0H_11_F_3_N_2_O | 9.22 | 233.08962 | Confirmed | 1.45_X_10^-2^ | 3.58_X_10^-2^ | 0.9902 | ** |  | 110.5 | 104.4 | 10 | 7.7 | 5.2 |
| Fluopicolide | C_14_H_8_Cl_3_F_3_N_2_O | 13.36 | 382.97271 | Quan only | 4.03_X_10^-4^ | 9.28_X_10^-3^ | 0.9906 | 77.6 |  | 100.0 | 103.9 | 5 | 8.8 | 11.6 |
| Fluopyram | C_16_H_11_ClF_6_N_2_O | 14.53 | 397.05369 | Quan only | 1.52_X_10^-2^ | 2.15_X_10^-2^ | 0.9906 | 100.1 |  | 113.9 | 114.7 | 5 | 6.5 | 6.4 |
| Fluquinconazole | C_16_H_8_Cl_2_FN_5_O | 14.75 | 376.01627 | Quan only | -3.91_X_10^-3^ | 2.05_X_10^-3^ | 0.9875 | ** |  | 57.0 | 83.6 | 10 | 14.7 | 14.7 |
| Fluridone | C_19_H_14_F_3_NO | 12.22 | 330.11003 | Confirmed | 3.27_X_10^-2^ | 4.40_X_10^-2^ | 0.9882 | 129.8 | * | 144.4 | 142.3 | 10 | 0.8 | 4.1 |
| Fluroxypyr | C_7_H_5_Cl_2_FN_2_O_3_ | 21.38 | 254.97340 | Quan only | 4.56_X_10^-4^ | 2.62_X_10^-3^ | 0.9902 | ** |  | 39.7 | 61.2 | 10 | 21.8 | 13.8 |
| Flurprimidol | C_15_H_15_F_3_N_2_O_2_ | 13.65 | 313.11584 | Confirmed | -1.58_X_10^-2^ | 1.26_X_10^-2^ | 0.9909 | 63.1 |  | 66.5 | 108.9 | 5 | 6.6 | 12.9 |
| Flurtamone | C_18_H_14_F_3_NO_2_ | 12.83 | 334.10494 | Confirmed | 4.89_X_10^-2^ | 3.49_X_10^-2^ | 0.9879 | 143.7 | * | 146.5 | 137.5 | 10 | 1.5 | 4.1 |
| Flusilazole | C_16_H_15_F_2_N_3_Si | 16.50 | 316.10761 | Confirmed | -3.74_X_10^-3^ | 1.75_X_10^-2^ | 0.9901 | 39.4 |  | 92.7 | 105.0 | 5 | 23.2 | 16.2 |
| Fluthiacet-methyl | C_15_H_15_ClFN_3_O_3_S_2_ | 17.09 | 404.03002 | Confirmed | 6.13_X_10^-3^ | 1.24_X_10^-2^ | 0.9896 | 76.8 |  | 103.3 | 100.0 | 5 | 10.2 | 7.0 |
| Flutolanil | C_17_H_16_F_3_NO_2_ | 13.51 | 324.12059 | Confirmed | 3.92_X_10^-2^ | 3.06_X_10^-2^ | 0.9874 | 131.5 | * | 135.9 | 123.7 | 10 | 5.9 | 4.6 |
| Flutriafol | C_16_H_13_F_2_N_3_O | 9.91 | 302.10995 | Confirmed | -2.04_X_10^-2^ | 1.33_X_10^-2^ | 0.9870 | 46.4 | * | 62.9 | 114.2 | 10 | 12.1 | 17.8 |
| Forchlorfenuron | C_12_H_1_0ClN_3_O | 10.96 | 248.05852 | Confirmed | 2.15_X_10^-3^ | 8.81_X_10^-3^ | 0.9869 | 78.2 |  | 104.7 | 108.3 | 5 | 5.9 | 9.8 |
| Fosthiazate | C_9_H_18_NO_3_PS_2_ | 9.04 | 284.05385 | Confirmed | 2.89_X_10^-2^ | 4.20_X_10^-2^ | 0.9905 | 117.0 | * | 128.0 | 125.2 | 10 | 2.0 | 4.3 |
| Furalaxyl | C_17_H_19_NO_4_ | 12.35 | 302.13868 | Confirmed | 3.40_X_10^-2^ | 4.61_X_10^-2^ | 0.9927 | 128.6 |  | 133.0 | 132.8 | 5 | 10.5 | 6.8 |
| Furathiocarb | C_18_H_26_N_2_O_5_S | 20.00 | 383.16352 | Confirmed | 2.87_X_10^-2^ | 3.64_X_10^-2^ | 0.9914 | 71.7 | * | 91.6 | 87.4 | 10 | 5.2 | 7.1 |
| Furmecyclox | C_14_H_21_NO_3_ | 16.72 | 252.15942 | Confirmed | 3.72_X_10^-2^ | 5.91_X_10^-2^ | 0.9889 | 107.9 | * | 128.2 | 128.8 | 10 | 5.7 | 4.7 |
| Halosulfuron-methyl | C_13_H_15_ClN_6_O_7_S | 14.83 | 435.04842 | Quan only | 1.58_X_10^-3^ | 3.95_X_10^-3^ | 0.9897 | 47.9 |  | 109.8 | 121.3 | 5 | 18.3 | 8.9 |
| Haloxyfop | C_15_H_11_ClF_3_NO_4_ | 16.11 | 362.04015 | Confirmed | -1.45_X_10^-3^ | 3.63_X_10^-3^ | 0.9895 | 65.2 |  | 127.8 | 132.1 | 5 | 15.1 | 10.9 |
| Haloxyfop-2-Ethoxyethyl | C_19_H_19_ClF_3_NO_5_ | 19.86 | 434.09766 | Confirmed | 1.08_X_10^-2^ | 1.88_X_10^-2^ | 0.9871 | 63.8 |  | 76.7 | 76.8 | 5 | 10.9 | 8.6 |
| Haloxyfop-methyl | C_16_H_13_ClF_3_NO_4_ | 18.83 | 376.05580 | Confirmed | 5.15_X_10^-2^ | 4.54_X_10^-2^ | 0.9927 | 85.5 | * | 108.9 | 107.5 | 10 | 4.9 | 4.3 |
| Heptenophos | C_9_H_12_ClO_4_P | 10.79 | 251.02345 | Confirmed | 5.26_X_10^-3^ | 1.68_X_10^-2^ | 0.9861 | 83.1 |  | 102.5 | 107.7 | 5 | 3.8 | 3.8 |
| Hexaconazole | C_14_H_17_Cl_2_N_3_O | 17.61 | 314.08214 | Confirmed | 2.36_X_10^-3^ | 1.04_X_10^-2^ | 0.9914 | 86.3 |  | 109.0 | 110.3 | 5 | 8.9 | 6.1 |
| Hexazinon | C_12_H_2_0N_4_O_2_ | 8.28 | 253.16590 | Confirmed | 1.66_X_10^-2^ | 4.15_X_10^-2^ | 0.9881 | 46.2 | * | 70.8 | 74.2 | 10 | 5.2 | 4.0 |
| Hexythiazox | C_17_H_21_ClN_2_O_2_S | 20.98 | 353.10850 | Confirmed | -2.87_X_10^-3^ | 6.74_X_10^-3^ | 0.9874 | 36.2 |  | 59.8 | 62.1 | 5 | 20.5 | 10.1 |
| Imazalil | C_14_H_14_Cl_2_N_2_O | 9.52 | 297.05560 | Confirmed | 2.75_X_10^-2^ | 2.52_X_10^-2^ | 0.9883 | 108.2 |  | 123.2 | 116.6 | 5 | 4.6 | 3.7 |
| Imazethapyr | C_15_H_19_N_3_O_3_ | 6.65 | 290.14992 | Confirmed | -4.61_X_10^-2^ | 2.35_X_10^-2^ | 0.9889 | 46.8 |  | 27.8 | 21.8 | 5 | 2.2 | 3.7 |
| Imazosulfuron | C_14_H_13_ClN_6_O_5_S | 14.22 | 413.04294 | Confirmed | -4.63_X_10^-4^ | 4.28_X_10^-3^ | 0.9879 | 49.4 |  | 103.4 | 113.4 | 5 | 6.7 | 9.8 |
| Imibenconazole | C_17_H_13_Cl_3_N_4_S | 20.78 | 410.99993 | Confirmed | -2.24_X_10^-4^ | 3.47_X_10^-3^ | 0.9890 | 41.1 |  | 76.9 | 84.6 | 5 | 31.5 | 14.3 |
| Imiprothrin | C_17_H_22_N_2_O_4_ | 14.53 | 319.16523 | Check ID | 5.27_X_10^-3^ | 9.19_X_10^-3^ | 0.9906 | 74.2 |  | 103.1 | 107.0 | 5 | 10.6 | 5.1 |
| Inabenfide | C_19_H_15_ClN_2_O_2_ | 12.81 | 339.08948 | Check ID | -8.93_X_10^-3^ | 5.39_X_10^-3^ | 0.9915 | ** |  | 68.4 | 83.7 | 10 | 10.1 | 14.8 |
| Indanofan | C_2_0H_17_ClO_3_ | 15.17 | 341.09390 | Check ID | -3.88_X_10^-3^ | 1.88_X_10^-3^ | 0.9890 | ** |  | 33.5 | 64.2 | 10 | 20.8 | 9.4 |
| Indaziflam | C_16_H_2_0FN_5_ | 14.81 | 302.17755 | Confirmed | 5.63_X_10^-2^ | 4.00_X_10^-2^ | 0.9882 | 108.3 |  | 119.8 | 118.3 | 5 | 8.4 | 5.5 |
| Ipconazole | C_18_H_24_ClN_3_O | 19.46 | 334.16807 | Confirmed | 8.99_X_10^-3^ | 1.68_X_10^-2^ | 0.9924 | 78.4 |  | 94.2 | 91.1 | 5 | 3.6 | 14.9 |
| Iprovalicarb | C_18_H_28_N_2_O_3_ | 14.34 | 321.21727 | Check ID | -3.99_X_10^-2^ | 3.74_X_10^-2^ | 0.9848 | 91.3 |  | 92.2 | 120.6 | 5 | 3.9 | 4.8 |
| Isazophos | C_9_H_17_ClN_3_O_3_PS | 14.07 | 314.04895 | Confirmed | 2.32_X_10^-2^ | 4.36_X_10^-2^ | 0.9889 | 124.6 |  | 131.0 | 131.2 | 5 | 7.2 | 5.1 |
| Isocarbophos | C_11_H_16_NO_4_PS | 16.46 | 230.98754 | Quan only | 7.29_X_10^-3^ | 2.25_X_10^-2^ | 0.9876 | 109.2 |  | 138.6 | 139.5 | 5 | 14.1 | 16.7 |
| Isofenphos | C_15_H_24_NO_4_PS | 18.02 | 346.12364 | Confirmed | -2.15_X_10^-4^ | 5.79_X_10^-3^ | 0.9934 | 68.0 |  | 104.3 | 108.6 | 5 | 15.7 | 8.0 |
| Isopropalin | C_15_H_23_N_3_O_4_ | 22.02 | 310.17613 | Confirmed | -3.22_X_10^-3^ | 1.29_X_10^-2^ | 0.9904 | 88.1 |  | 101.4 | 94.5 | 5 | 5.3 | 12.3 |
| Isoprothiolane | C_12_H_18_O_4_S_2_ | 13.73 | 291.07193 | Confirmed | 4.78_X_10^-2^ | 4.40_X_10^-2^ | 0.9893 | 112.8 | * | 129.7 | 128.2 | 10 | 2.6 | 3.8 |
| Isoproturon | C_12_H_18_N_2_O | 10.42 | 207.14919 | Check ID | 6.53_X_10^-2^ | 6.47_X_10^-2^ | 0.9900 | 127.4 | * | 131.4 | 126.5 | 10 | 1.9 | 4.0 |
| Isouron | C_1_0H_17_N_3_O_2_ | 8.22 | 212.13935 | Confirmed | 2.08_X_10^-2^ | 5.32_X_10^-2^ | 0.9889 | 100.7 |  | 103.0 | 110.6 | 5 | 5.4 | 7.6 |
| Isoxaben | C_18_H_24_N_2_O_4_ | 13.49 | 333.18088 | Confirmed | 1.52_X_10^-2^ | 2.93_X_10^-2^ | 0.9859 | 107.2 | * | 122.7 | 118.8 | 10 | 2.8 | 3.6 |
| Isoxadifen-ethyl | C_18_H_17_NO_3_ | 16.48 | 296.12812 | Confirmed | 4.97_X_10^-2^ | 3.00_X_10^-2^ | 0.9914 | 116.0 |  | 107.2 | 99.5 | 5 | 10.4 | 5.9 |
| Isoxathion | C_13_H_16_NO_4_PS | 18.43 | 314.06104 | Confirmed | 6.01_X_10^-2^ | 5.49_X_10^-2^ | 0.9922 | 85.4 | * | 107.0 | 106.5 | 10 | 3.1 | 4.0 |
| Karbutilate | C_14_H_21_N_3_O_3_ | 7.60 | 280.16557 | Confirmed | 2.50_X_10^-2^ | 2.96_X_10^-2^ | 0.9857 | 125.2 |  | 132.9 | 131.3 | 5 | 3.5 | 4.1 |
| Kresoxim Methyl | C_18_H_19_NO_4_ | 16.38 | 314.13868 | Confirmed | 2.69_X_10^-2^ | 2.33_X_10^-2^ | 0.9924 | 130.5 | * | 143.1 | 135.8 | 10 | 4.1 | 4.9 |
| Linuron | C_9_H_1_0Cl_2_N_2_O_2_ | 12.73 | 249.01921 | Confirmed | -5.42_X_10^-3^ | 1.13_X_10^-2^ | 0.9884 | 81.2 |  | 103.5 | 112.9 | 5 | 11.5 | 15.5 |
| Malathion | C_1_0H_19_O_6_PS_2_ | 13.47 | 331.04334 | Confirmed | 1.32_X_10^-2^ | 2.57_X_10^-2^ | 0.9888 | 112.2 |  | 120.8 | 120.6 | 5 | 5.1 | 6.8 |
| Malathion-oxon | C_1_0H_19_O_7_PS | 7.75 | 315.06619 | Confirmed | 2.39_X_10^-2^ | 3.32_X_10^-2^ | 0.9891 | 108.4 | * | 120.1 | 117.9 | 10 | 4.1 | 4.1 |
| Mandipropamid | C_23_H_22_ClNO_4_ | 13.73 | 412.13101 | Confirmed | 7.36_X_10^-3^ | 1.04_X_10^-2^ | 0.9838 | 95.3 |  | 117.2 | 122.0 | 5 | 9.9 | 7.3 |
| Mecarbam | C_1_0H_2_0NO_5_PS_2_ | 14.79 | 330.05933 | Check ID | 2.68_X_10^-2^ | 2.99_X_10^-2^ | 0.9902 | 73.4 |  | 91.9 | 93.7 | 5 | 8.2 | 6.1 |
| Mefenacet | C_16_H_14_N_2_O_2_S | 14.62 | 299.08487 | Confirmed | 2.91_X_10^-2^ | 4.77_X_10^-2^ | 0.9916 | 88.6 | * | 117.8 | 121.7 | 10 | 8.9 | 7.7 |
| Mefenpyr-diethyl | C_16_H_18_Cl_2_N_2_O_4_ | 17.47 | 373.07164 | Confirmed | 1.67_X_10^-2^ | 2.00_X_10^-2^ | 0.9919 | 78.5 |  | 99.2 | 97.4 | 5 | 10.4 | 6.4 |
| Mefluidide | C_11_H_13_F_3_N_2_O_3_S | 7.73 | 311.06717 | Confirmed | -1.38_X_10^-5^ | 7.82_X_10^-3^ | 0.9887 | 53.4 | * | 93.1 | 103.3 | 10 | 11.6 | 7.9 |
| Mepanipyrim | C_14_H_13_N_3_ | 15.43 | 224.11822 | Quan only | -1.98_X_10^-2^ | 6.12_X_10^-2^ | 0.9913 | 83.9 |  | 99.1 | 103.0 | 5 | 1.6 | 7.1 |
| Mephosfolan | C_8_H_16_NO_3_PS_2_ | 7.48 | 270.03820 | Confirmed | 3.56_X_10^-2^ | 3.71_X_10^-2^ | 0.9880 | 82.5 | * | 94.3 | 95.9 | 10 | 6.9 | 4.8 |
| Mepronil | C_17_H_19_NO_2_ | 13.47 | 270.14886 | Confirmed | 3.55_X_10^-2^ | 3.94_X_10^-2^ | 0.9880 | 121.2 |  | 117.8 | 118.3 | 5 | 8.8 | 7.8 |
| Metalaxyl | C_15_H_21_NO_4_ | 10.13 | 280.15433 | Confirmed | 5.07_X_10^-2^ | 1.05_X_10^-1^ | 0.9875 | 92.8 | * | 126.7 | 131.2 | 10 | 8.5 | 6.0 |
| Metazachlor | C_14_H_16_ClN_3_O | 10.10 | 278.10547 | Check ID | -3.81_X_10^-2^ | 3.21_X_10^-2^ | 0.9846 | 41.0 |  | 61.1 | 98.3 | 5 | 8.6 | 5.8 |
| Metconazole | C_17_H_22_ClN_3_O | 17.93 | 320.15242 | Check ID | 2.44_X_10^-3^ | 1.52_X_10^-2^ | 0.9916 | 52.1 |  | 85.0 | 87.1 | 5 | 10.8 | 11.9 |
| Methabenzthiazuron | C_1_0H_11_N_3_OS | 10.86 | 222.06956 | Confirmed | -6.69_X_10^-2^ | 3.12_X_10^-2^ | 0.9804 | 92.9 |  | 91.8 | 49.1 | 5 | 4.9 | 4.4 |
| Methacrifos | C_7_H_13_O_5_PS | 11.15 | 241.02941 | Confirmed | -1.56_X_10^-3^ | 8.91_X_10^-3^ | 0.9912 | 80.5 |  | 109.6 | 117.8 | 5 | 7.5 | 10.7 |
| Methidathion | C_6_H_11_N_2_O_4_PS_3_ | 10.95 | 302.96829 | Confirmed | 7.06_X_10^-3^ | 1.70_X_10^-2^ | 0.9876 | 91.0 |  | 103.4 | 104.8 | 5 | 7.0 | 5.7 |
| Methomyl | C_5_H_1_0N_2_O_2_S | 4.45 | 106.03211 | Quan only | -3.98_X_10^-2^ | 1.63_X_10^-2^ | 0.9449 | 52.4 |  | 31.1 | 28.5 | 5 | 1.0 | 6.2 |
| Methoprotryne | C_11_H_21_N_5_OS | 10.29 | 272.15396 | Confirmed | 5.98_X_10^-2^ | 6.34_X_10^-2^ | 0.9889 | 125.3 | * | 141.3 | 136.3 | 10 | 2.4 | 4.1 |
| Methoxyfenozide | C_22_H_28_N_2_O_3_ | 14.04 | 369.21727 | Confirmed | 8.98_X_10^-3^ | 1.46_X_10^-2^ | 0.9869 | 131.6 |  | 131.6 | 127.6 | 5 | 6.9 | 4.8 |
| Methyl Paraoxon | C_8_H_1_0NO_6_P | 10.91 | 248.03185 | Check ID | -1.71_X_10^-2^ | 2.11_X_10^-2^ | 0.9909 | 38.0 |  | 41.5 | 64.3 | 5 | 19.0 | 10.6 |
| Metobromuron | C_9_H_11_BrN_2_O_2_ | 9.94 | 259.00767 | Confirmed | 1.96_X_10^-3^ | 1.50_X_10^-2^ | 0.9871 | 73.0 |  | 90.6 | 98.0 | 5 | 5.8 | 10.0 |
| Metolachlor | C_15_H_22_ClNO_2_ | 15.11 | 284.14118 | Confirmed | 5.77_X_10^-2^ | 9.05_X_10^-2^ | 0.9909 | 100.8 |  | 114.1 | 116.0 | 5 | 4.6 | 4.3 |
| Metominostrobin, E- | C_16_H_16_N_2_O_3_ | 10.77 | 285.12337 | Confirmed | 3.08_X_10^-2^ | 3.72_X_10^-2^ | 0.9888 | 123.5 | * | 134.0 | 128.3 | 10 | 2.2 | 3.7 |
| Metominostrobin, Z- | C_16_H_16_N_2_O_3_ | 8.80 | 285.12337 | Check ID | 1.61_X_10^-2^ | 2.86_X_10^-2^ | 0.9870 | ** |  | 115.2 | 114.4 | 10 | 2.3 | 4.4 |
| Metosulam | C_14_H_13_Cl_2_N_5_O_4_S | 8.48 | 418.01381 | Quan only | -1.65_X_10^-3^ | 4.71_X_10^-3^ | 0.9867 | 42.9 |  | 90.0 | 97.9 | 5 | 24.5 | 19.9 |
| Metoxuron | C_1_0H_13_ClN_2_O_2_ | 6.63 | 229.07383 | Confirmed | 2.42_X_10^-3^ | 2.03_X_10^-2^ | 0.9899 | 56.1 | * | 86.2 | 93.9 | 10 | 7.5 | 5.2 |
| Metrafenone | C_19_H_21_BrO_5_ | 18.44 | 409.06451 | Confirmed | 8.55_X_10^-3^ | 1.53_X_10^-2^ | 0.9927 | 74.0 |  | 90.7 | 88.1 | 5 | 6.1 | 8.1 |
| Metribuzin | C_8_H_14_N_4_OS | 7.33 | 215.09611 | Confirmed | -3.82_X_10^-2^ | 2.00_X_10^-2^ | 0.9875 | ** |  | 47.8 | 83.6 | 10 | 17.8 | 17.3 |
| Metsulfuron-methyl | C_14_H_15_N_5_O_6_S | 8.05 | 382.08158 | Quan only | -1.42_X_10^-3^ | 8.04_X_10^-3^ | 0.9813 | 41.1 |  | 79.1 | 90.7 | 5 | 22.8 | 12.5 |
| Mevinphos | C_7_H_13_O_6_P | 5.24 | 225.05225 | Check ID | -4.48_X_10^-2^ | 2.56_X_10^-2^ | 0.9898 | ** |  | 32.3 | 42.2 | 10 | 12.3 | 8.3 |
| Molinate | C_9_H_17_NOS | 13.65 | 188.11036 | Quan only | -1.23_X_10^-2^ | 3.08_X_10^-2^ | 0.9897 | 43.2 |  | 85.0 | 95.2 | 5 | 11.5 | 13.5 |
| Monolinuron | C_9_H_11_ClN_2_O_2_ | 9.09 | 215.05818 | Confirmed | 5.49_X_10^-3^ | 2.42_X_10^-2^ | 0.9883 | 77.7 | * | 97.6 | 97.9 | 10 | 3.7 | 3.6 |
| Monuron | C_9_H_11_ClN_2_O | 7.72 | 199.06327 | Check ID | -1.73_X_10^-2^ | 2.64_X_10^-2^ | 0.9886 | ** |  | 65.3 | 84.1 | 10 | 7.5 | 17.8 |
| Myclobutanil | C_15_H_17_ClN_4_ | 14.20 | 289.12145 | Confirmed | 7.03_X_10^-3^ | 1.27_X_10^-2^ | 0.9921 | 104.9 |  | 123.2 | 121.3 | 5 | 11.0 | 7.0 |
| Naphthalophos (naftalofos) | C_16_H_16_NO_6_P | 14.19 | 350.07880 | Confirmed | 3.14_X_10^-2^ | 2.99_X_10^-2^ | 0.9886 | 46.6 | * | 69.3 | 72.0 | 10 | 1.6 | 4.7 |
| Napropamide | C_17_H_21_NO_2_ | 15.39 | 272.16451 | Confirmed | 5.54_X_10^-2^ | 6.76_X_10^-2^ | 0.9910 | 107.9 | * | 128.2 | 124.5 | 10 | 0.5 | 3.9 |
| Naptalam | C_18_H_13_NO_3_ | 7.78 | 292.09682 | Confirmed | -7.69_X_10^-3^ | 2.99_X_10^-3^ | 0.9871 | ** |  | 37.0 | 48.9 | 10 | 5.1 | 4.7 |
| Neburon | C_12_H_16_Cl_2_N_2_O | 16.36 | 275.07125 | Check ID | 3.67_X_10^-2^ | 2.56_X_10^-2^ | 0.9920 | 116.0 |  | 126.8 | 125.3 | 5 | 0.6 | 3.7 |
| Nicosulfuron | C_15_H_18_N_6_O_6_S | 9.58 | 411.10813 | Quan only | 1.46_X_10^-2^ | 1.27_X_10^-2^ | 0.9861 | 147.6 |  | 145.9 | 138.8 | 5 | 1.9 | 5.2 |
| Nitenpyram | C_11_H_15_ClN_4_O_2_ | 4.28 | 271.09563 | Confirmed | -1.11_X_10^-2^ | 7.29_X_10^-3^ | 0.9842 | 45.2 |  | 21.5 | 10.4 | 5 | 6.3 | 5.8 |
| Norflurazon | C_12_H_9_ClF_3_N_3_O | 10.98 | 304.04590 | Confirmed | 1.11_X_10^-2^ | 2.20_X_10^-2^ | 0.9880 | 82.1 | * | 98.7 | 99.9 | 10 | 4.0 | 3.7 |
| Norflurazon-desmethyl | C_11_H_7_ClF_3_N_3_O | 9.35 | 290.03025 | Confirmed | 3.54_X_10^-3^ | 1.40_X_10^-2^ | 0.9884 | 83.2 |  | 95.9 | 97.6 | 5 | 6.6 | 7.8 |
| Nuarimol | C_17_H_12_ClFN_2_O | 12.51 | 315.06950 | Confirmed | 1.81_X_10^-3^ | 7.17_X_10^-3^ | 0.9896 | 119.5 |  | 126.8 | 124.1 | 5 | 8.9 | 5.3 |
| Ochratoxina A‡ | C_2_0H_18_ClNO_6_ | 14.50 | 257.02069 | Confirmed | 1.04_X_10^-2^ | 4.02_X_10^-1^ | 0.9918 | 69.4 |  | 74.0 | 75.7 | 5 | 12.0 | 4.3 |
| Ofurace | C_14_H_16_ClNO_3_ | 7.71 | 282.08915 | Confirmed | -8.71_X_10^-3^ | 1.34_X_10^-2^ | 0.9849 | 57.3 |  | 85.6 | 97.5 | 5 | 11.1 | 7.5 |
| Orbencarb | C_12_H_16_ClNOS | 18.02 | 258.07139 | Confirmed | -7.01_X_10^-2^ | 4.89_X_10^-2^ | 0.9571 | 59.9 |  | 72.4 | 72.9 | 5 | 5.9 | 4.8 |
| Oxadiargyl | C_15_H_14_Cl_2_N_2_O_3_ | 17.83 | 341.04542 | Quan only | -1.33_X_10^-3^ | 1.60_X_10^-3^ | 0.9887 | ** |  | 37.9 | 71.8 | 10 | 20.0 | 19.1 |
| Oxadiazon | C_15_H_18_Cl_2_N_2_O_3_ | 20.38 | 345.07672 | Confirmed | -1.91_X_10^-3^ | 2.43_X_10^-3^ | 0.9899 | ** |  | 32.5 | 43.7 | 10 | 29.2 | 15.6 |
| Oxasulfuron | C_17_H_18_N_4_O_6_S | 7.57 | 407.10198 | Quan only | -5.29_X_10^-3^ | 5.16_X_10^-3^ | 0.9867 | 34.8 |  | 73.4 | 83.7 | 5 | 18.1 | 6.9 |
| Oxaziclomefone | C_2_0H_19_Cl_2_NO_2_ | 19.91 | 376.08656 | Confirmed | 1.84_X_10^-2^ | 2.16_X_10^-2^ | 0.9898 | 41.1 |  | 71.1 | 71.1 | 5 | 4.7 | 17.8 |
| Oxycarboxin | C_12_H_13_NO_4_S | 5.64 | 268.06381 | Confirmed | -1.37_X_10^-2^ | 1.02_X_10^-2^ | 0.9796 | 65.2 |  | 58.9 | 44.6 | 5 | 32.8 | 14.4 |
| Oxyfluorfen | C_15_H_11_ClF_3_NO_4_ | 16.11 | 362.04015 | Quan only | -1.45_X_10^-3^ | 3.63_X_10^-3^ | 0.9895 | 65.2 |  | 127.8 | 132.1 | 5 | 15.1 | 10.9 |
| Paclobutrazol | C_15_H_2_0ClN_3_O | 13.26 | 294.13677 | Check ID | -2.49_X_10^-3^ | 1.85_X_10^-2^ | 0.9897 | 49.1 |  | 98.5 | 111.4 | 5 | 13.0 | 13.8 |
| Parathion-oxon | C_1_0H_14_NO_6_P | 9.84 | 276.06315 | Confirmed | -2.85_X_10^-3^ | 1.24_X_10^-2^ | 0.9863 | 80.2 |  | 98.6 | 102.7 | 5 | 9.5 | 10.2 |
| Penconazol | C_13_H_15_Cl_2_N_3_ | 17.13 | 284.07158 | Confirmed | 1.35_X_10^-2^ | 1.93_X_10^-2^ | 0.9919 | 103.2 |  | 114.6 | 113.9 | 5 | 8.7 | 6.1 |
| Pencycuron | C_19_H_21_ClN_2_O | 18.63 | 329.14152 | Confirmed | 2.02_X_10^-2^ | 2.69_X_10^-2^ | 0.9897 | 80.6 |  | 95.1 | 94.2 | 5 | 7.0 | 6.1 |
| Pendimethalin | C_13_H_19_N_3_O_4_ | 21.13 | 282.14483 | Confirmed | -3.66_X_10^-3^ | 1.29_X_10^-2^ | 0.9885 | 72.7 |  | 83.6 | 83.4 | 5 | 6.7 | 6.7 |
| Pentanochlor | C_13_H_18_ClNO | 16.01 | 240.11497 | Quan only | 1.99_X_10^-2^ | 4.03_X_10^-2^ | 0.9897 | 67.9 |  | 81.9 | 97.1 | 5 | 17.3 | 9.5 |
| Penthiopyrad | C_16_H_2_0F_3_N_3_OS | 17.05 | 360.13519 | Confirmed | 2.17_X_10^-2^ | 3.37_X_10^-2^ | 0.9889 | 93.4 | * | 114.8 | 113.5 | 10 | 3.2 | 4.8 |
| Phenothrin | C_23_H_26_O_3_ | 23.01 | 351.19547 | Check ID | -5.94_X_10^-3^ | 4.85_X_10^-3^ | 0.8999 | 44.2 |  | 50.3 | 55.3 | 5 | 21.9 | 10.3 |
| Phenthoate | C_12_H_17_O_4_PS_2_ | 16.50 | 321.03786 | Confirmed | 2.28_X_10^-2^ | 2.41_X_10^-2^ | 0.9910 | 104.4 |  | 125.1 | 123.9 | 5 | 5.6 | 4.6 |
| Phorate | C_7_H_17_O_2_PS_3_ | 17.73 | 261.02010 | Check ID | -5.86_X_10^-3^ | 2.78_X_10^-3^ | 0.9782 | 59.6 |  | 92.4 | 85.6 | 5 | 7.7 | 6.7 |
| Phorate oxon | C_7_H_17_O_3_PS_2_ | 10.81 | 245.04295 | Confirmed | 1.93_X_10^-2^ | 3.36_X_10^-2^ | 0.9897 | 59.9 | * | 90.3 | 91.9 | 10 | 7.1 | 5.3 |
| Phorate Sulfoxide | C_7_H_17_O_3_PS_3_ | 9.41 | 277.01502 | Confirmed | 1.86_X_10^-2^ | 3.61_X_10^-2^ | 0.9903 | 101.8 | * | 121.7 | 119.4 | 10 | 2.4 | 3.7 |
| Phorate-oxon Sulfone | C_7_H_17_O_5_PS_2_ | 5.51 | 277.03278 | Confirmed | -7.31_X_10^-3^ | 2.24_X_10^-2^ | 0.9876 | 60.8 |  | 62.2 | 78.8 | 5 | 12.5 | 17.3 |
| Phorate-sulfone | C_7_H_17_O_4_PS_3_ | 9.58 | 293.00993 | Confirmed | 5.02_X_10^-3^ | 1.42_X_10^-2^ | 0.9898 | 92.5 |  | 110.5 | 113.5 | 5 | 6.2 | 14.0 |
| Phosalone | C_12_H_15_ClNO_4_PS_2_ | 18.24 | 367.99414 | Confirmed | 3.75_X_10^-3^ | 8.94_X_10^-3^ | 0.9913 | 65.7 |  | 89.5 | 92.1 | 5 | 6.7 | 7.6 |
| Phosphamidon | C_1_0H_19_ClNO_5_P | 6.69 | 300.07621 | Confirmed | -7.06_X_10^-2^ | 1.79_X_10^-2^ | 0.9209 | ** |  | 54.6 | 37.7 | 10 | 5.2 | 7.7 |
| Phoxim | C_12_H_15_N_2_O_3_PS | 16.65 | 299.06138 | Confirmed | 1.27_X_10^-2^ | 3.80_X_10^-2^ | 0.9915 | 86.1 | * | 105.3 | 105.9 | 10 | 5.0 | 5.2 |
| Picolinafen | C_19_H_12_F_4_N_2_O_2_ | 20.93 | 377.09077 | Confirmed | 7.61_X_10^-3^ | 1.65_X_10^-2^ | 0.9881 | 72.8 |  | 81.7 | 84.4 | 5 | 3.9 | 4.0 |
| Piperalin | C_16_H_21_Cl_2_NO_2_ | 9.48 | 330.10221 | Quan only | 3.28_X_10^-2^ | 2.82_X_10^-2^ | 0.9869 | 33.3 |  | 51.6 | 47.9 | 5 | 21.2 | 19.2 |
| Piperonyl-butoxide | C_19_H_3_0O_5_ | 20.38 | 356.24315 | Confirmed | 3.34_X_10^-2^ | 4.71_X_10^-2^ | 0.9903 | 75.3 | * | 93.9 | 91.3 | 10 | 3.3 | 8.2 |
| Piperophos | C_14_H_28_NO_3_PS_2_ | 19.03 | 354.13210 | Confirmed | 6.62_X_10^-2^ | 4.99_X_10^-2^ | 0.9935 | 142.3 |  | 152.9 | 143.9 | 5 | 3.8 | 4.4 |
| Pirimicarb | C_11_H_18_N_4_O_2_ | 6.49 | 239.15025 | Confirmed | 2.28_X_10^-2^ | 5.05_X_10^-2^ | 0.9903 | 92.4 | * | 111.2 | 113.6 | 10 | 3.2 | 4.0 |
| Pirimiphos Methyl | C_11_H_2_0N_3_O_3_PS | 17.25 | 306.10358 | Confirmed | -1.16_X_10^-2^ | 4.69_X_10^-2^ | 0.9888 | 60.2 |  | 95.3 | 93.9 | 5 | 18.0 | 16.7 |
| Pirimiphos-ethyl | C_13_H_24_N_3_O_3_PS | 19.98 | 334.13488 | Confirmed | 2.68_X_10^-2^ | 5.23_X_10^-2^ | 0.9894 | 55.6 |  | 80.3 | 80.8 | 5 | 10.9 | 11.2 |
| Pirimiphos-methyl-n-desethyl | C_9_H_16_N_3_O_3_PS | 10.13 | 278.07228 | Confirmed | 2.74_X_10^-2^ | 4.11_X_10^-2^ | 0.9868 | 106.2 | * | 122.0 | 121.9 | 10 | 3.0 | 4.5 |
| Prallethrin | C_19_H_24_O_3_ | 18.92 | 301.17982 | Confirmed | 3.86_X_10^-2^ | 2.03_X_10^-2^ | 0.9894 | 122.2 |  | 120.9 | 111.4 | 5 | 8.0 | 5.5 |
| Pretilachlor | C_17_H_26_ClNO_2_ | 19.21 | 312.17248 | Confirmed | -3.84_X_10^-2^ | 4.51_X_10^-2^ | 0.9907 | 87.8 |  | 102.5 | 106.3 | 5 | 3.0 | 4.7 |
| Prochloraz | C_15_H_16_Cl_3_N_3_O_2_ | 17.97 | 376.03809 | Confirmed | 9.93_X_10^-3^ | 1.51_X_10^-2^ | 0.9928 | 87.0 |  | 105.2 | 104.0 | 5 | 9.0 | 5.6 |
| Profenofos | C_11_H_15_BrClO_3_PS | 19.91 | 372.94242 | Check ID | 8.02_X_10^-3^ | 7.97_X_10^-3^ | 0.9918 | ** |  | 44.3 | 54.9 | 10 | 17.3 | 18.6 |
| Promecarb | C_12_H_17_NO_2_ | 12.99 | 208.13321 | Confirmed | 4.37_X_10^-2^ | 4.69_X_10^-2^ | 0.9881 | ** |  | 138.4 | 124.2 | 10 | 3.2 | 3.8 |
| Prometon | C_1_0H_19_N_5_O | 8.59 | 226.16624 | Confirmed | 2.08_X_10^-1^ | 2.23_X_10^-1^ | 0.9897 | 109.4 | * | 125.3 | 121.5 | 10 | 2.1 | 3.8 |
| Prometryn | C_1_0H_19_N_5_S | 12.00 | 242.14339 | Confirmed | 3.21_X_10^-2^ | 6.77_X_10^-2^ | 0.9881 | 90.4 |  | 105.2 | 108.4 | 5 | 5.5 | 3.4 |
| Propachlor | C_11_H_14_ClNO | 10.04 | 212.08367 | Confirmed | 1.91_X_10^-2^ | 5.19_X_10^-2^ | 0.9887 | 90.4 | * | 105.3 | 107.4 | 10 | 1.0 | 3.5 |
| Propaphos | C_13_H_21_O_4_PS | 17.13 | 305.09709 | Confirmed | 2.58_X_10^-2^ | 2.86_X_10^-2^ | 0.9789 | 106.8 | * | 120.1 | 116.7 | 10 | 7.0 | 8.2 |
| Propargite | C_19_H_26_O_4_S | 21.33 | 368.18901 | Confirmed | 1.84_X_10^-2^ | 2.49_X_10^-2^ | 0.9913 | 33.4 | * | 51.2 | 56.4 | 10 | 7.5 | 11.6 |
| Propazine | C_9_H_16_ClN_5_ | 11.98 | 146.02263 | Confirmed | 9.32_X_10^-3^ | 1.14_X_10^-2^ | 0.9889 | 105.8 | * | 117.4 | 113.4 | 10 | 2.9 | 4.8 |
| Propetamphos | C_1_0H_2_0NO_4_PS | 13.90 | 282.09234 | Confirmed | 4.74_X_10^-3^ | 1.20_X_10^-2^ | 0.9909 | 92.1 |  | 110.0 | 114.7 | 5 | 3.2 | 9.3 |
| Propiconazol (mixture Of Isomers) | C_15_H_17_Cl_2_N_3_O_2_ | 17.54 | 342.07706 | Confirmed | -4.67_X_10^-3^ | 1.16_X_10^-2^ | 0.9068 | 57.4 |  | 72.1 | 90.2 | 5 | 8.5 | 7.2 |
| Propisochlor | C_15_H_22_ClNO_2_ | 15.11 | 284.14118 | Confirmed | 5.77_X_10^-2^ | 9.05_X_10^-2^ | 0.9909 | 100.8 |  | 114.1 | 116.0 | 5 | 4.6 | 4.3 |
| Propyzamide (pronamide) | C_12_H_11_Cl_2_NO | 13.02 | 256.02905 | Confirmed | -7.78_X_10^-3^ | 7.65_X_10^-3^ | 0.9877 | 76.5 |  | 84.0 | 97.1 | 5 | 7.8 | 12.5 |
| Proquinazid | C_14_H_17_IN_2_O_2_ | 22.01 | 373.04075 | Confirmed | 2.38_X_10^-2^ | 3.51_X_10^-2^ | 0.9913 | 55.5 |  | 65.1 | 64.9 | 5 | 2.2 | 7.4 |
| Prosulfocarb | C_14_H_21_NOS | 19.44 | 252.14166 | Quan only | 2.69_X_10^-2^ | 5.00_X_10^-2^ | 0.9915 | 71.6 | * | 84.9 | 85.5 | 10 | 7.0 | 5.5 |
| Prosulfuron | C_15_H_16_F_3_N_5_O_4_S | 12.88 | 420.09479 | Confirmed | 4.54_X_10^-3^ | 7.86_X_10^-3^ | 0.9890 | 99.7 |  | 124.5 | 125.0 | 5 | 6.3 | 7.5 |
| Prothioconazole | C_14_H_15_Cl_2_N_3_OS | 17.32 | 344.03856 | Confirmed | -3.17_X_10^-3^ | 1.26_X_10^-3^ | 0.9864 | ** |  | 37.5 | 62.2 | 10 | 6.1 | 7.7 |
| Pyraclofos | C_14_H_18_ClN_2_O_3_PS | 18.39 | 361.05370 | Confirmed | 2.56_X_10^-2^ | 2.19_X_10^-2^ | 0.9902 | 82.0 |  | 98.2 | 97.6 | 5 | 7.7 | 7.0 |
| Pyraclostrobin | C_19_H_18_ClN_3_O_4_ | 18.32 | 388.10586 | Confirmed | 2.37_X_10^-2^ | 3.65_X_10^-2^ | 0.9918 | 107.9 | * | 121.3 | 116.4 | 10 | 3.8 | 5.0 |
| Pyraflufen-ethyl | C_15_H_13_Cl_2_F_3_N_2_O_4_ | 17.57 | 413.02772 | Confirmed | 1.00_X_10^-2^ | 1.37_X_10^-2^ | 0.9922 | 51.8 | * | 76.8 | 78.8 | 10 | 3.3 | 8.8 |
| Pyrazolynate | C_19_H_16_Cl_2_N_2_O_4_S | 18.92 | 439.02806 | Quan only | 6.50_X_10^-3^ | 1.13_X_10^-2^ | 0.9890 | 47.6 |  | 72.7 | 72.2 | 5 | 9.5 | 19.3 |
| Pyrazophos | C_14_H_2_0N_3_O_5_PS | 18.39 | 374.09340 | Confirmed | 2.27_X_10^-2^ | 2.06_X_10^-2^ | 0.9921 | 87.8 | * | 111.4 | 110.2 | 10 | 3.1 | 4.7 |
| Pyrazosulfuron-ethyl | C_14_H_18_N_6_O_7_S | 14.77 | 415.10304 | Check ID | 5.31_X_10^-3^ | 9.87_X_10^-3^ | 0.9902 | 110.8 |  | 133.9 | 137.1 | 5 | 10.4 | 6.0 |
| Pyrazoxyfen | C_2_0H_16_Cl_2_N_2_O_3_ | 17.46 | 403.06107 | Confirmed | 7.63_X_10^-3^ | 1.24_X_10^-2^ | 0.9907 | 69.6 |  | 91.7 | 88.6 | 5 | 2.4 | 7.8 |
| Pyributicarb | C_18_H_22_N_2_O_2_S | 20.69 | 331.14748 | Confirmed | 2.98_X_10^-2^ | 4.76_X_10^-2^ | 0.9898 | 75.9 | * | 91.0 | 89.3 | 10 | 8.9 | 7.8 |
| Pyridaben | C_19_H_25_ClN_2_OS | 22.36 | 365.14489 | Confirmed | 3.82_X_10^-2^ | 3.31_X_10^-2^ | 0.9774 | 99.8 |  | 102.3 | 103.4 | 5 | 8.8 | 6.9 |
| Pyridafol | C_1_0H_7_ClN_2_O | 6.63 | 207.03197 | Confirmed | 4.80_X_10^-3^ | 1.44_X_10^-2^ | 0.9903 | 40.5 | * | 86.2 | 90.8 | 10 | 7.9 | 5.5 |
| Pyridaphenthion | C_14_H_17_N_2_O_4_PS | 14.48 | 341.07194 | Confirmed | 2.57_X_10^-2^ | 2.55_X_10^-2^ | 0.9895 | 100.2 |  | 113.8 | 115.8 | 5 | 3.3 | 4.1 |
| Pyridate | C_19_H_23_ClN_2_O_2_S | 23.00 | 379.12415 | Confirmed | 4.40_X_10^-3^ | 1.69_X_10^-2^ | 0.9928 | 33.2 |  | 40.6 | 45.7 | 5 | 14.0 | 7.9 |
| Pyrifenox | C_14_H_12_Cl_2_N_2_O | 13.96 | 295.03994 | Confirmed | 4.14_X_10^-3^ | 1.43_X_10^-2^ | 0.9884 | 82.1 |  | 99.0 | 102.0 | 5 | 2.3 | 7.4 |
| Pyrimethanil | C_12_H_13_N_3_ | 11.79 | 200.11822 | Confirmed | 3.15_X_10^-2^ | 5.77_X_10^-2^ | 0.9861 | 103.2 |  | 106.4 | 104.6 | 5 | 4.4 | 3.8 |
| Pyriproxyfen | C_2_0H_19_NO_3_ | 20.84 | 322.14377 | Confirmed | 4.56_X_10^-2^ | 4.94_X_10^-2^ | 0.9926 | 80.0 | * | 92.0 | 91.4 | 10 | 9.3 | 11.0 |
| Pyroxsulam | C_14_H_13_F_3_N_6_O_5_S | 7.90 | 435.06930 | Quan only | 2.98_X_10^-3^ | 1.19_X_10^-2^ | 0.9892 | 82.4 |  | 109.5 | 109.1 | 5 | 9.3 | 8.2 |
| Quinalphos | C_12_H_15_N_2_O_3_PS | 16.65 | 299.06138 | Confirmed | 1.27_X_10^-2^ | 3.80_X_10^-2^ | 0.9915 | 86.1 | * | 105.3 | 105.9 | 10 | 5.0 | 5.2 |
| Quinoxyfen | C_15_H_8_Cl_2_FNO | 21.40 | 308.00397 | Check ID | 7.62_X_10^-3^ | 2.14_X_10^-2^ | 0.9909 | 58.0 |  | 61.8 | 66.7 | 5 | 15.8 | 7.6 |
| Quizalofop-ethyl | C_19_H_17_ClN_2_O_4_ | 19.93 | 373.09496 | Confirmed | 4.05_X_10^-2^ | 3.55_X_10^-2^ | 0.9898 | 33.3 | * | 61.5 | 64.9 | 10 | 6.8 | 7.8 |
| Resmethrin | C_22_H_26_O_3_ | 22.65 | 339.19547 | Confirmed | 9.02_X_10^-3^ | 3.40_X_10^-2^ | 0.9817 | 65.1 | * | 74.8 | 74.4 | 10 | 10.5 | 8.0 |
| Rimsulfuron | C_14_H_17_N_5_O_7_S_2_ | 9.14 | 432.06422 | Quan only | -3.72_X_10^-3^ | 2.81_X_10^-3^ | 0.9910 | ** |  | 54.2 | 86.3 | 10 | 11.8 | 16.6 |
| Rotenone | C_23_H_22_O_6_ | 16.36 | 395.14891 | Confirmed | 1.29_X_10^-2^ | 1.22_X_10^-2^ | 0.9918 | 95.7 | * | 112.7 | 104.9 | 10 | 4.8 | 5.9 |
| Sebuthylazin | C_9_H_16_ClN_5_ | 11.69 | 230.11670 | Check ID | 1.04_X_10^-1^ | 6.12_X_10^-2^ | 0.9898 | ** |  | 139.6 | 122.7 | 10 | 0.8 | 4.3 |
| Secbumeton | C_1_0H_19_N_5_O | 8.59 | 226.16624 | Quan only | 2.08_X_10^-1^ | 2.23_X_10^-1^ | 0.9897 | 109.4 | * | 125.3 | 121.5 | 10 | 2.1 | 3.8 |
| Sethoxydim | C_17_H_29_NO_3_S | 19.93 | 328.19409 | Confirmed | 2.58_X_10^-2^ | 2.56_X_10^-2^ | 0.9921 | 58.8 |  | 84.3 | 89.4 | 5 | 8.8 | 18.2 |
| Siduron | C_14_H_2_0N_2_O | 12.14 | 233.16484 | Confirmed | 2.35_X_10^-2^ | 3.91_X_10^-2^ | 0.9853 | 101.2 | * | 111.1 | 108.0 | 10 | 2.4 | 3.6 |
| Silthiofam | C_13_H_21_NOSSi | 16.06 | 268.11859 | Confirmed | 1.76_X_10^-2^ | 1.47_X_10^-2^ | 0.9911 | 87.3 | * | 114.6 | 114.8 | 10 | 5.9 | 6.2 |
| Simeconazole | C_14_H_2_0FN_3_OSi | 14.92 | 294.14324 | Check ID | -2.73_X_10^-4^ | 2.07_X_10^-2^ | 0.9925 | 82.4 |  | 106.8 | 112.5 | 5 | 9.3 | 7.5 |
| Simetone | C_8_H_15_N_5_O | 5.77 | 198.13494 | Confirmed | -1.81_X_10^-1^ | 1.13_X_10^-1^ | 0.9695 | 88.7 |  | 76.3 | 67.8 | 5 | 5.4 | 15.2 |
| Simetryn | C_8_H_15_N_5_S | 8.07 | 214.11209 | Confirmed | 3.55_X_10^-2^ | 1.13_X_10^-1^ | 0.9872 | 85.7 |  | 100.8 | 104.6 | 5 | 6.1 | 6.0 |
| S-Metolachlor | C_15_H_22_ClNO_2_ | 15.11 | 284.14118 | Confirmed | 5.77_X_10^-2^ | 9.05_X_10^-2^ | 0.9909 | 100.8 |  | 114.1 | 116.0 | 5 | 4.6 | 4.3 |
| Spirodiclofen | C_21_H_24_Cl_2_O_4_ | 21.74 | 411.11244 | Confirmed | 6.09_X_10^-3^ | 8.68_X_10^-3^ | 0.9930 | 83.6 |  | 97.8 | 98.7 | 5 | 14.9 | 11.5 |
| Spiromesifen | C_23_H_3_0O_4_ | 21.30 | 388.24824 | Confirmed | 2.09_X_10^-2^ | 2.34_X_10^-2^ | 0.9911 | 48.3 | * | 64.3 | 67.0 | 10 | 2.3 | 12.5 |
| Spirotetramat | C_21_H_27_NO_5_ | 14.84 | 374.19620 | Confirmed | 3.43_X_10^-2^ | 2.76_X_10^-2^ | 0.9884 | 105.9 | * | 127.2 | 122.3 | 10 | 3.7 | 4.7 |
| Spirotetramat-enol | C_18_H_23_NO_3_ | 9.16 | 302.17507 | Confirmed | -2.18_X_10^-2^ | 1.41_X_10^-2^ | 0.9858 | 108.3 |  | 52.6 | 57.3 | 5 | 1.5 | 9.0 |
| Spirotetramat-mono-hydroxy | C_18_H_25_NO_3_ | 7.15 | 304.19072 | Confirmed | 4.83_X_10^-3^ | 1.37_X_10^-2^ | 0.9868 | 63.4 | * | 71.9 | 69.3 | 10 | 11.1 | 7.8 |
| Spiroxamine (mix Of Isomers) | C_18_H_35_NO_2_ | 13.16 | 298.27406 | Confirmed | 2.80_X_10^-2^ | 6.21_X_10^-2^ | 0.9905 | 95.9 |  | 94.7 | 86.2 | 5 | 8.4 | 7.3 |
| Sulfallate | C_8_H_14_ClNS_2_ | 16.55 | 224.03290 | Quan only | -6.12_X_10^-3^ | 1.16_X_10^-2^ | 0.9906 | 106.6 |  | 123.2 | 125.2 | 5 | 14.3 | 7.6 |
| Sulfometuron Methyl | C_15_H_16_N_4_O_5_S | 8.05 | 365.09142 | Quan only | -3.57_X_10^-3^ | 1.08_X_10^-2^ | 0.9802 | 86.4 |  | 86.4 | 98.6 | 5 | 8.1 | 11.8 |
| Sulprofos sulfoxide | C_12_H_19_O_3_PS_3_ | 15.36 | 339.03067 | Confirmed | 2.92_X_10^-2^ | 2.75_X_10^-2^ | 0.9916 | 94.9 |  | 109.0 | 109.0 | 5 | 5.1 | 5.4 |
| Tebuconazole | C_16_H_22_ClN_3_O | 16.98 | 308.15242 | Confirmed | 9.39_X_10^-3^ | 1.59_X_10^-2^ | 0.9903 | 112.3 |  | 126.9 | 121.9 | 5 | 5.8 | 6.3 |
| Tebufenozide | C_22_H_28_N_2_O_2_ | 16.30 | 353.22235 | Check ID | 8.34_X_10^-3^ | 1.40_X_10^-2^ | 0.9929 | 86.3 |  | 122.2 | 125.3 | 5 | 8.3 | 4.6 |
| Tebufenpyrad | C_18_H_24_ClN_3_O | 20.29 | 334.16807 | Confirmed | 1.40_X_10^-3^ | 1.36_X_10^-2^ | 0.9944 | ** |  | 27.6 | 35.7 | 10 | 11.2 | 12.6 |
| Tebupirimfos | C_13_H_23_N_2_O_3_PS | 20.12 | 319.12398 | Confirmed | 1.25_X_10^-2^ | 3.80_X_10^-2^ | 0.9925 | 63.5 |  | 76.9 | 77.4 | 5 | 5.5 | 4.5 |
| Tebutam | C_15_H_23_NO | 15.06 | 234.18524 | Quan only | 5.77_X_10^-2^ | 8.71_X_10^-2^ | 0.9888 | 105.4 | * | 120.8 | 119.9 | 10 | 1.8 | 3.8 |
| Tebuthiuron | C_9_H_16_N_4_OS | 8.09 | 229.11176 | Confirmed | -5.04_X_10^-3^ | 3.15_X_10^-2^ | 0.9890 | 34.8 | * | 69.6 | 81.7 | 10 | 6.3 | 6.1 |
| Tepraloxydim | C_17_H_24_ClNO_4_ | 14.85 | 342.14666 | Confirmed | -7.78_X_10^-3^ | 5.78_X_10^-3^ | 0.9892 | 58.5 |  | 85.2 | 99.8 | 5 | 11.6 | 15.3 |
| Terbucarb | C_17_H_27_NO_2_ | 18.77 | 278.21146 | Confirmed | 1.48_X_10^-2^ | 2.49_X_10^-2^ | 0.9926 | 41.3 | * | 94.4 | 107.2 | 10 | 6.9 | 10.0 |
| Terbufos | C_9_H_21_O_2_PS_3_ | 20.11 | 289.05141 | Check ID | 9.08_X_10^-5^ | 7.63_X_10^-3^ | 0.9934 | 38.3 |  | 78.1 | 82.2 | 5 | 21.1 | 11.5 |
| Terbufos-sulfon | C_9_H_21_O_4_PS_3_ | 11.86 | 321.04123 | Confirmed | 8.65_X_10^-3^ | 1.65_X_10^-2^ | 0.9884 | 122.5 |  | 130.6 | 129.1 | 5 | 3.6 | 4.5 |
| Terbufos-sulfoxide | C_9_H_21_O_3_PS_3_ | 12.09 | 305.04632 | Check ID | 3.86_X_10^-2^ | 3.45_X_10^-2^ | 0.9895 | 131.6 | * | 144.9 | 139.7 | 10 | 3.4 | 4.7 |
| Terbumeton | C_1_0H_19_N_5_O | 8.59 | 226.16624 | Confirmed | 2.08_X_10^-1^ | 2.23_X_10^-1^ | 0.9897 | 109.4 | * | 125.3 | 121.5 | 10 | 2.1 | 3.8 |
| Terbumeton-desethyl | C_8_H_15_N_5_O | 6.08 | 198.13494 | Check ID | 6.39_X_10^-2^ | 4.77_X_10^-2^ | 0.9876 | 88.4 |  | 93.7 | 92.6 | 5 | 1.9 | 3.4 |
| Terbutryne | C_1_0H_19_N_5_S | 12.62 | 242.14339 | Confirmed | 2.92_X_10^-2^ | 5.88_X_10^-2^ | 0.9894 | 103.9 |  | 113.5 | 115.4 | 5 | 4.1 | 5.0 |
| Tetrachlorvinphos (iso) | C_1_0H_9_Cl_4_O_4_P | 16.28 | 364.90653 | Confirmed | 3.82_X_10^-3^ | 6.07_X_10^-3^ | 0.9924 | 72.6 |  | 103.7 | 110.4 | 5 | 16.5 | 11.2 |
| Tetraconazole | C_13_H_11_Cl_2_F_4_N_3_O | 15.44 | 372.02881 | Confirmed | 8.56_X_10^-4^ | 7.45_X_10^-3^ | 0.9899 | 89.8 |  | 120.6 | 121.2 | 5 | 7.3 | 7.3 |
| Tetraethyl Dithiopyrophosphate | C_8_H_2_0O_5_P_2_S_2_ | 16.64 | 323.03001 | Confirmed | 1.56_X_10^-2^ | 4.50_X_10^-2^ | 0.9889 | 107.1 | * | 126.2 | 123.2 | 10 | 5.5 | 5.0 |
| Tetraethyl Pyrophosphate | C_8_H_2_0O_7_P_2_ | 6.77 | 291.07570 | Check ID | -6.05_X_10^-2^ | 1.73_X_10^-2^ | 0.9194 | ** |  | 41.5 | 27.6 | 10 | 0.2 | 3.7 |
| Tetramethrin | C_19_H_25_NO_4_ | 20.08 | 332.18563 | Confirmed | 1.45_X_10^-2^ | 1.66_X_10^-2^ | 0.9943 | ** |  | 40.8 | 44.8 | 10 | 8.4 | 6.8 |
| Thenylchlor | C_16_H_18_ClNO_2_S | 15.06 | 324.08195 | Confirmed | 1.93_X_10^-2^ | 2.05_X_10^-2^ | 0.9915 | 98.7 |  | 115.0 | 116.1 | 5 | 2.0 | 4.9 |
| Thiabendazole | C_1_0H_7_N_3_S | 5.29 | 202.04334 | Check ID | 1.40_X_10^-3^ | 2.26_X_10^-2^ | 0.9860 | ** |  | 29.4 | 71.6 | 10 | 34.3 | 19.1 |
| Thiacloprid | C_1_0H_9_ClN_4_S | 6.20 | 253.03092 | Check ID | 2.05_X_10^-4^ | 9.68_X_10^-3^ | 0.9833 | ** |  | 61.4 | 74.8 | 10 | 12.0 | 13.5 |
| Thiazafluron | C_6_H_7_F_3_N_4_OS | 7.90 | 241.03654 | Confirmed | -1.75_X_10^-3^ | 1.46_X_10^-2^ | 0.9893 | 72.9 |  | 96.7 | 97.9 | 5 | 10.4 | 7.9 |
| Thiazopyr | C_16_H_17_F_5_N_2_O_2_S | 16.88 | 397.10037 | Confirmed | 1.21_X_10^-2^ | 3.51_X_10^-2^ | 0.9914 | 90.0 | * | 110.7 | 110.1 | 10 | 3.5 | 3.9 |
| Thidiazuron | C_9_H_8_N_4_OS | 7.83 | 221.04916 | Check ID | -9.62_X_10^-3^ | 5.19_X_10^-3^ | 0.9920 | ** |  | 48.4 | 87.5 | 10 | 9.3 | 10.5 |
| Thifensulfuron-methyl | C_12_H_13_N_5_O_6_S_2_ | 7.63 | 388.03800 | Quan only | -2.01_X_10^-3^ | 5.89_X_10^-3^ | 0.9856 | 33.6 |  | 78.5 | 79.7 | 5 | 8.7 | 7.5 |
| Thiobencarb | C_12_H_16_ClNOS | 17.92 | 258.07139 | Check ID | -7.17_X_10^-2^ | 4.92_X_10^-2^ | 0.9559 | 60.0 |  | 72.3 | 72.9 | 5 | 5.9 | 4.8 |
| Thiodicarb | C_1_0H_18_N_4_O_4_S_3_ | 10.07 | 355.05629 | Quan only | 1.25_X_10^-2^ | 1.90_X_10^-2^ | 0.9849 | 57.7 | * | 84.2 | 85.3 | 10 | 3.2 | 4.5 |
| Thionazine (zinophos) | C_8_H_13_N_2_O_3_PS | 10.18 | 249.04573 | Confirmed | -1.53_X_10^-3^ | 2.37_X_10^-2^ | 0.9893 | 99.7 |  | 111.2 | 113.7 | 5 | 3.1 | 4.7 |
| Thiophanate | C_14_H_18_N_4_O_4_S_2_ | 10.54 | 371.08422 | Confirmed | -6.03_X_10^-3^ | 8.90_X_10^-3^ | 0.9895 | ** |  | 25.5 | 35.5 | 10 | 3.4 | 15.9 |
| Thiophanate-ethyl | C_14_H_18_N_4_O_4_S_2_ | 10.54 | 371.08422 | Confirmed | -6.03_X_10^-3^ | 8.90_X_10^-3^ | 0.9895 | ** |  | 25.5 | 35.5 | 10 | 3.4 | 15.9 |
| Thiophanate-methyl | C_12_H_14_N_4_O_4_S_2_ | 7.42 | 343.05292 | Quan only | -4.88_X_10^-3^ | 6.90_X_10^-3^ | 0.9874 | ** |  | 14.5 | 23.9 | 10 | 18.5 | 10.3 |
| Tralkoxydim | C_2_0H_27_NO_3_ | 20.77 | 330.20637 | Confirmed | 2.40_X_10^-2^ | 3.02_X_10^-2^ | 0.9914 | 129.0 |  | 126.3 | 120.4 | 5 | 13.2 | 7.7 |
| Triadimefon | C_14_H_16_ClN_3_O_2_ | 13.80 | 294.10038 | Check ID | -4.07_X_10^-3^ | 2.08_X_10^-2^ | 0.9927 | 57.2 | * | 100.1 | 115.2 | 10 | 8.7 | 5.5 |
| Triasulfuron | C_14_H_16_ClN_5_O_5_S | 7.56 | 402.06334 | Quan only | -1.74_X_10^-3^ | 5.63_X_10^-3^ | 0.9868 | 56.8 |  | 97.7 | 106.5 | 5 | 17.5 | 16.5 |
| Triazamate | C_13_H_22_N_4_O_3_S | 14.29 | 315.14854 | Check ID | 5.71_X_10^-2^ | 5.20_X_10^-2^ | 0.9882 | 64.1 | * | 95.0 | 98.2 | 10 | 4.6 | 5.3 |
| Triaziflam | C_17_H_24_FN_5_O | 16.46 | 334.20377 | Confirmed | 3.07_X_10^-2^ | 3.98_X_10^-2^ | 0.9890 | 139.7 | * | 157.8 | 152.6 | 10 | 1.7 | 5.1 |
| Triazophos | C_12_H_16_N_3_O_3_PS | 14.81 | 314.07228 | Confirmed | 4.76_X_10^-2^ | 4.23_X_10^-2^ | 0.9896 | 107.2 | * | 125.3 | 125.3 | 10 | 1.5 | 4.3 |
| Triazoxide | C_1_0H_6_ClN_5_O | 10.94 | 248.03336 | Check ID | 6.21_X_10^-3^ | 2.17_X_10^-2^ | 0.9863 | 65.6 |  | 83.9 | 85.2 | 5 | 4.9 | 5.3 |
| Tribenuron-methyl | C_15_H_17_N_5_O_6_S | 10.55 | 396.09723 | Quan only | -2.81_X_10^-3^ | 4.91_X_10^-3^ | 0.9917 | 60.4 |  | 111.5 | 115.9 | 5 | 11.3 | 12.4 |
| Tribufos (def) | C_12_H_27_OPS_3_ | 22.19 | 315.10344 | Confirmed | 6.47_X_10^-2^ | 5.89_X_10^-2^ | 0.9885 | 81.8 | * | 92.3 | 90.8 | 10 | 9.8 | 7.4 |
| Tricyclazole | C_9_H_7_N_3_S | 7.08 | 190.04334 | Check ID | 5.99_X_10^-3^ | 3.35_X_10^-2^ | 0.9875 | 35.2 |  | 51.7 | 55.8 | 5 | 8.9 | 7.6 |
| Trifloxystrobin | C_2_0H_19_F_3_N_2_O_4_ | 19.28 | 409.13697 | Confirmed | 2.16_X_10^-2^ | 3.38_X_10^-2^ | 0.9918 | 93.3 | * | 106.3 | 101.8 | 10 | 1.8 | 4.1 |
| Triflumizole | C_15_H_15_ClF_3_N_3_O | 19.60 | 346.09285 | Check ID | 1.82_X_10^-2^ | 3.67_X_10^-2^ | 0.9924 | 100.7 |  | 108.5 | 103.9 | 5 | 4.7 | 4.1 |
| Triflumuron | C_15_H_1_0ClF_3_N_2_O_3_ | 18.38 | 359.04048 | Check ID | 1.07_X_10^-3^ | 3.66_X_10^-3^ | 0.9902 | 40.6 | * | 91.7 | 101.1 | 10 | 5.8 | 7.4 |
| Tris(2-chloroethyl) Phosphate | C_6_H_12_Cl_3_O_4_P | 7.72 | 284.96115 | Confirmed | -4.24_X_10^-4^ | 7.98_X_10^-3^ | 0.9890 | 51.2 |  | 92.3 | 97.9 | 5 | 15.1 | 9.4 |
| Tris  (o-cresyl)phosphate | C_21_H_21_O_4_P | 21.13 | 369.12502 | Confirmed | 1.56_X_10^-2^ | 2.13_X_10^-2^ | 0.9884 | 49.0 | * | 67.9 | 69.4 | 10 | 4.2 | 16.4 |
| Triticonazole | C_17_H_2_0ClN_3_O | 14.92 | 318.13677 | Check ID | 1.26_X_10^-2^ | 1.26_X_10^-2^ | 0.9909 | 77.1 | * | 112.4 | 113.2 | 10 | 4.7 | 6.3 |
| Uniconazole | C_15_H_18_ClN_3_O | 15.82 | 292.12112 | Check ID | 4.42_X_10^-3^ | 1.27_X_10^-2^ | 0.9917 | 85.3 |  | 107.5 | 118.3 | 5 | 3.9 | 12.1 |
| Zoxamide | C_14_H_16_Cl_3_NO_2_ | 17.03 | 336.03194 | Confirmed | 8.91_X_10^-3^ | 1.28_X_10^-2^ | 0.9898 | 68.5 |  | 95.4 | 94.9 | 5 | 12.1 | 7.9 |


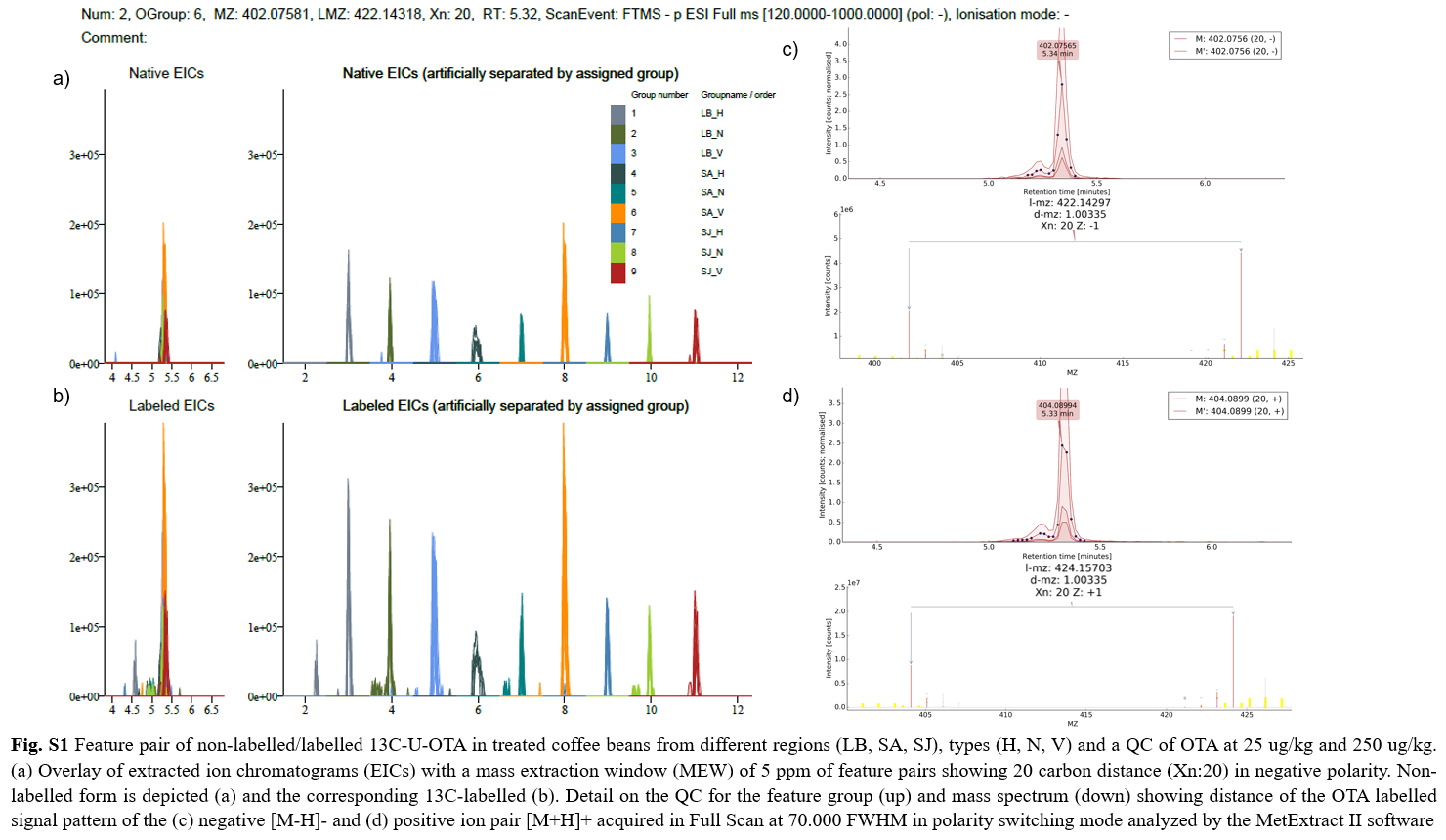
Figure S1

Figure S2


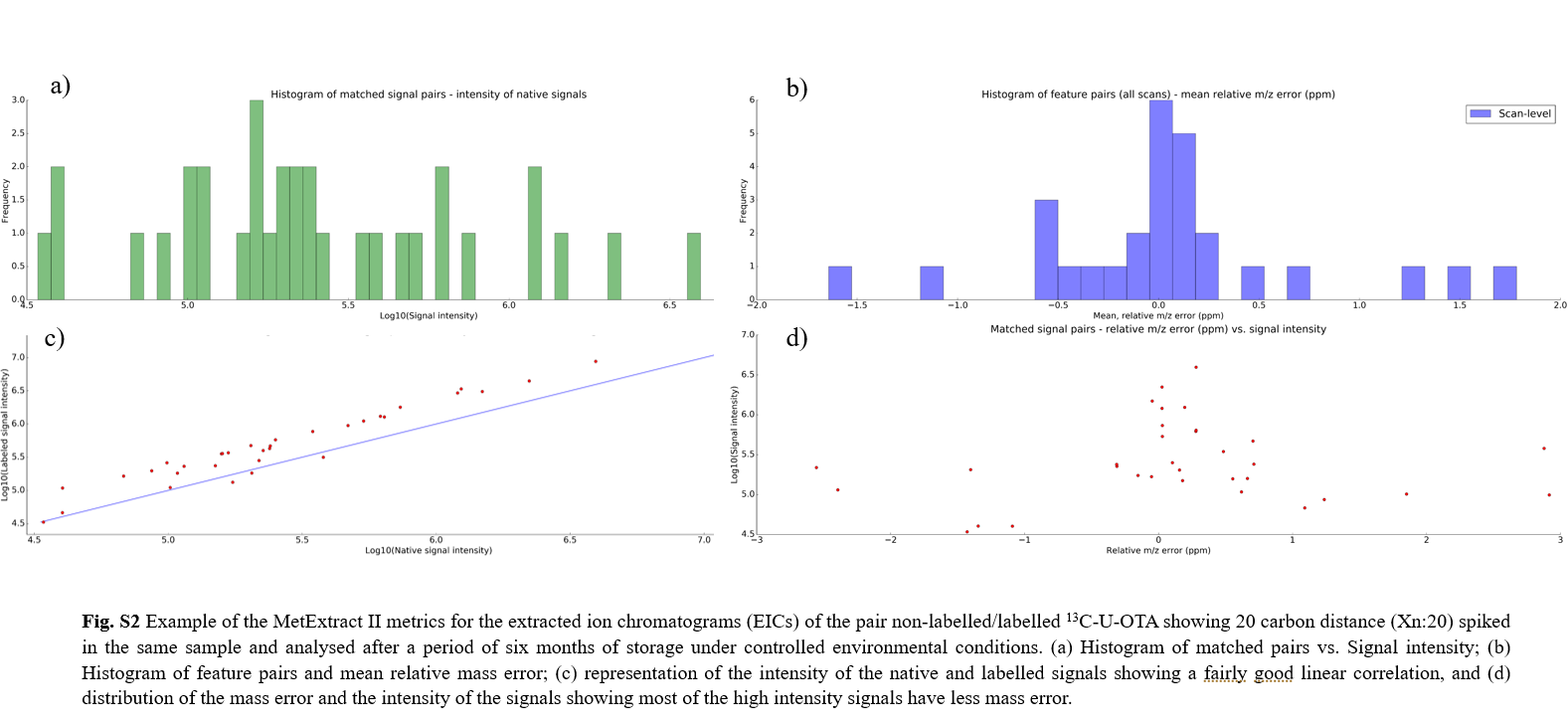


Figure S3


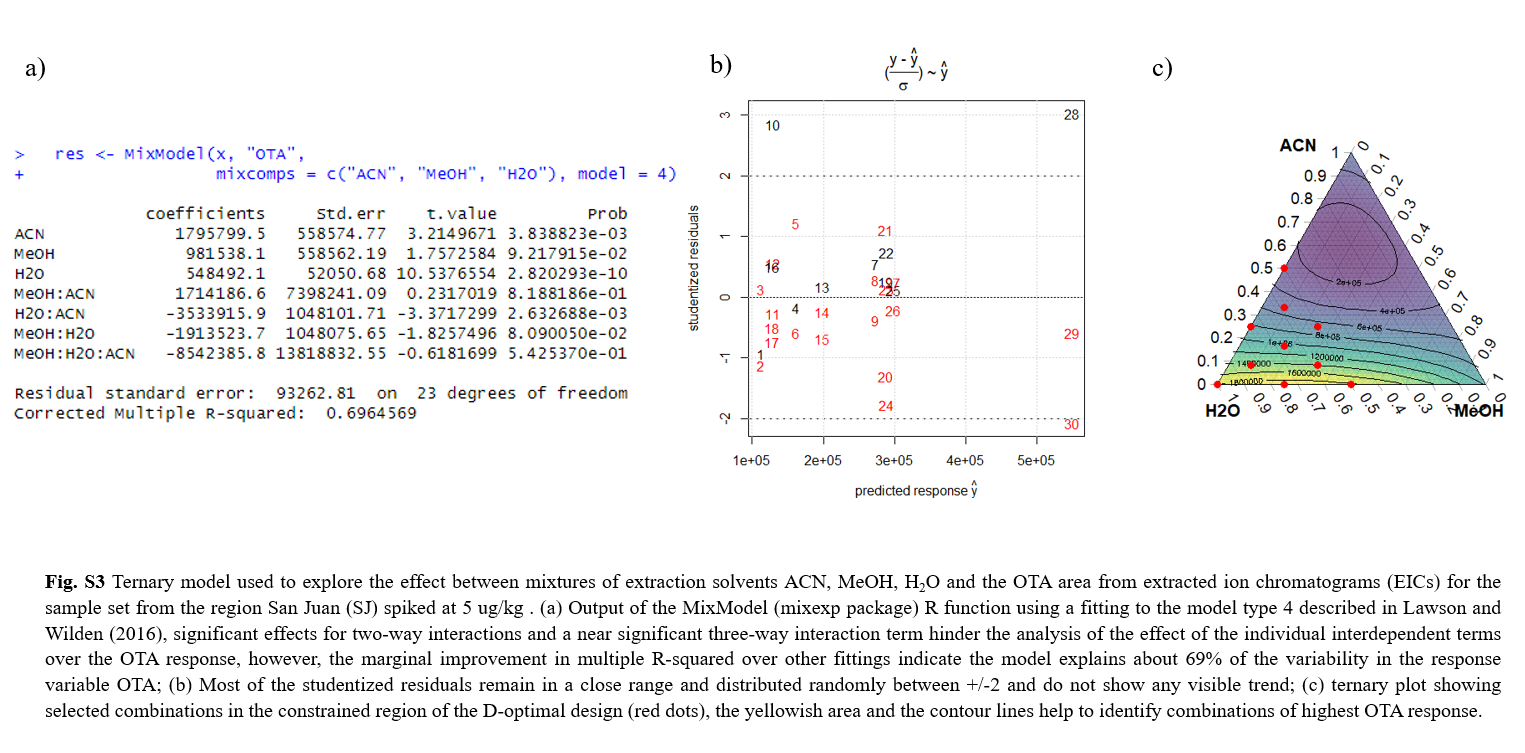


Figure S4


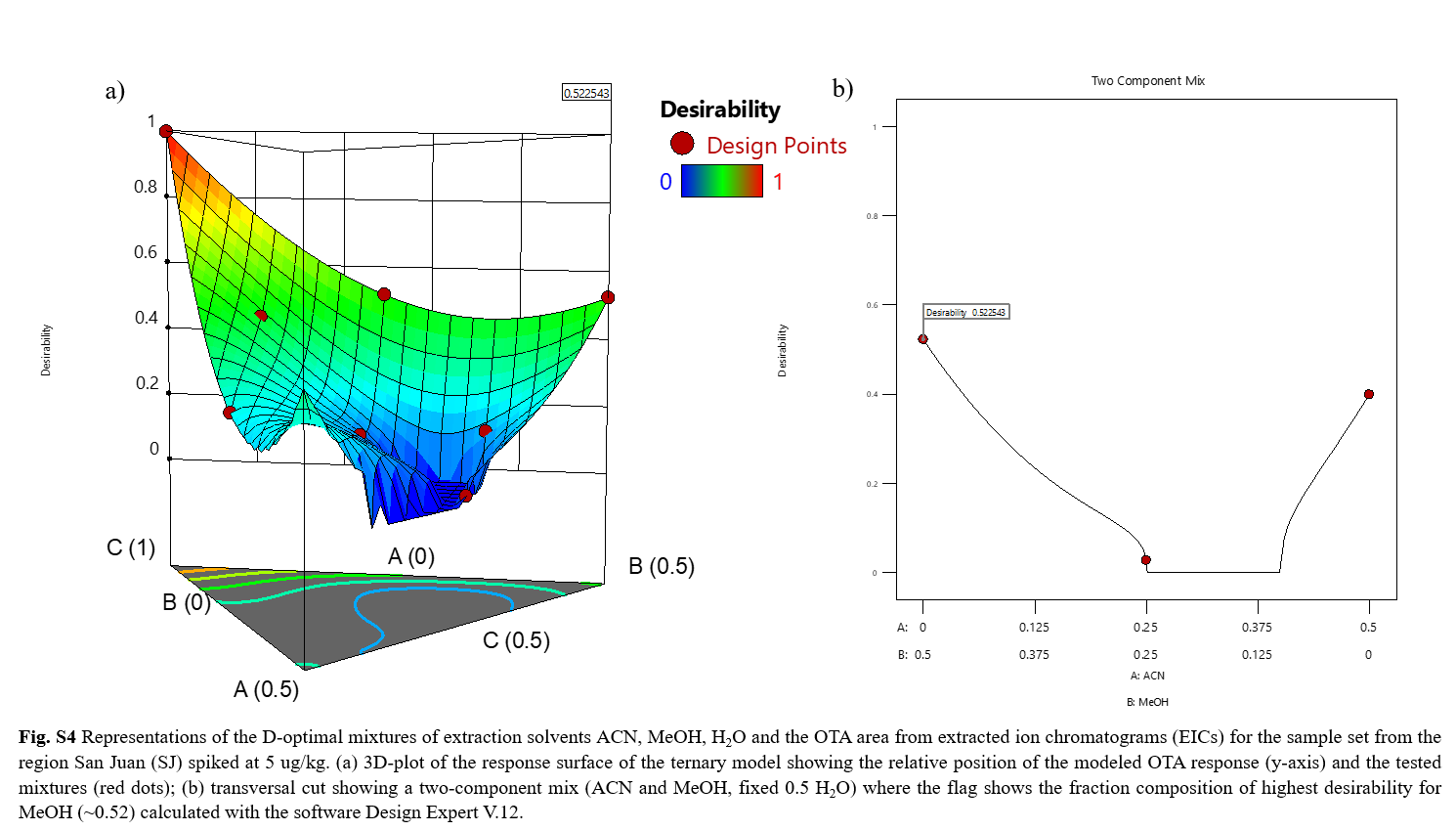


Figure S5


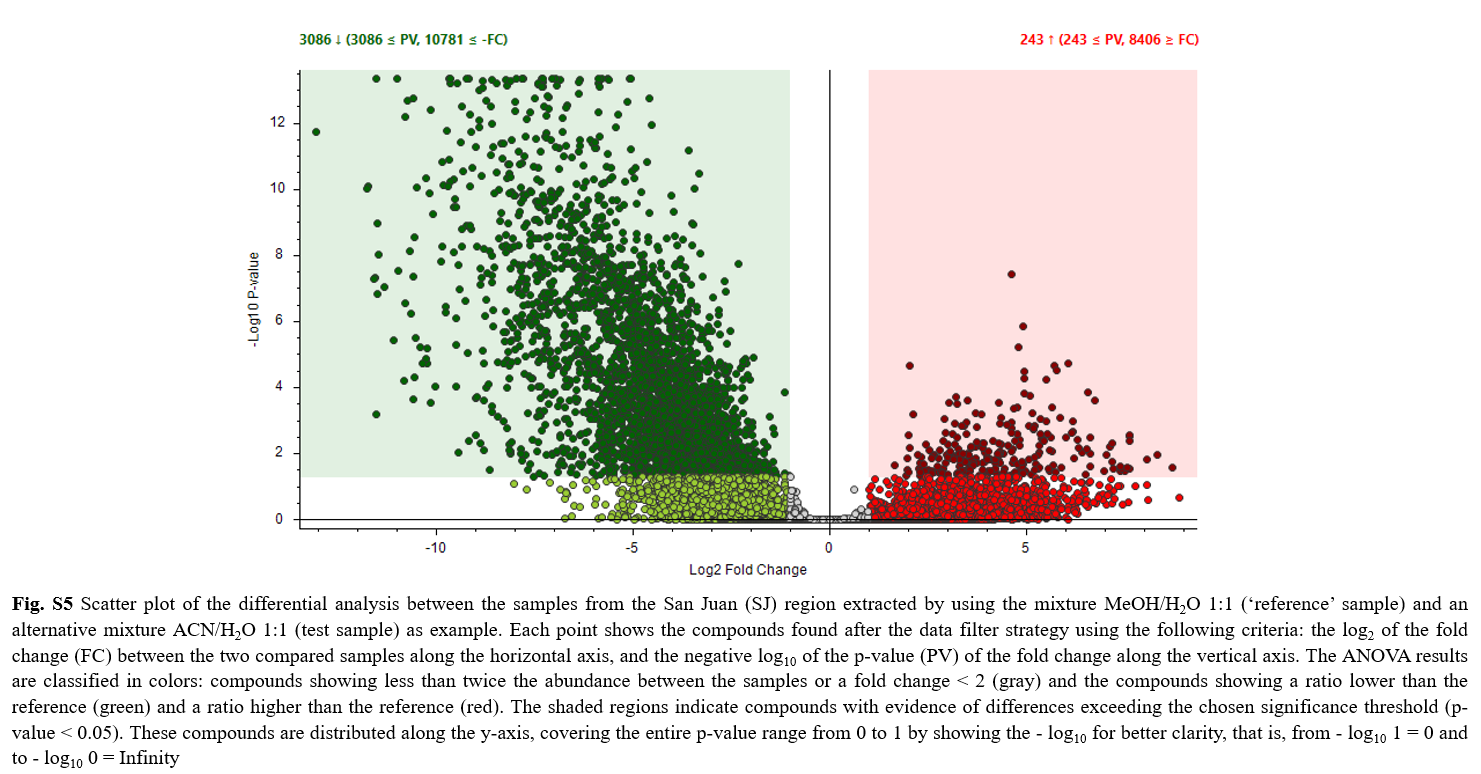


Figure S6


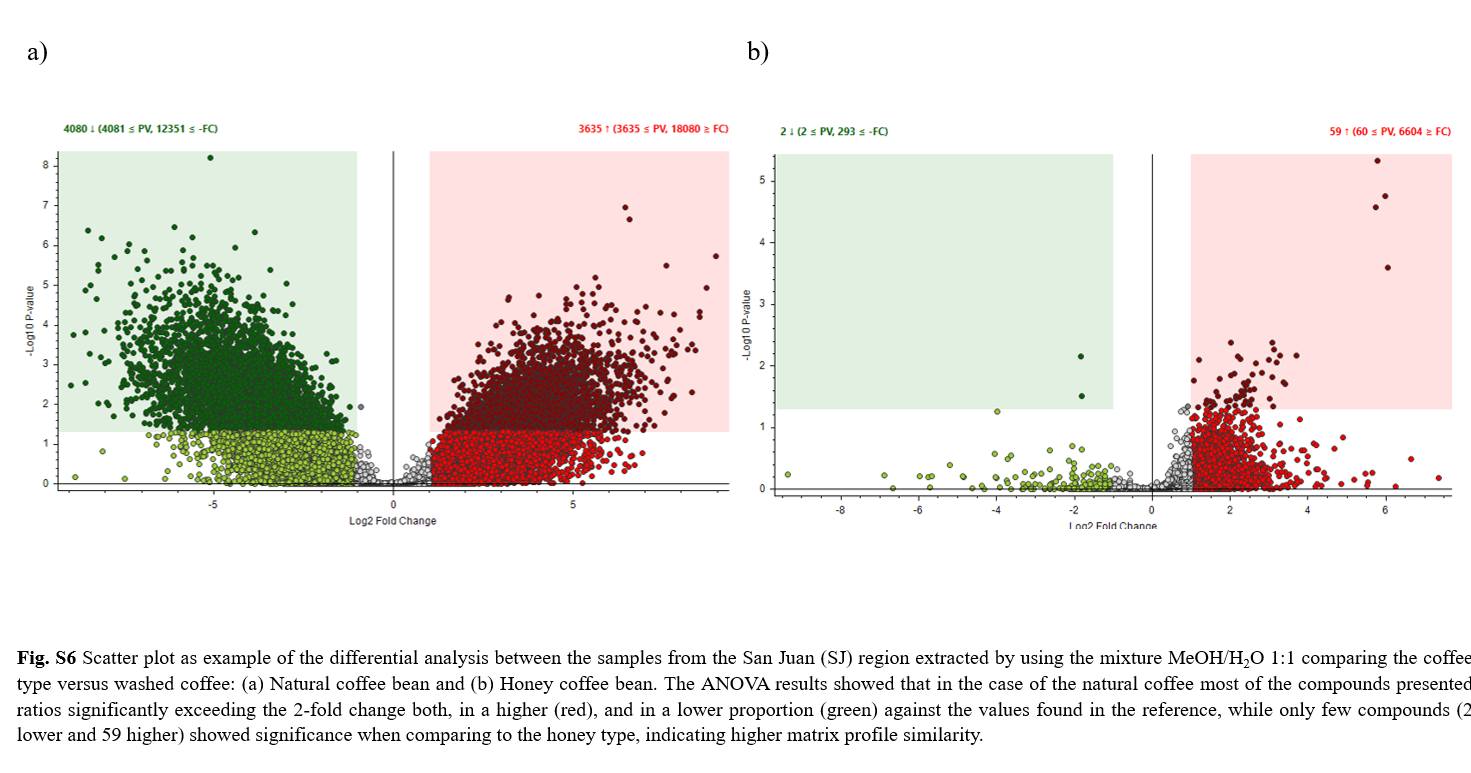


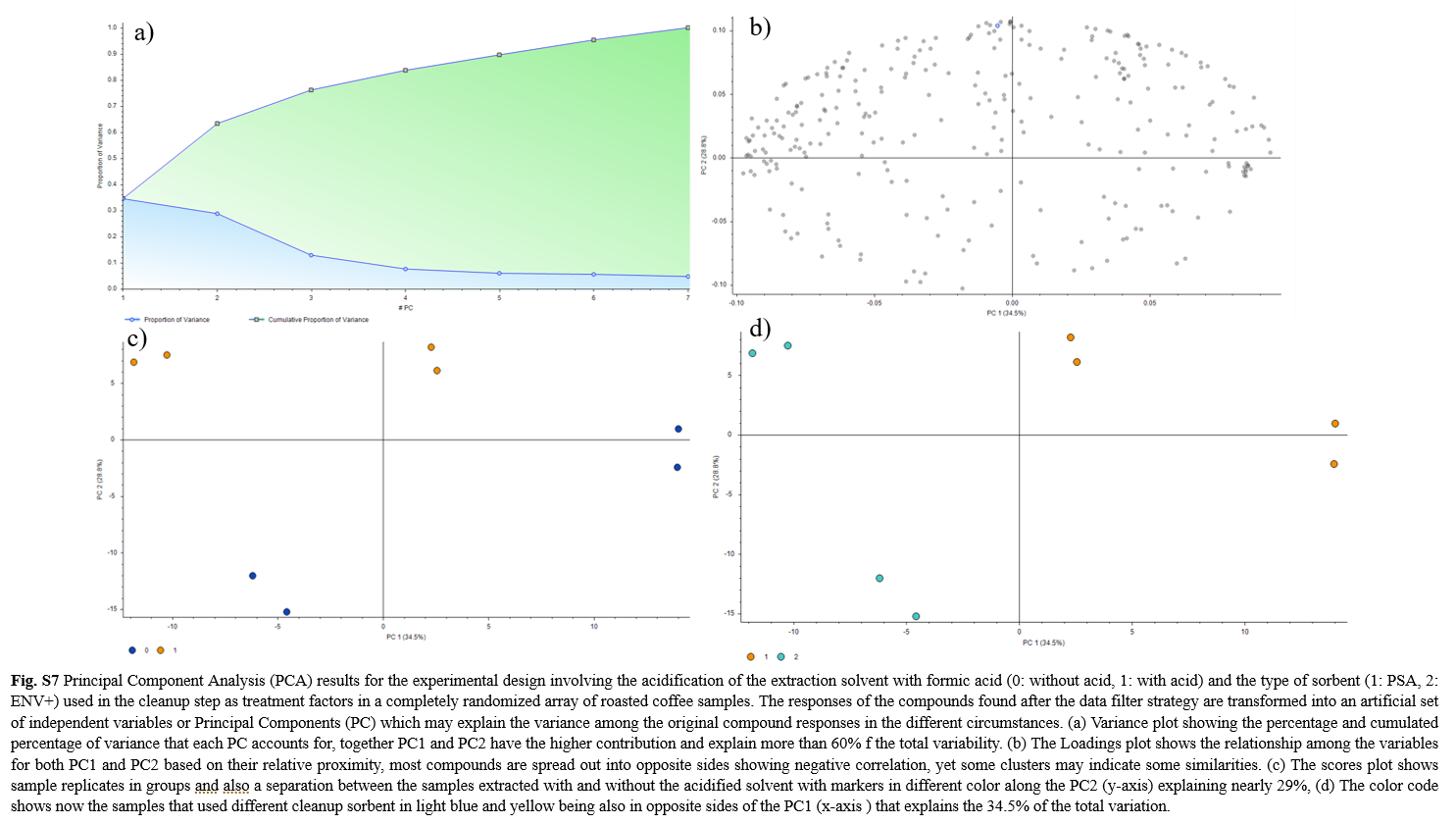
Figure S7

Figure S8


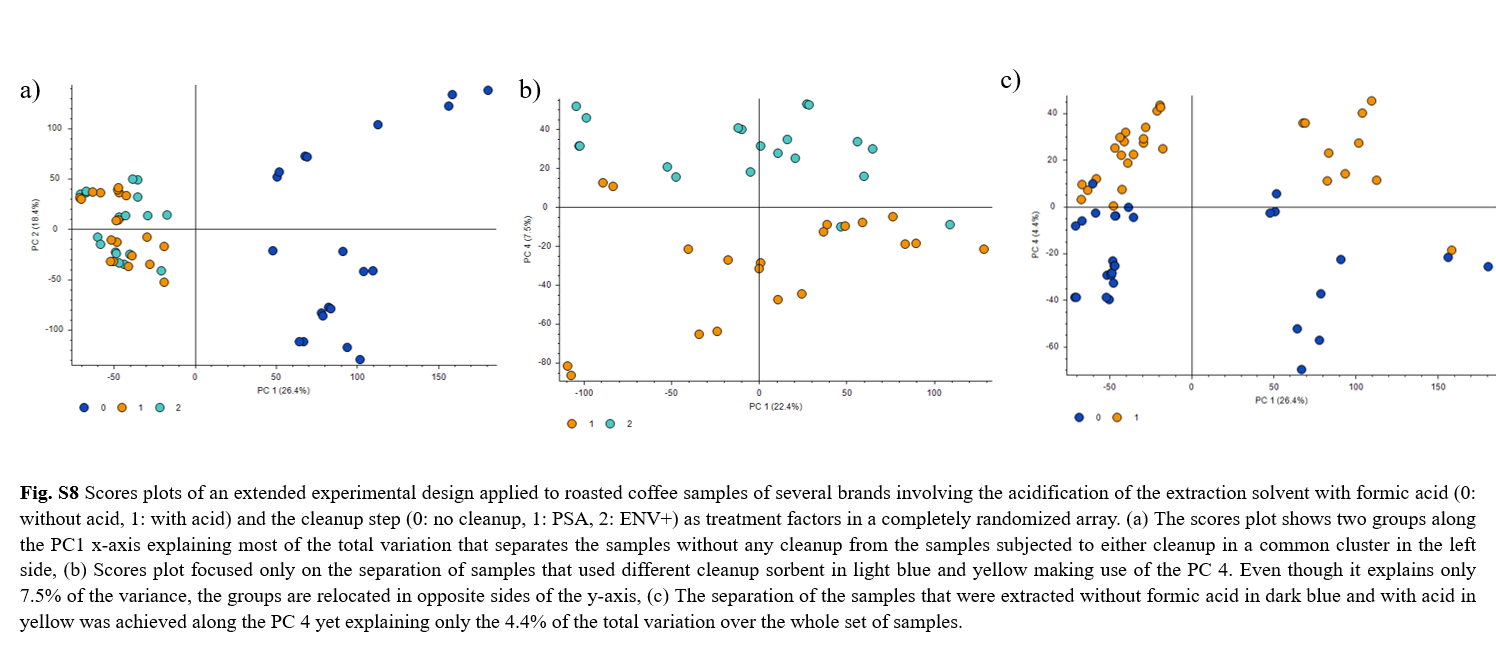


Figure S9


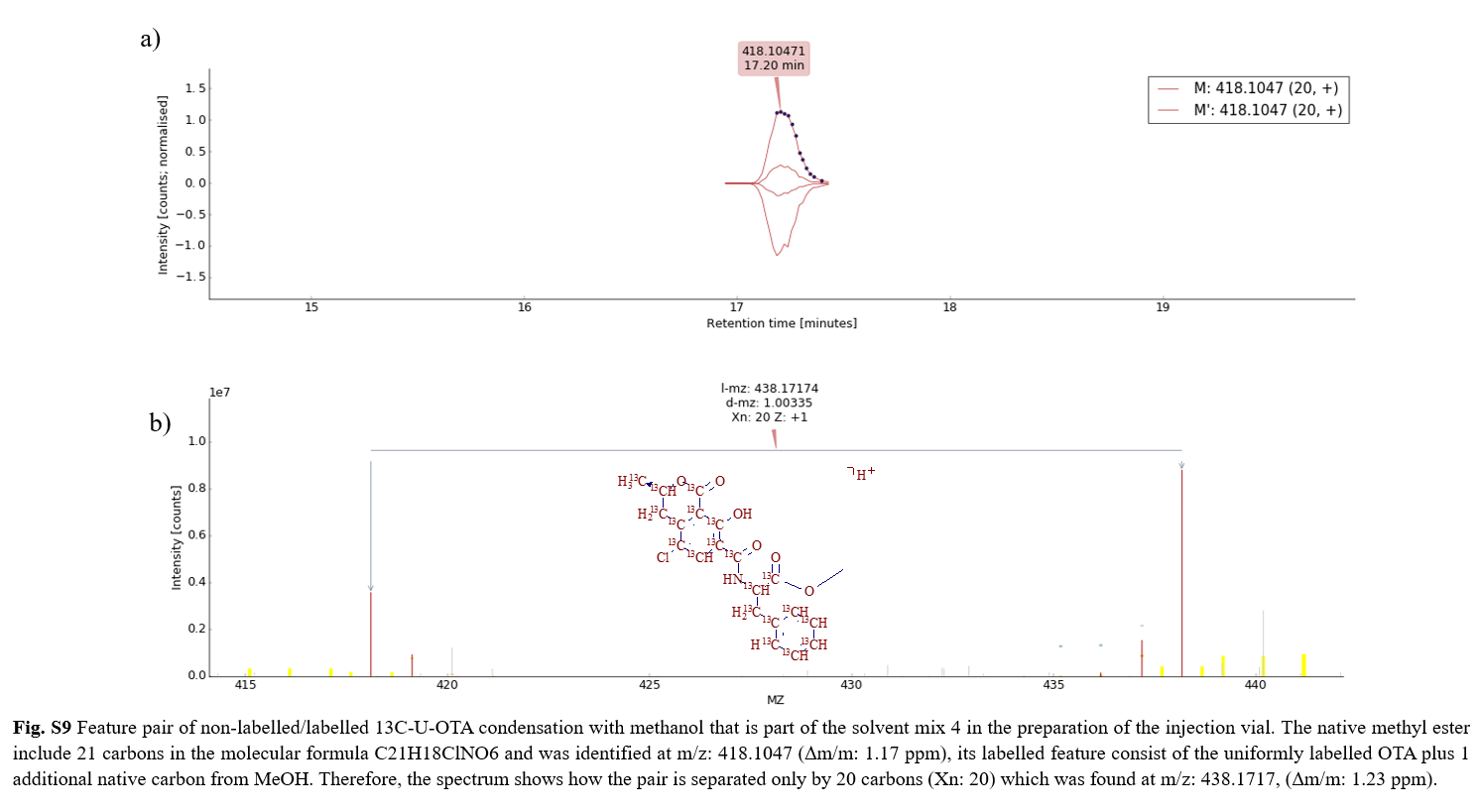


Figure S10


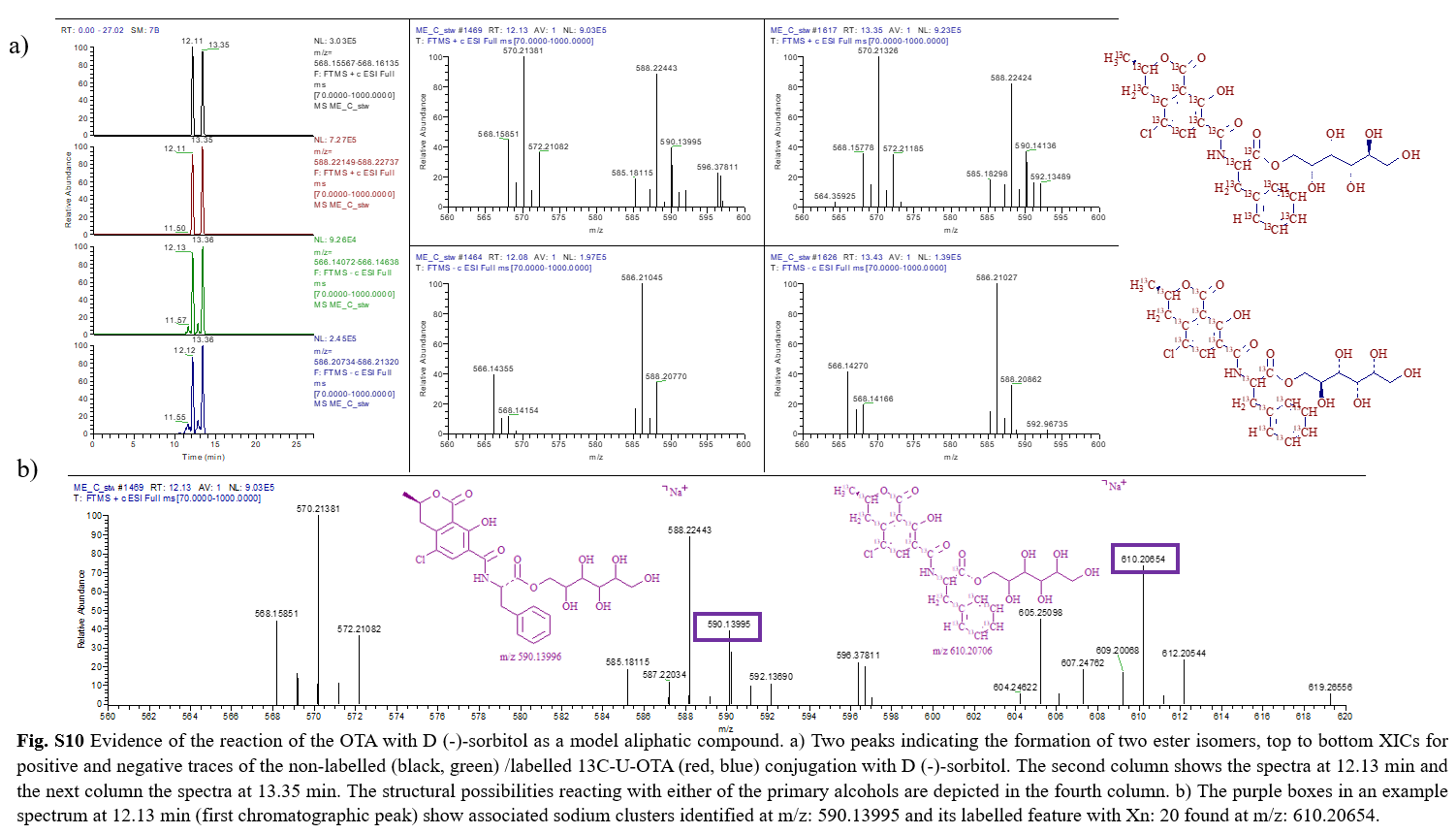


Figure S11


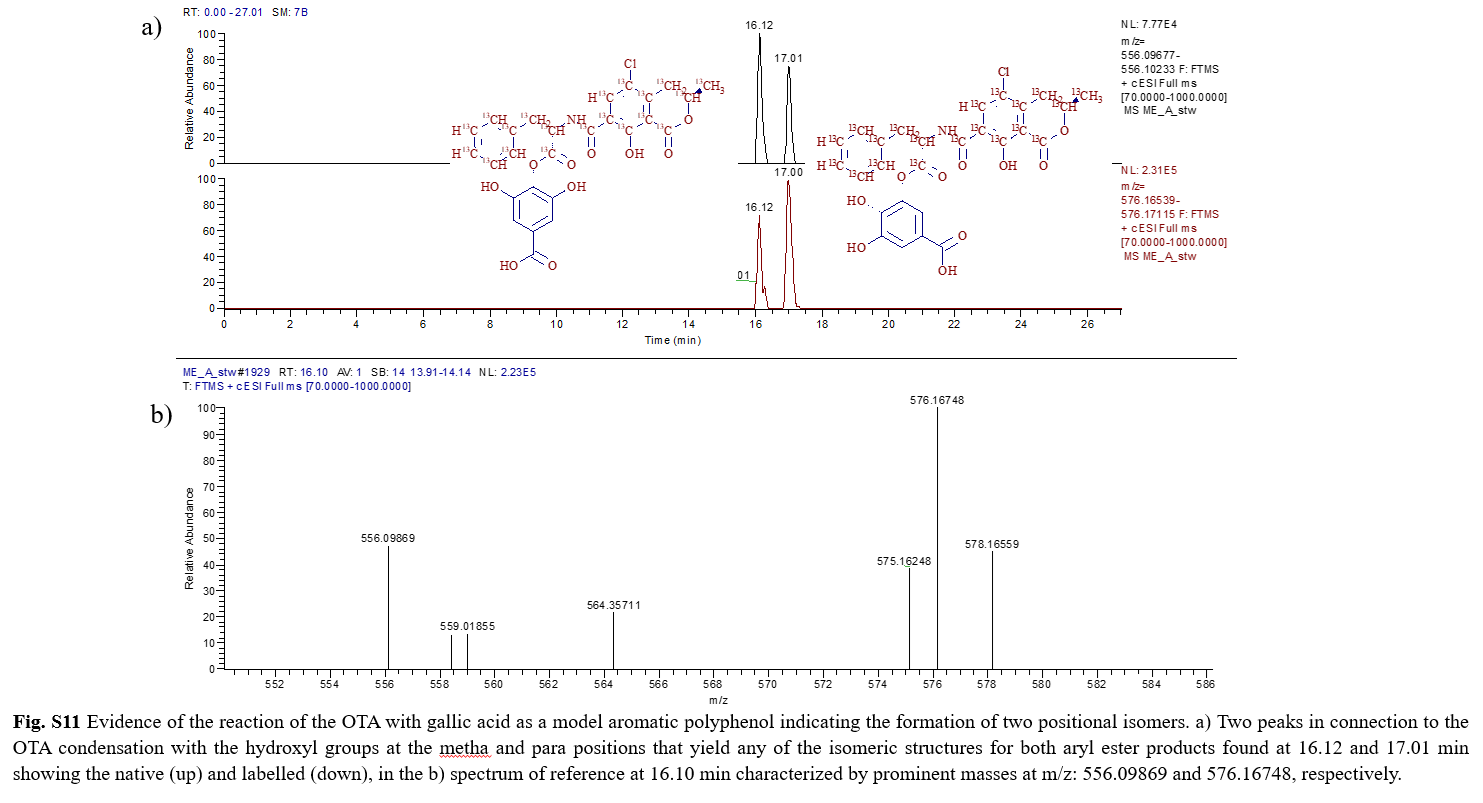


Figure S12


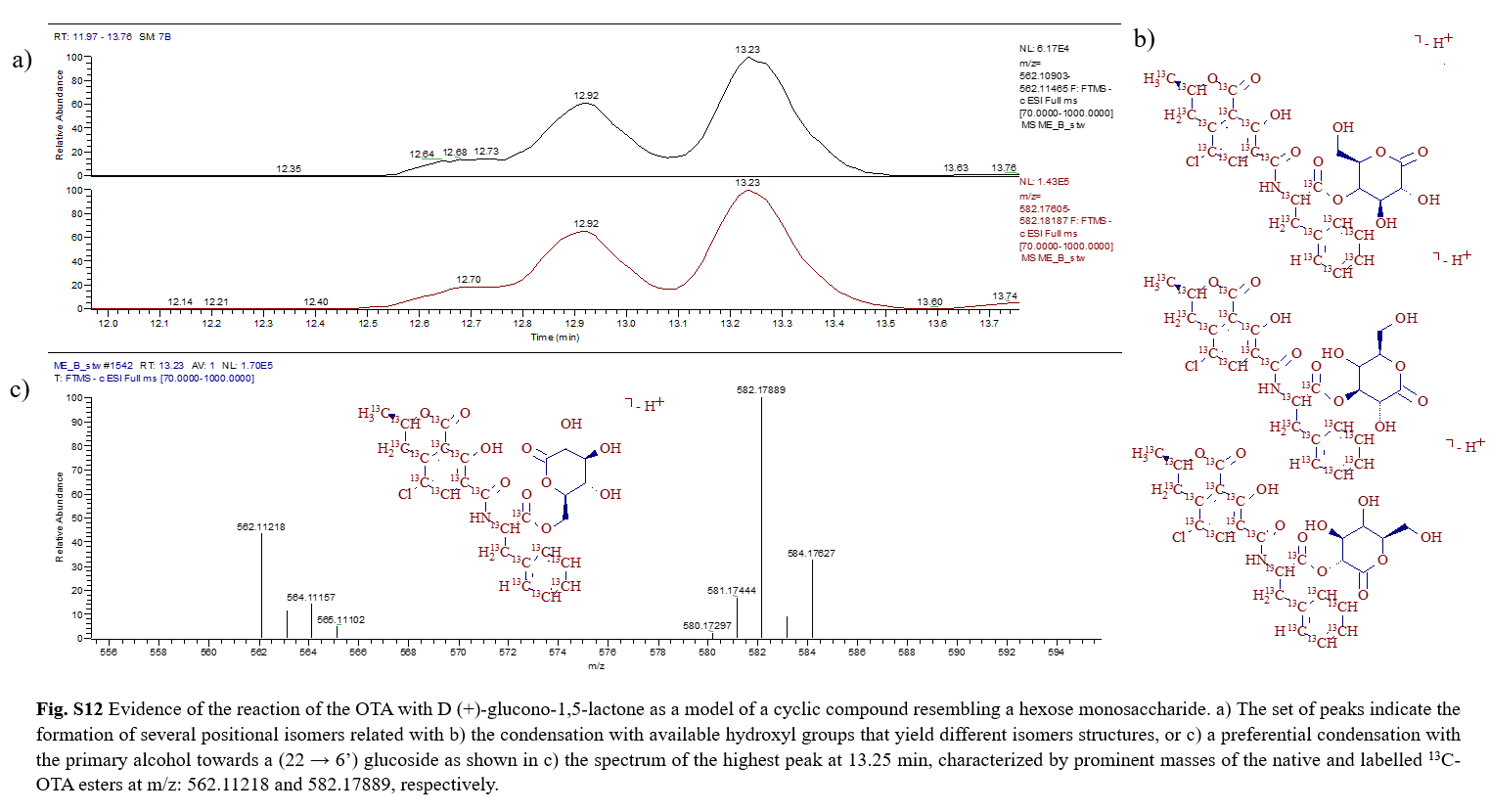


Figure S13


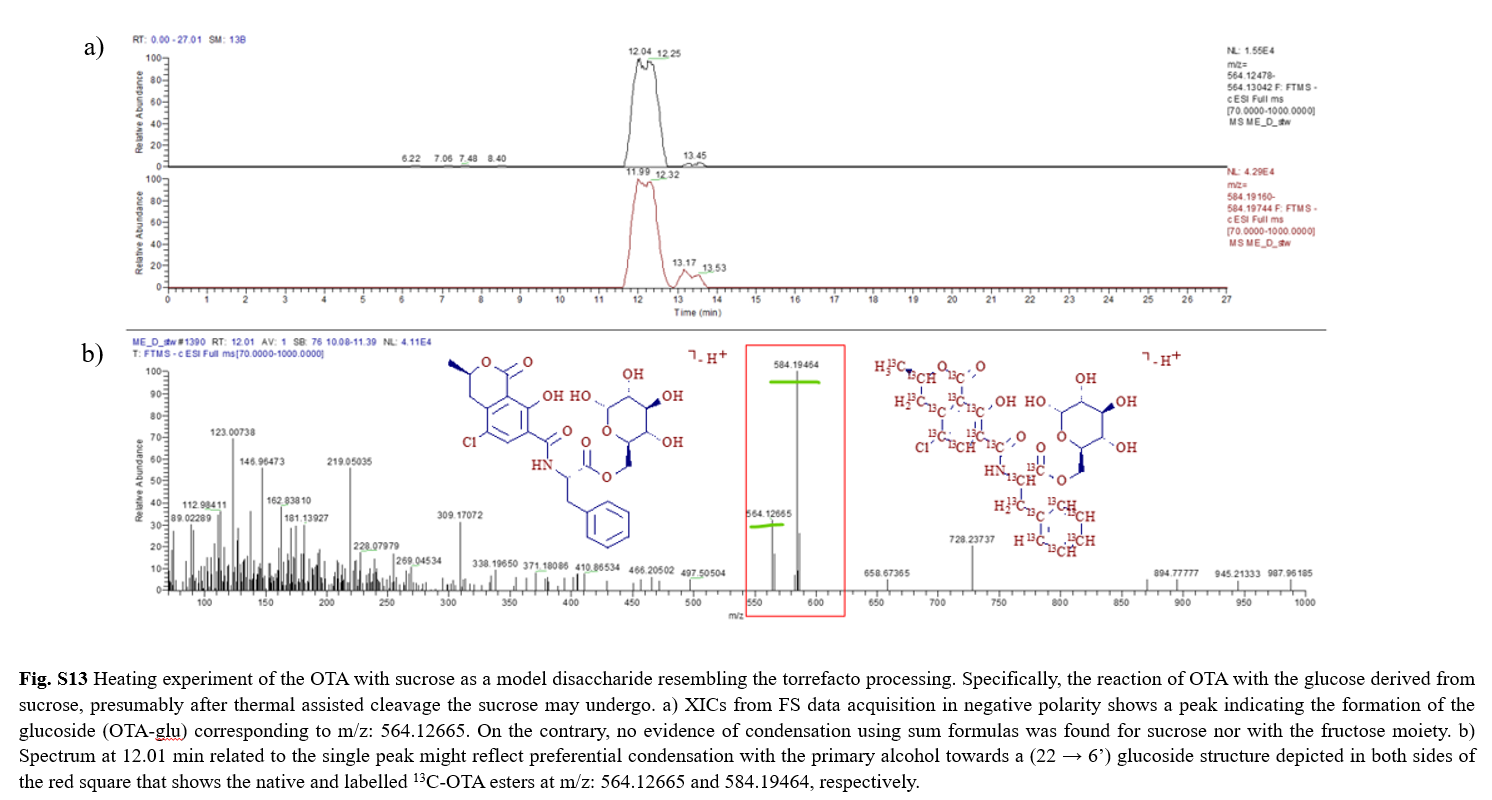


Figure S14


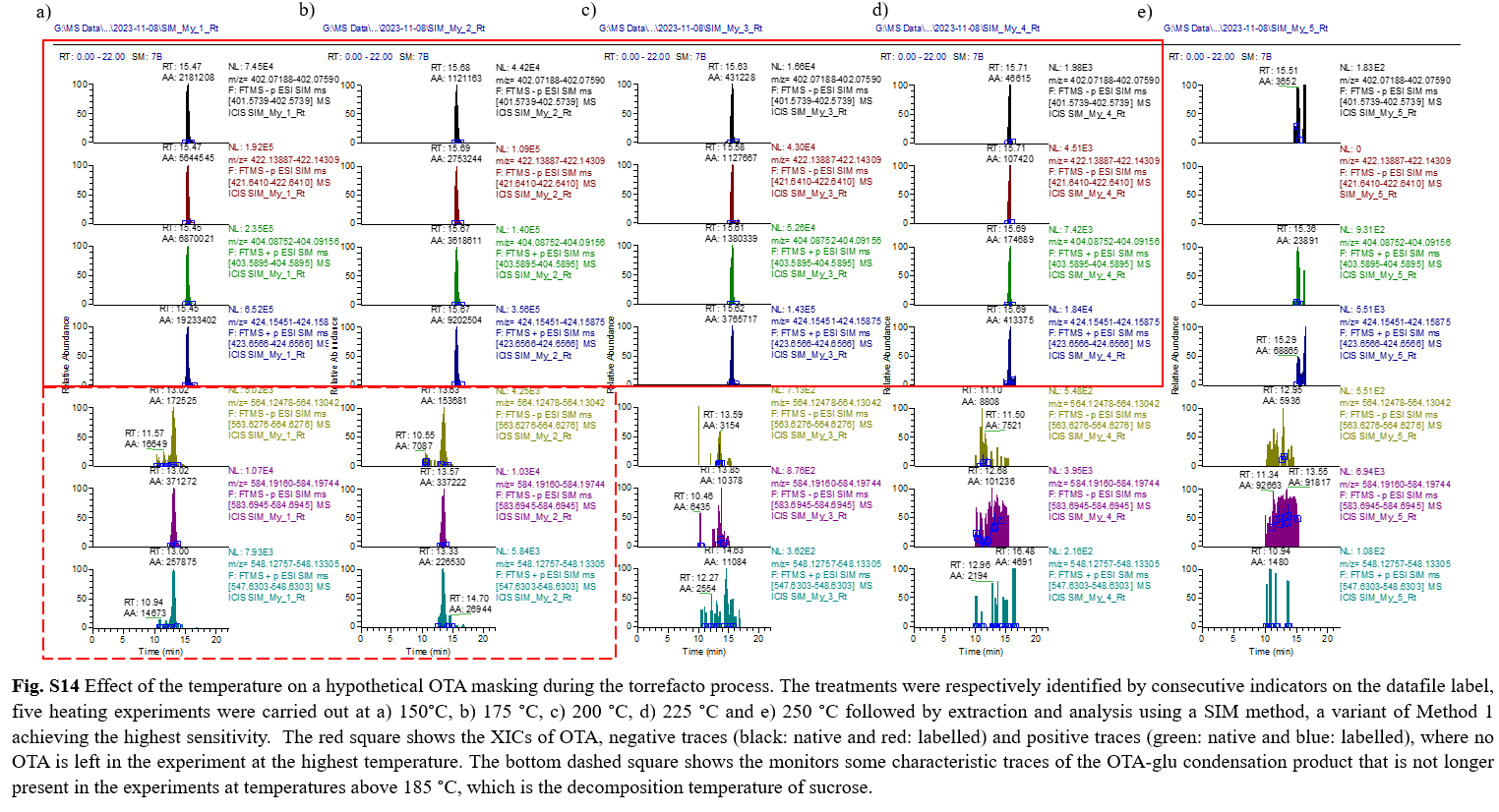


Figure S15


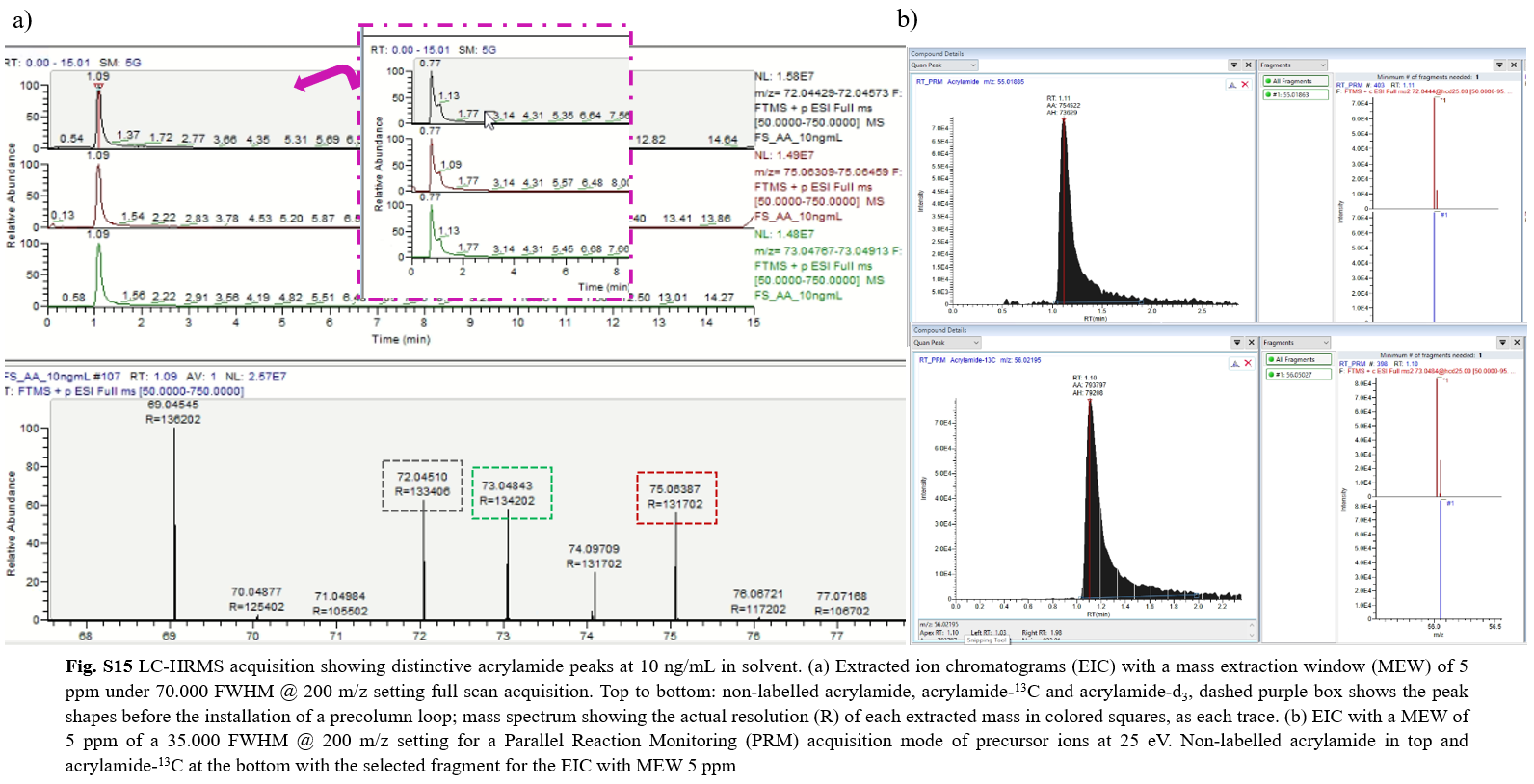


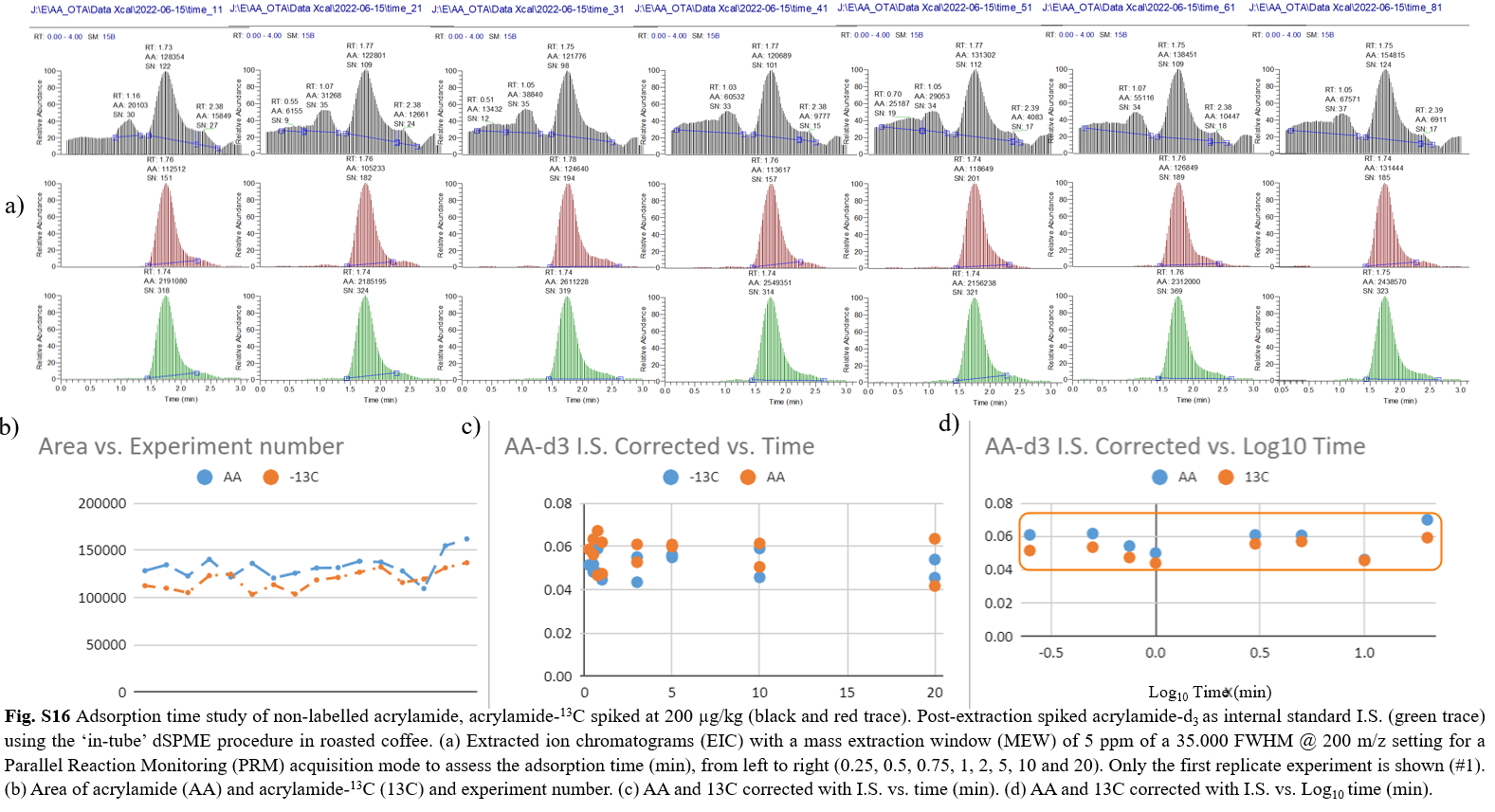
Figure S16


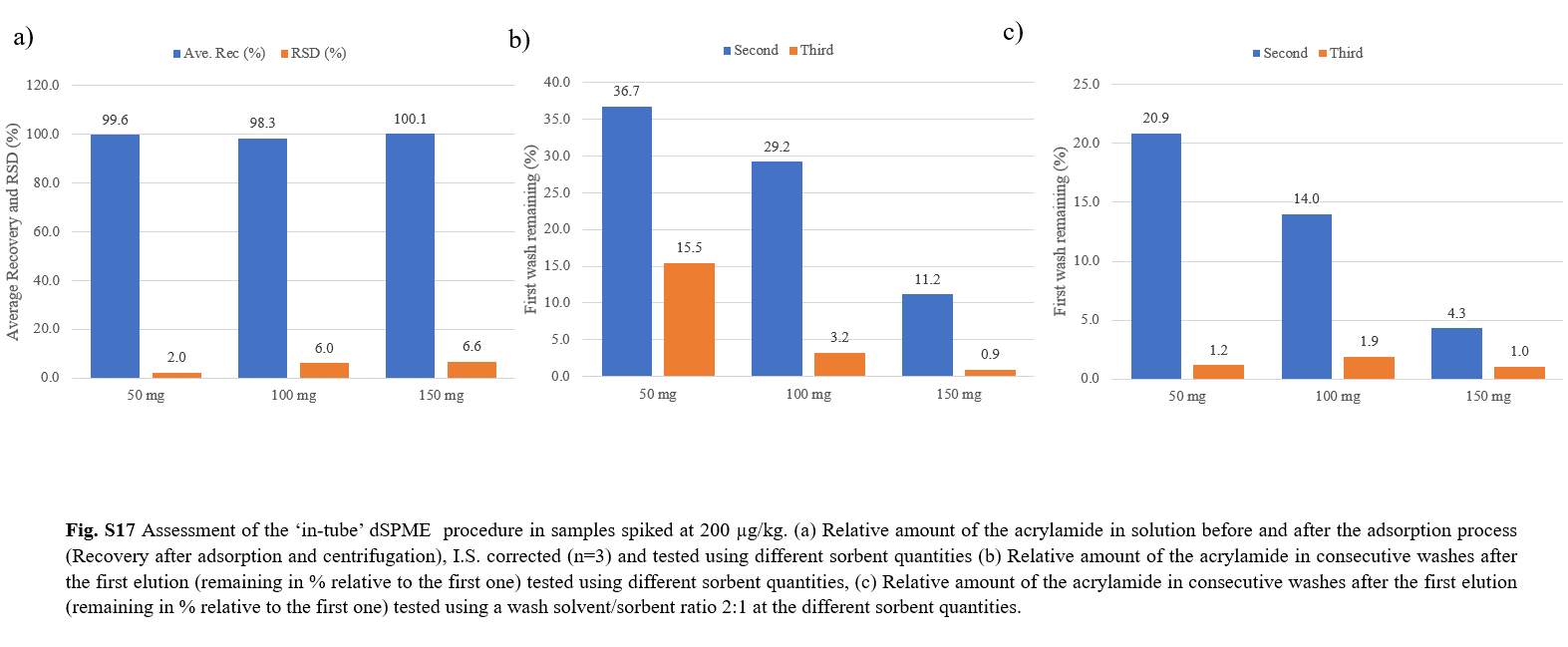
Figure S17

Note: A serial washing was also evaluated and indicated that the higher the elution volume the less AA remains for another wash. Note that the wash volume increases as the sorbent quantity to keep the same sorbent-liquid ratio. Hence, the higher the volume the lower the final concentration due to the dilution factor. In addition, the same experiment was performed but using a sorbent – wash ratio 2:1 to check for any improvement but the same behavior was found. Therefore, the ratio 1:1 was kept, and no sequential washing was considered but only the first elution. Due to practical reasons, the quantities of sorbent were tweaked to fit the dimensions of the filter and allow room enough for vortex matching the values in Figure 1. It is worth to notice that, just like the SPE methods that use a cleaning step (usually with a multimode C18 cartridge) prior the retention of the AA onto the ENV+, the adsorption in this study is not hindered thanks to the use of EtOAc as a defatting agent of the aqueous phase that undergoes the dSPME procedure.

Figure S18


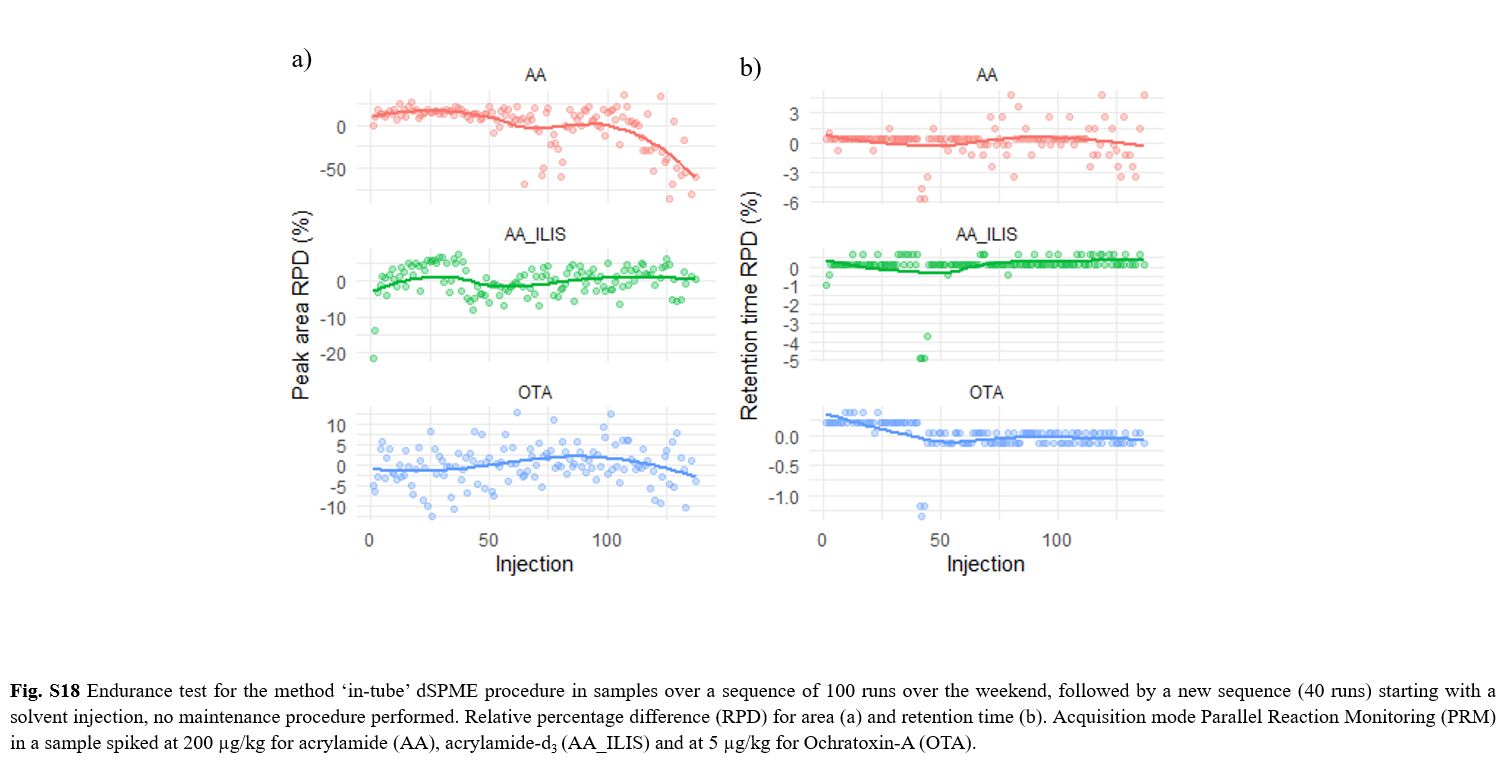


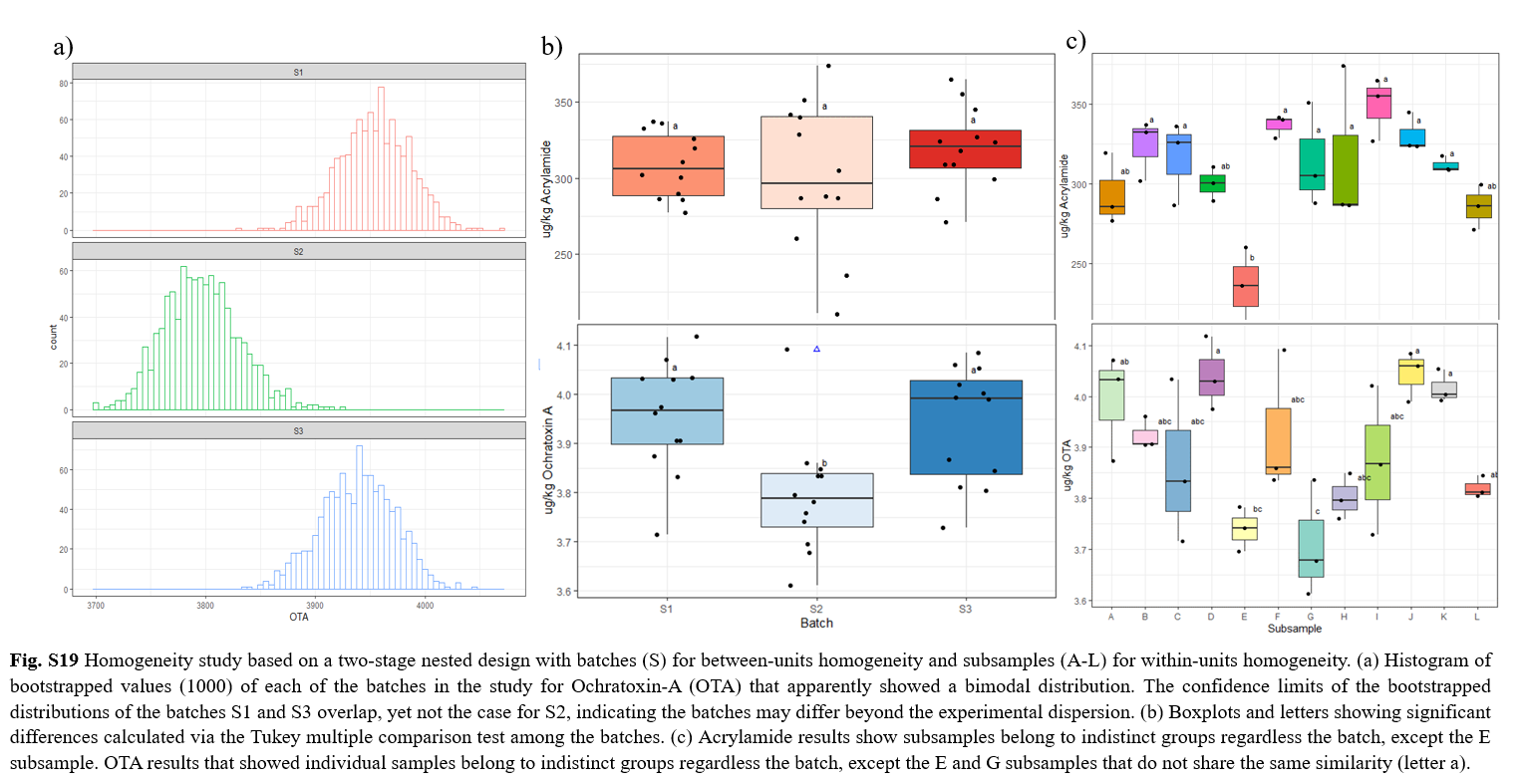
Figure S19


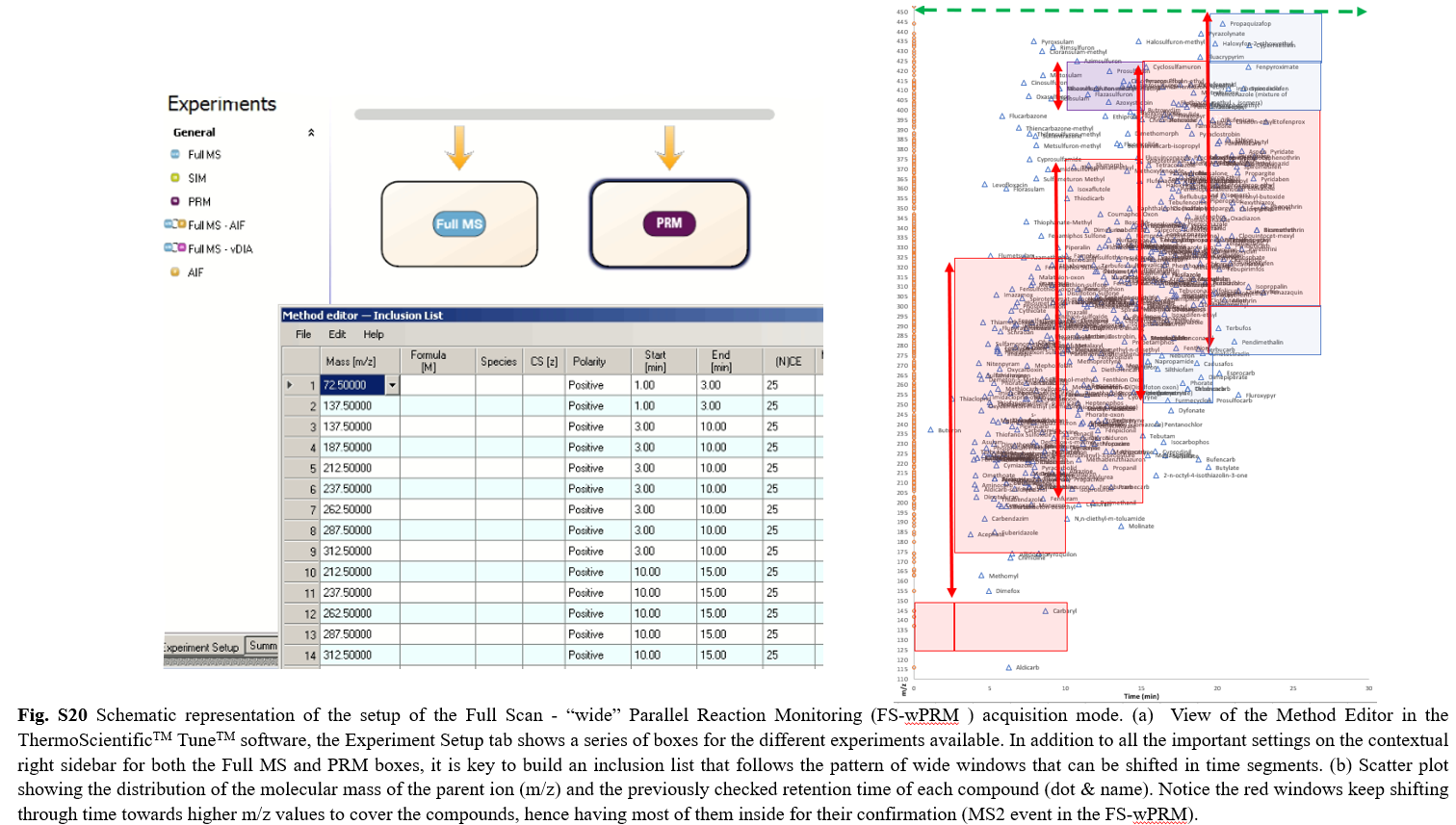
Figure S20


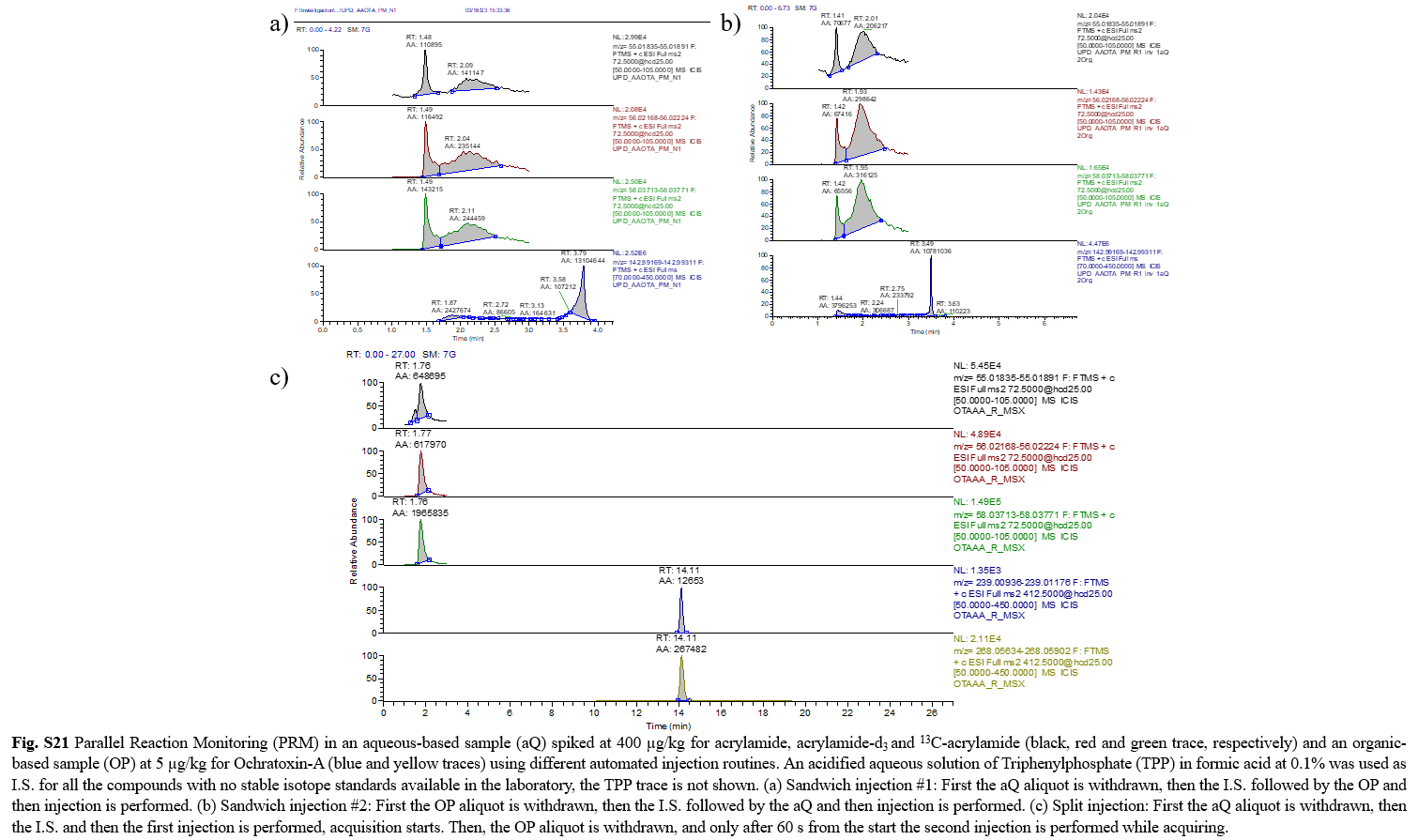
Figure S21


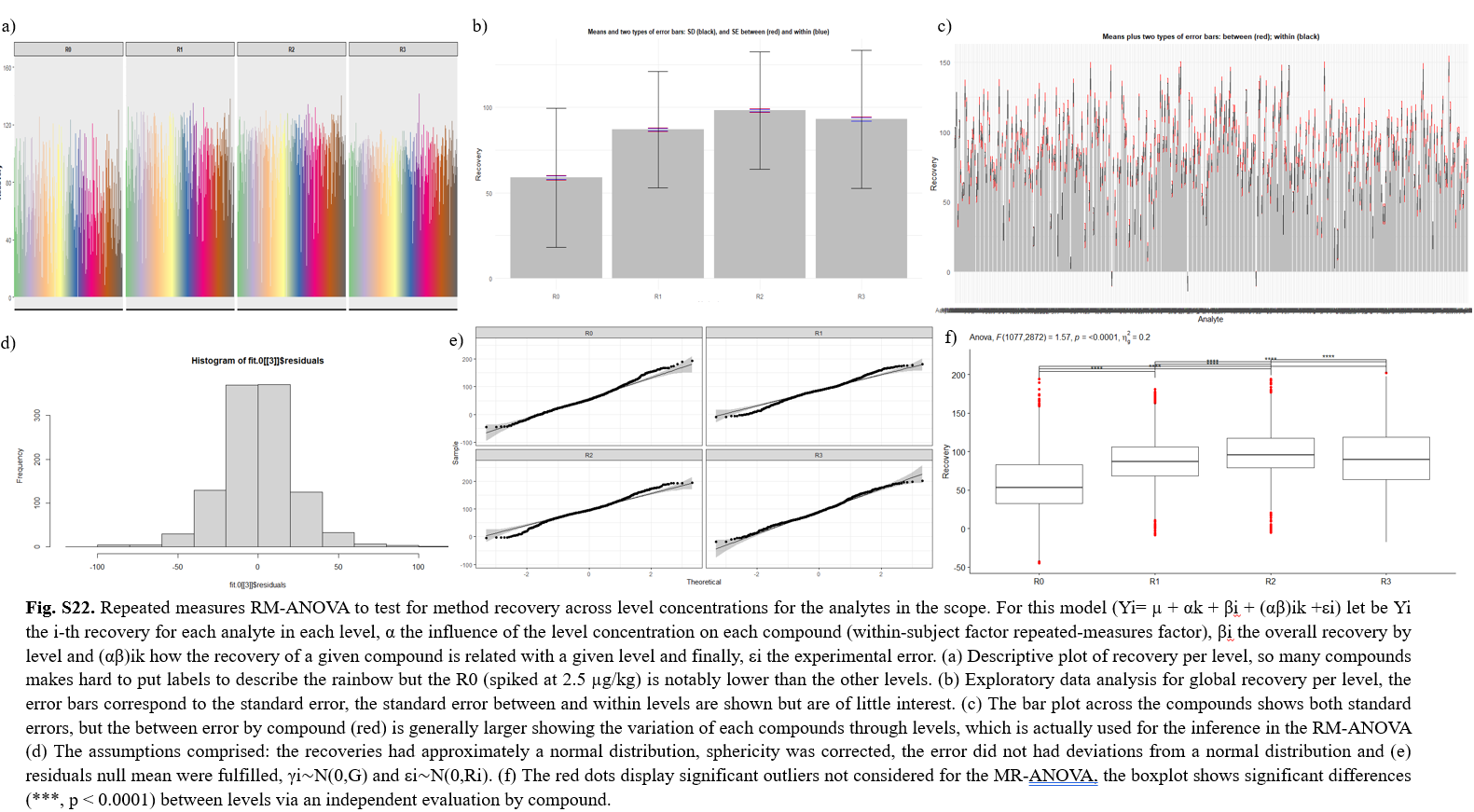
Figure S22


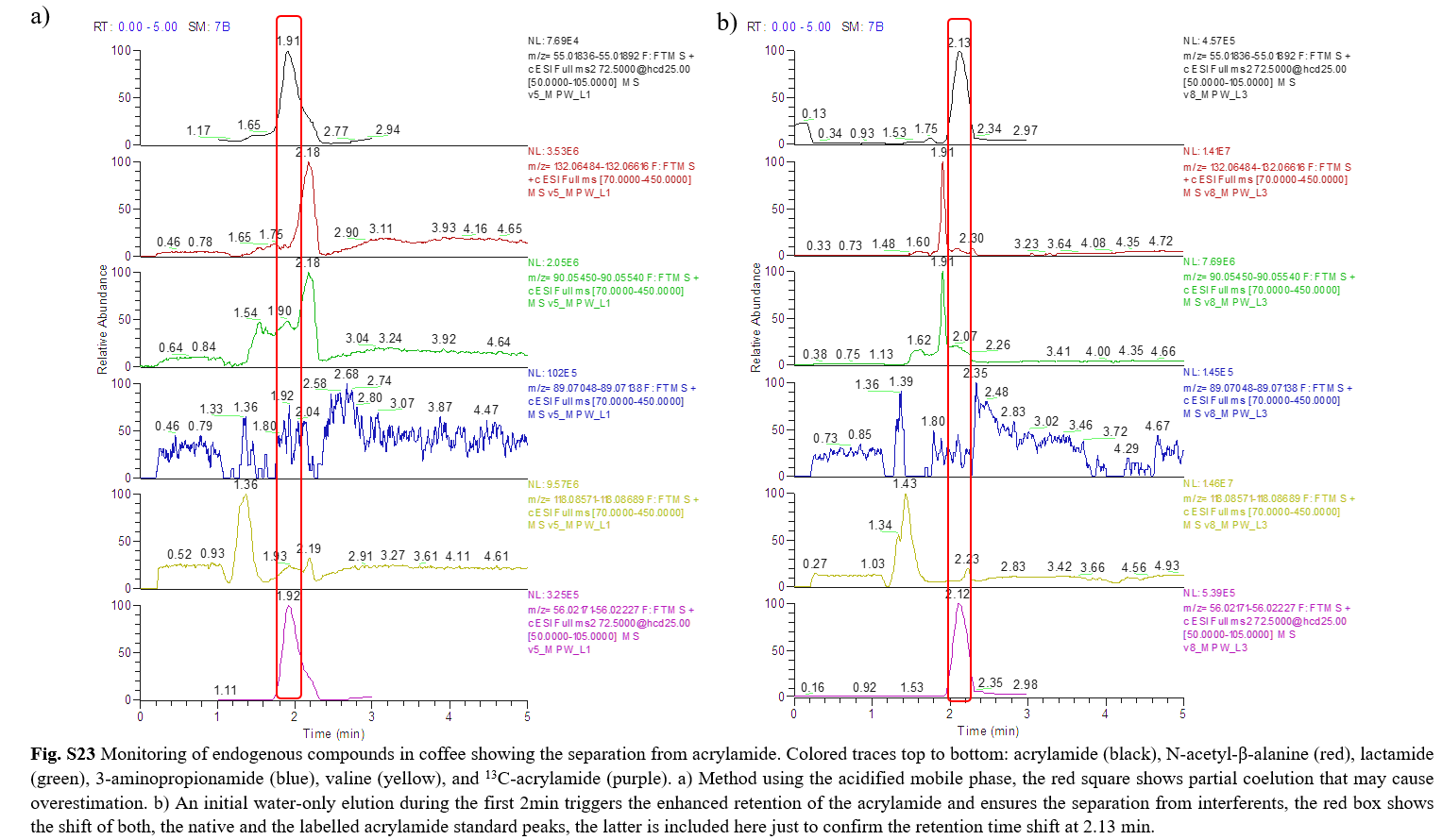
Figure S23


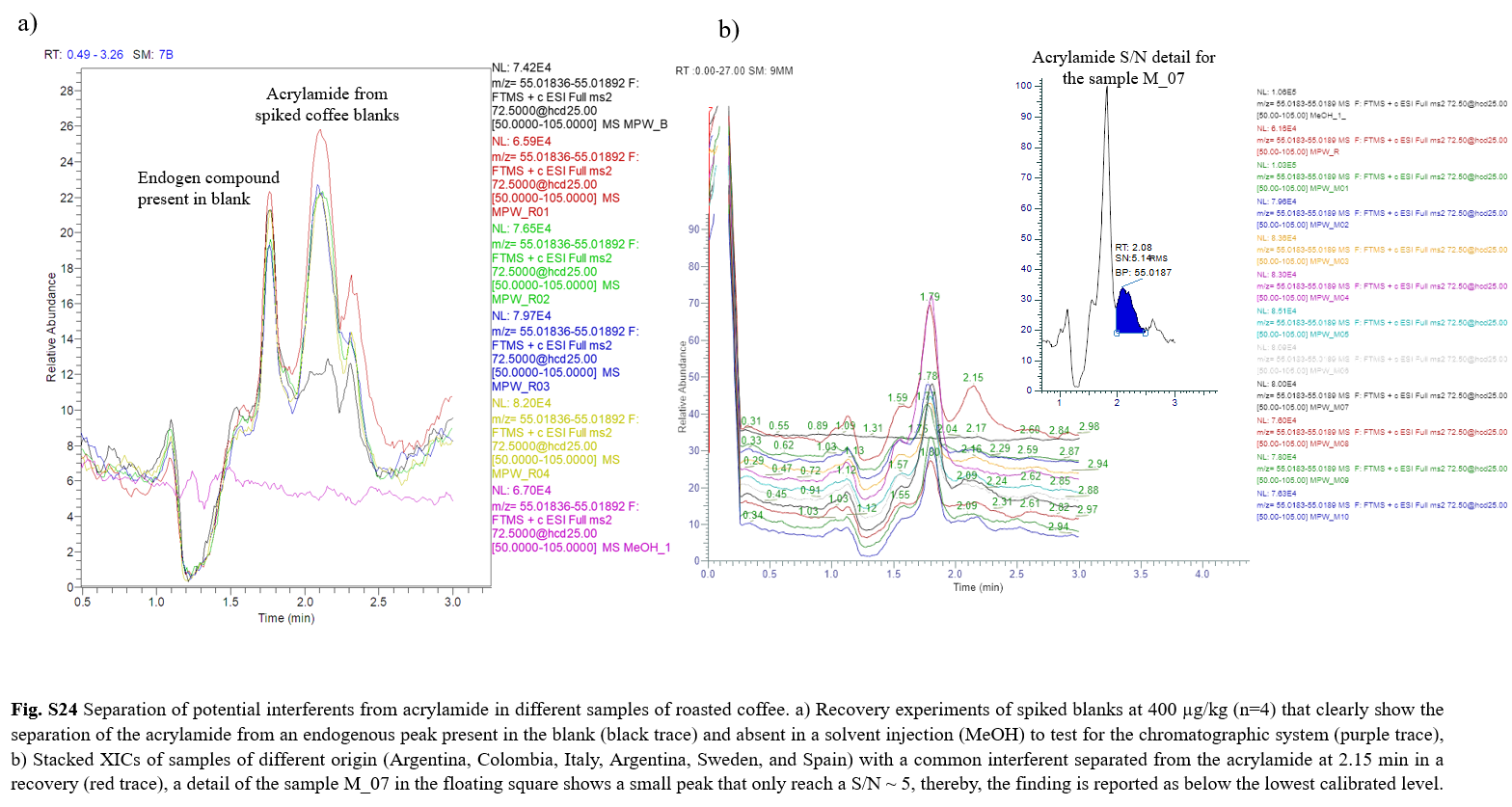
Figure S24


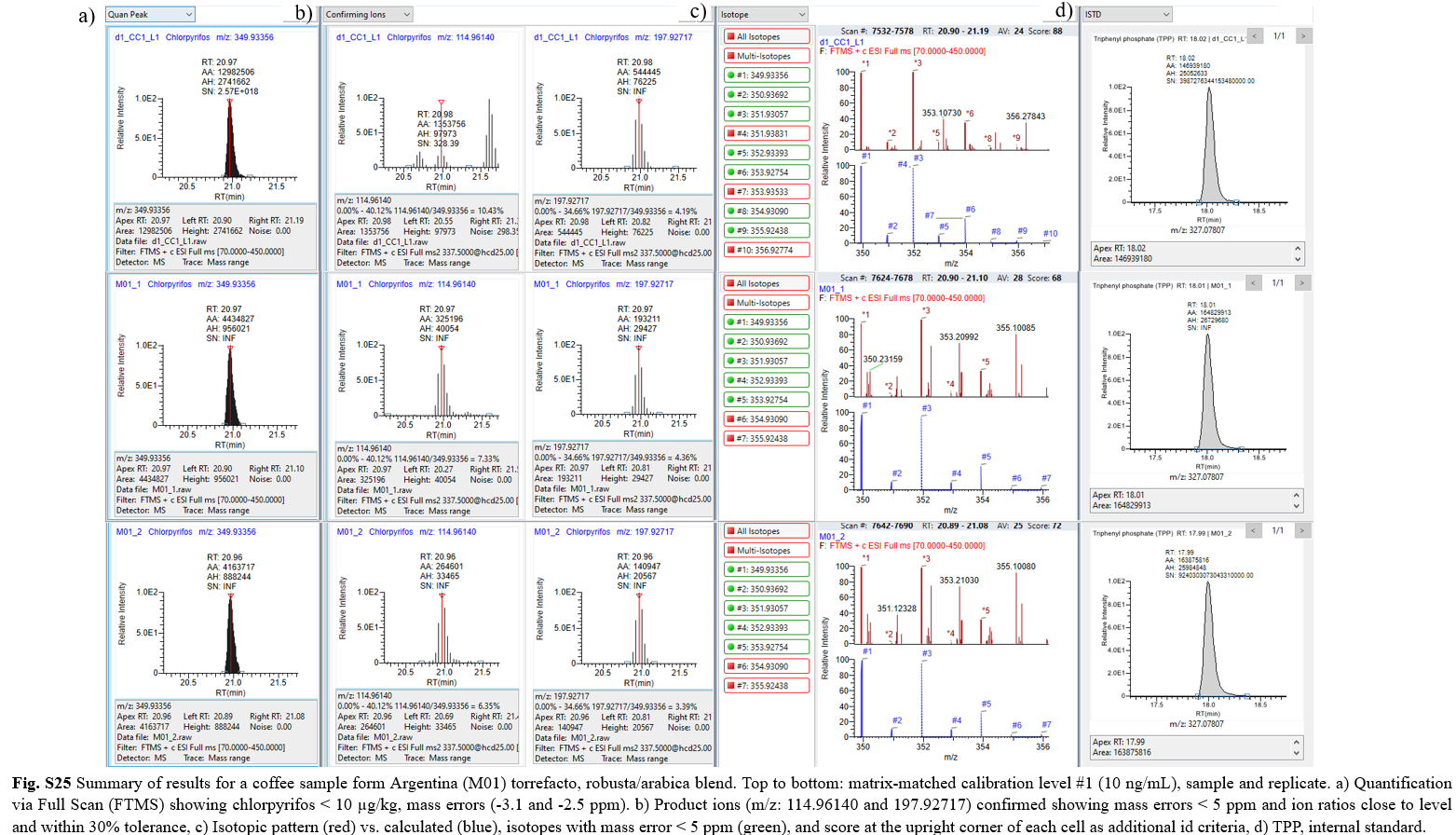
Figure S25

Figure S26


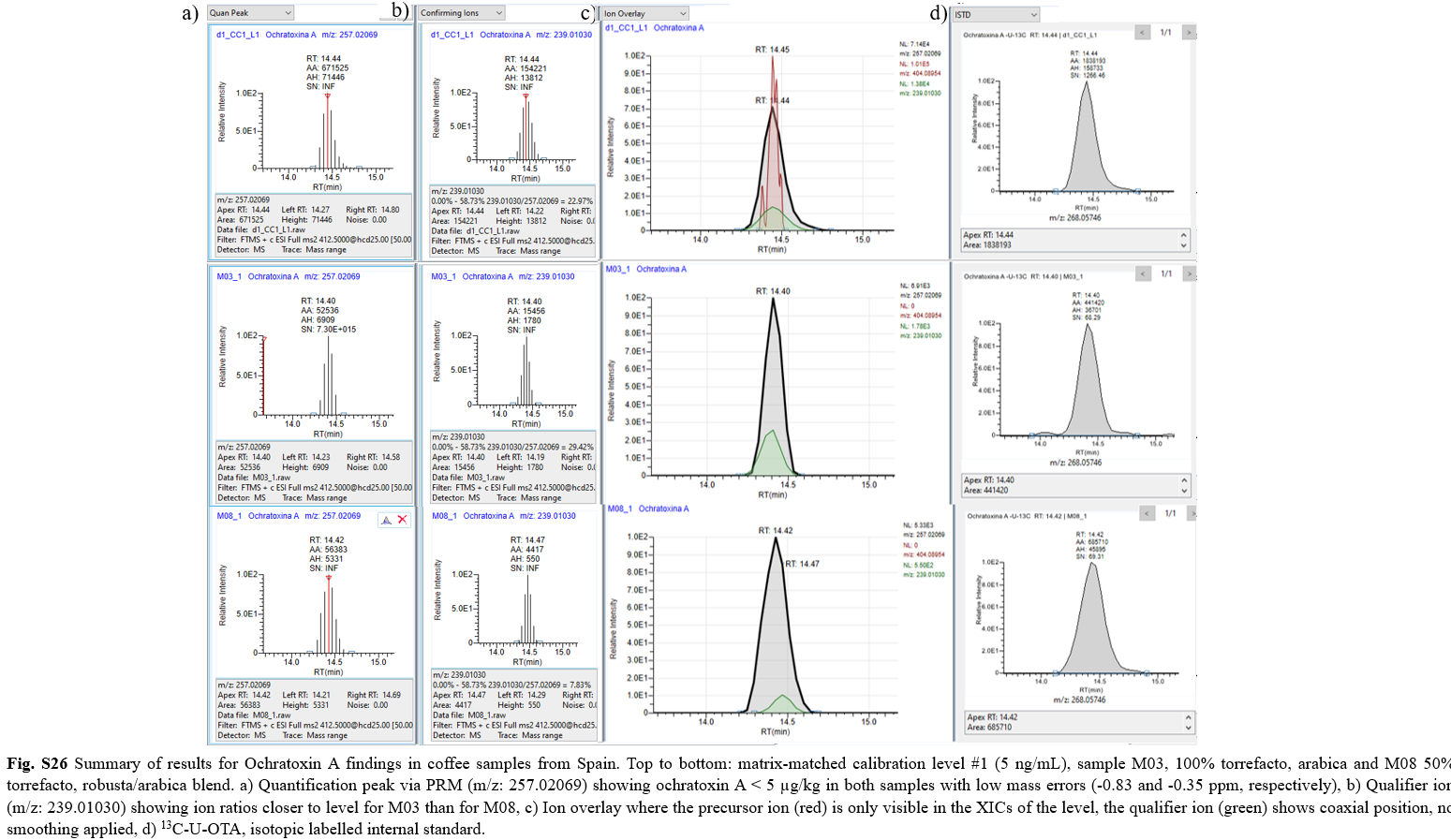


"This page is intentionally left blank."
